# Relative Bioavailability of Trace Minerals in Production Animal Nutrition: A Review

**DOI:** 10.3390/ani12151981

**Published:** 2022-08-04

**Authors:** Laurann Byrne, Richard A. Murphy

**Affiliations:** Alltech Bioscience Centre, Summerhill Road, Dunboyne, A86 X006 Co. Meath, Ireland

**Keywords:** organic trace mineral (OTM), biological availability, relative bioavailability, copper, iron, manganese, zinc

## Abstract

**Simple Summary:**

This is a comprehensive review containing the most up-to-date information on the relative bioavailability of selected trace minerals (copper, iron, manganese and zinc) used in ruminant, poultry and swine nutrition. Inorganic and organic forms of the trace minerals are included, and the differences between the product types are highlighted. Building on previously published tables and data, this review incorporates studies on newly developed products and concepts not previously discussed. Extensive data tables are included, providing a valuable reference guide. Methods to calculated relative bioavailability of the minerals are discussed and reasons for potential variance are noted. Detailed background information on uptake mechanisms to aid understanding of mineral transport is also contained in the current review.

**Abstract:**

The importance of dietary supplementation of animal feeds with trace minerals is irrefutable, with various forms of both organic and inorganic products commercially available. With advances in research techniques, and data obtained from both in-vitro and in-vivo studies in recent years, differences between inorganic and organic trace minerals have become more apparent. Furthermore, differences between specific organic mineral types can now be identified. Adhering to PRISMA guidelines for systematic reviews, we carried out an extensive literature search on previously published studies detailing performance responses to trace minerals, in addition to their corresponding relative bioavailability values. This review covers four of the main trace minerals included in feed: copper, iron, manganese and zinc, and encompasses the different types of organic and inorganic products commercially available. Their impact from environmental, economic, and nutritional perspectives are discussed, along with the biological availability of various mineral forms in production animals. Species-specific sections cover ruminants, poultry, and swine. Extensive relative bioavailability tables cover values for all trace mineral products commercially available, including those not previously reviewed in earlier studies, thereby providing a comprehensive industry reference guide. Additionally, we examine reasons for variance in reported relative bioavailability values, with an emphasis on accounting for data misinterpretation.

## 1. Introduction

The main objective of this review was to compile an up-to-date reference of relative biological values for both inorganic and organic mineral products used in animal nutrition which enables readers to compare and contrast products. Newer concepts in the area of mineral bioavailability are discussed and novel commercial product types developed in recent years have been included. An important justification for conducting the review was to show how easily data can be misrepresented and the extensive differences in bioavailability between commercially available trace mineral products. The importance of understanding how relative bioavailability values are obtained cannot be underestimated, as it enables informed decision-making from a nutritional perspective when choosing trace mineral products for dietary inclusion.

For the present review, we adapted the Preferred Reporting Items for Systematic Reviews and Meta-Analyses (PRISMA) statement, designed for clinical trials, to the systematic review of animal studies due to the lack of a better and more standardised screening method [1]. An electronic-based search in the scientific libraries PubMed, Scopus, Web of Science and ScienceDirect was performed [2,3,4,5]. Searches comprised a combination of MeSH terms and keywords, applying truncation (*), quotes and field tags with BOOLEAN operators. Keywords included: biological avail*, bioavail*, RBV, Trace element, micronutrient, iron, copper, zinc, manganese, organic trace mineral, OTM, inorganic trace mineral, ITM, animal feed, diet, dietary supplements, animal, chelat*, proteinate, amino acid. MeSH terms comprised the following terms: chelating agents, biological availability, nutritive value, trace elements, micronutrients, copper, zinc, iron, manganese, animal feed, animal nutritional physiological phenomena, dietary supplements, animals, animal nutrition sciences. Initial searches retuned 113,348 results and after final refinements, 328 peer-reviewed papers were selected for consideration based on relevance. Results were screened based on their titles, abstracts, and full text availability according to our inclusion criteria: (1) production animal studies; (2) relative bioavailability evaluation; and (3) copper, iron, manganese and zinc mineral studies. All non-English publications were excluded, and filters were applied to restrict the results to peer reviewed studies. Proceedings of scientific meetings identified by topical relevance and regulatory documents, such as those published by the European Food Safety Authority (EFSA), were manually searched to identify additional data. Searches were limited to studies published from 1991 onward to coincide with the EU acceptance of organic trace minerals in feed additives although some earlier data on inorganics is also reported based on inclusion in a previous publication which was incorporated into the review tables [6].

## 2. Trace Minerals in Animal Nutrition

“Trace mineral” is the term used to describe nutritional elements added to production and companion animal diets in micro quantities [7]. They are involved in structural, physiological, catalytic, and regulatory functions in animals and their inclusion in animal diets is necessary for a multitude of reasons. Diets may not contain adequate amounts of specific minerals to meet animal requirements, minerals in feed may not be in a form that is biologically available, or anti-nutritional factors may reduce the total proportion of the nutrient in a feedstuff that is available for use in normal body functions. Furthermore, mineral requirements vary over the lifecycle of the animal and tailored supplementation strategies are paramount to obtain optimum results in modern animal production systems. Of the trace minerals commonly included in dietary formulations, four were selected for the purposes of this review: copper, iron, manganese and zinc. Table 1 outlines the primary function of each of the aforementioned minerals, highlighting their importance in animal diets. Marked deficiencies are unlikely to occur in modern commercial production systems; however, marginal deficiencies could occur under certain conditions such as poor feed formulation or low feed intake. The occurrence and severity of mineral deficiencies are influenced by length of time that deficient diets are fed, prior mineral status, and physiological state [8].

The source of the mineral is of crucial importance. In addition to differing by type, OTM and ITM also differ greatly in terms of how well they are absorbed and utilised by an animal. Traditionally, diets have been supplemented with inorganic sources of the mineral elements but these were found to be inefficiently utilised. Research further highlighted that the low pH environment of the upper gastrointestinal tract reduced the digestibility of inorganic salts by causing dissociation, thereby leaving the minerals susceptible to various nutrient and ingredient antagonisms that impaired absorption [30]. As pH increases in the small intestine, minerals such as Zn and Cu can additionally form insoluble hydroxide precipitates, rendering them unavailable for absorption [31]. Over the last number of years, organic mineral sources have increasingly been used instead of inorganic sources due to their apparent benefits—the organic counterparts are better protected from unwanted interactions in the GI tract and have enhanced bioavailability.

### 2.1. Inorganic Trace Minerals

Inorganic trace mineral (ITM) salts such as oxides, carbonates, chlorides and sulphates have been traditionally used in commercial feed formulations to meet the mineral needs of production animals in correcting and preventing trace mineral deficiencies. Although the inorganic form is perceived as being an inexpensive way to supplement the diet, recent research has shown far greater return on investment when using organic trace minerals (OTM) in place of ITM and this topic is discussed further in Section 4.

Feed-grade sources of trace minerals can differ greatly in purity. The biological availability of minerals from these sources also varies, with sulphates usually having higher relative bioavailability values than oxides [32]. Overall, the bioavailability of ITM are limited and high doses are needed to fulfil animal requirements which often results in an imbalance of nutrients and potential toxicity issues [33]. The concept of bioavailability is discussed in detail in Section 3.

Often, wide safety margins for mineral levels are permitted in feed formulation in an attempt to counteract dietary antagonists or to allow the mineral to act as a growth promoter [34,35]. Legal limitations can vary between regions with some permitting higher levels of supplementation than others [36]. When such high volumes are ingested, saturation of cellular metal binding proteins can occur, resulting in an increase in free ionised metal concentrations which can cause tissue damage. Toxic effects vary depending on the specific trace element in question, the total amount of that element in the diet, the age and condition of the animal and the presence or absence of certain other dietary components [33,36,37]. The toxic effect of a trace element can also be the cause of a secondary deficiency of another trace element.

The pathologies associated with Cu, Fe and Zn toxicity are often the result of damage to lipids in cell membranes leading to cell lysis. While pigs are highly tolerant to dietary Cu, which is often supplemented in excess as a growth promoter, sheep are far more susceptible to chronic Cu toxicity and supplementation is restricted to 15 mg kg^−1^ DM in the EU [38], although different breeds are thought to be more tolerant due to genetic differences [30]. Cattle were traditionally thought to be relatively tolerant to Cu accumulation, but with intensive systems for rearing now commonplace, Cu toxicity has been reported [39,40]. Furthermore, issues surround the use of sacrificial Cu in an attempt to avoid deficiencies due to high Mo levels in forages. The Mo binds to Cu in the rumen, together with S, to form thiomolybdates that render the Cu unavailable. Incidences of Cu toxicity have arisen from this management practice previously. Acute Cu toxicity in cattle can cause severe haemorrhagic gastroenteritis and congestion of the liver, kidneys and spleen, while chronic Cu toxicity can result in icterus, an enlarged spleen, and hepatic and renal necrosis [41,42,43,44].

Continuing with ruminants as an example, toxic effects in cattle and sheep associated with chronic high Fe intake include decreases in key performance indicators such as feed intake, weight gain and feed efficiency [33,45]. Enteritis, liver necrosis, icterus and haemoglobinuria have also been reported [41]. High Fe concentrations can also decrease absorption of other essential nutrients such as P, Mg, Se and Cu [46,47].

Manganese has a low potential for toxicity due to its poor intestinal absorption and efficient biliary elimination [48,49], but it can interact with several other dietary nutrients such as Zn and Fe by competing with Fe for intestinal absorption sites [50] or reducing tissue concentrations of Fe and Zn [51].

As with Fe, excess Zn can cause decreases in feed consumption, feed efficiency and weight gain. Zinc has also been shown to decrease Cu absorption and clinical manifestations in one study in sheep included inappetence, loss of condition, diarrhoea with dehydration or subcutaneous oedema, profound weakness and jaundice [52,53]. In cattle, toxicity from Zn can result in lesions of gastroenteritis, renal and liver necrosis [41].

In addition to the toxic effects in animals, another concern is the impact on plants and microorganisms [54]. In recent years, there has been increased awareness of the impact of environmental pollution from excreted minerals often caused by intensive animal feeding operations and the low retention rate of ITM [55,56,57]. Authorities have taken action and set maximum permitted levels for mineral concentrations in feed to protect the consumer, animals and the environment and continue to do so [58]. As such, it is imperative that the minerals that are supplemented are utilised in the most effective manner. Enhancing mineral utilisation is one of the most effective ways to ensure cost savings, improve animal health and reduce environmental impact.

### 2.2. Organic Trace Minerals

Several different types of OTM are commercially available, based on the type of ligand (amino acid, peptide, polysaccharide or organic acid) used to bond with the mineral. Functionality and pH stability differ between the products formed, yet all are still grouped together under the broad “OTM” term. Products such as amino acid complexes, amino acid chelates, polysaccharide complexes and proteinates have been shown to have different mineral binding properties and different pH stabilities based on their respective production processes [59]. Table 2 outlines the different classes of OTM and the further variation that exists between the Association of American Feed Control Officials (AAFCO) and the European Union (EU) definitions. Classes which are equivalent to each other have been grouped together.

There are several proposed theories for the enhanced mineral availability of chelates and complexes of minerals with organic ligands. Complexing minerals with organic components may increase the passive absorption of minerals in the intestine by reducing the interaction between the mineral and other potential chelators in the intestinal lumen and thus prevent the formation of insoluble complexes with substances such as hydroxides, carbonates, phosphates, oxalates and phytates, which would render the mineral unavailable for absorption [60,61,62].

Another proposed explanation is that complexing the mineral with an organic component may increase the water and lipid solubility of the mineral which may enhance passive absorption of the mineral. Complexing a mineral with an organic component may also result in a more favourable water–lipid partitioning coefficient that favours absorption over a wide range of pH values [63].

Absorption of OTM may also be affected by changes in molecular weight, geometry, charge density and size of the complex or chelate formed, that could result in different affinities of the mineral for binding sites. Additionally, differences in dissociation rates of the mineral from the organic group to which they are bound, and differences in mineral-chelate solubility are known to affect absorption [64]. Furthermore, the strength of the bonds between the organic ligands and the mineral on formation of a complex or chelate can prevent dissociation as it passes through the digestive system and enhance biological availability of the mineral [59].

### 2.3. Mineral Uptake Mechanisms

Most absorption of trace minerals occurs in the small intestine, primarily in the duodenum, although absorption can occur anywhere along the GI tract [65,66]. Copper and zinc can also be absorbed in the rumen [65,66,67]. In poultry, the proventriculus is also a potential site for absorption [65].

Several pathways exist for absorption of ITM and OTM. The homeostatic control of mineral uptake is covered extensively in Section 2.3.1 and Section 2.3.2 and details a general model for absorption and resorption of inorganic minerals. With respect to organic trace elements, multiple studies have reported that organically bound trace minerals may be absorbed via amino acid or peptide transport pathways more effectively than through general mineral uptake pathways, which could explain their enhanced use [68,69,70,71,72,73]. With that in mind, several uptake mechanisms for OTM are outlined here but general homeostatic control mechanisms will also apply for their ultimate control.

The transport of amino acids into the cytoplasm occurs via functionally and biochemically distinct amino acid transport systems that have been defined on the basis of their amino acid selectivities and physico-chemical properties [74]. Each amino acid transport system adapts to the environmental conditions by choosing a coupling mode to achieve the affinity required for certain physiological conditions [75,76,77]. Amino acid transporters are categorised into at least 17 distinct classes [75]. Neutral amino acids are considered to be mainly transported by three systems: A, ASC and L [78]. Amino acids with short, polar, or linear side chains, such as L-alanine and L-serine are mainly transported by systems A and ASC. Large, branched and aromatic amino acids, such as L-tyrosine mainly enter cells via system L [79]. Species differences exist in the site of amino acid absorption and individual amino acids are not absorbed with equal efficiency—competition for transport is greater among amino acids for which a carrier has a greater affinity [80]. The transport of amino acids by intestinal enterocytes occurs by simple diffusion, facilitated diffusion (Na^+^-independent) and active transport (Na^+^-dependent) [81]. Brush border and basolateral membranes are crossed by amino acids, and di- and tripeptides by passive (facilitated or simple diffusion) or active (Na+ or H+ co- transporters) pathways [82]. Free amino acids use either passive or active transport systems, whereas di- and tripeptides use mainly active ones [82]. The relative significance of each route is highly dependent on the concentration of the substrate present [80]. Competitive inhibition from free amino acids is another factor to consider. A 2017 study, assessing the uptake of Zn provided by Zn-amino acid complexes, found a highly significant inhibitory effect on the increase in intracellular Zn levels after application of Zn-Glu, Zn-Lys and Zn-Met in the presence of Glu, Lys and Met respectively [71]. The same study noted uptake of Zn into cells was faster by the inorganic source of Zn tested (ZnCl_2_) compared to most of the Zn-amino acid complexes after 30 min but similar levels of absorption were observed after 120 min [71]. Other authors also found similar results where the uptake of Cu-amino acid complexes was lower compared to the free form of Cu in solution but the amino acid complex form facilitated Cu absorption in Caco-2 cells [83].

Animal diets are often supplemented with L-Met, DL-Met, or a hydroxyl analogue; DL-2-hydroxy-4-(methylthio)butanoic acid (DL-HMTBa), which is analogous to lactic acid [84]. Not only are the metabolism and use mechanisms different for these Met sources; they also differ in their absorption mechanisms [85]. For instance, as HMTBa is a precursor without an amino group, it is not absorbed by AA transporters, but rather by sodium-dependent and sodium-independent monocarboxylate transporters such as MCT1 [85,86,87]. As it is a racemic mixture with D- and L- enantiomers, differences in uptake mechanisms are not unexpected. This molecule has also been used for trace element conjugation and the complexes formed will be reliant on monocarboxylate transport pathways rather than amino or peptide transport mechanisms.

Short chain fatty acids such as acetate, propionate and butyrate were found to use a carrier mediated transport system specific for monocarboxylic acids such as MCT1 in addition to a non-electrogenic SCFA_-_/HCO_3_^−^ antiporter [88,89,90].

Previous work on peptides supported the theory that their rate and extent of absorption is greater than that of free amino acids and that independent transport systems for peptides exist [80,91,92,93,94,95,96,97,98]. The usual routes of peptide absorption include passive transcellular diffusion, carrier mediated transport by the proton-dependent peptide transporter, PepT1 for di- and tripeptides, vesicle-mediated intracellular transport of oligopeptides (transcytosis), and paracellular transport across the intestinal epithelium [99,100]. Once the peptide-mineral complex reaches the small intestine, it can either be absorbed intact via the usual peptide absorption mechanisms, or the mineral can be dissociated from the complex and absorbed alone. As homeostasis is tightly controlled at the cellular and organismal level, the mineral is bound by a chaperone protein following dissociation to prevent subcellular damage occurring. One example is cellular Cu metabolism, which is modulated within cells by a host of cytosolic chaperones which control Cu trafficking [101]. The SLC31 (CTR) family of Cu transporters is a major gateway of Cu acquisition in eukaryotes, ranging from yeast to humans [102]. Other examples include the divalent metal transporter 1 (DMT 1), a member of the proton coupled metal ion transporter family [103]. Copper may also be sequestered within cells by metallothionein (MT) which is a Cu- and Zn-binding protein. Uptake at both adequate and suboptimal mineral levels is discussed in greater detail in Section 2.3.1 and Section 2.3.2.

A comprehensive review by Goff (2018) suggests that, when fed at high concentrations, many minerals can use paracellular absorption mechanisms, where the mineral diffuses across the tight junction, or solvent drag where the mineral moves with the bulk flow of water between intestinal epithelial cells to enter the blood. Minerals complexed to various dietary substances such as amino acids and peptides can also be absorbed via solvent drag, provided they are soluble in the unstirred water layer over the tight junction and generally less than 3.5 kDa in size. At lower dietary concentrations, the body primarily relies on transcellular absorption which requires transport proteins to move the mineral across the apical membrane [66].

#### 2.3.1. Adequate Levels

##### Copper

In monogastric species, Cu is primarily absorbed across the stomach and small intestine by a transcellular process [104]. Transporters and proteins involved in the regulation of Cu in cattle have been characterised by Fry et al. [105]. Paracellular Cu absorption by diffusion is unlikely due to the potential difference across the tight junction, created by the high Na^+^ content of the interstitial space, being too highly positively charged. Paracellular absorption via solvent drag could be a minor contributor [66]. Cu and Zn are not free ions at the neutral pH of the intestine, but rather are often associated with low molecular weight binding ligands which enhance mucosal uptake of these trace minerals [106,107].

**Figure 1:**
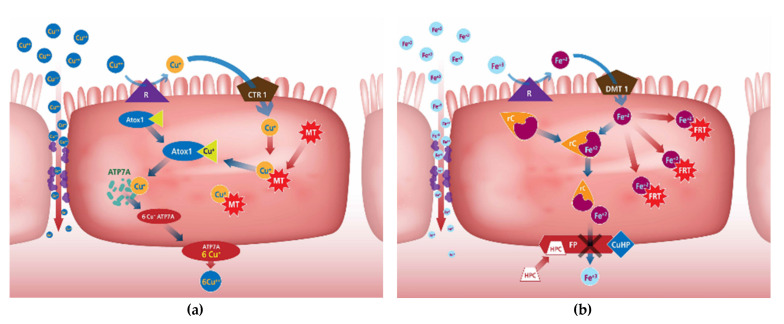

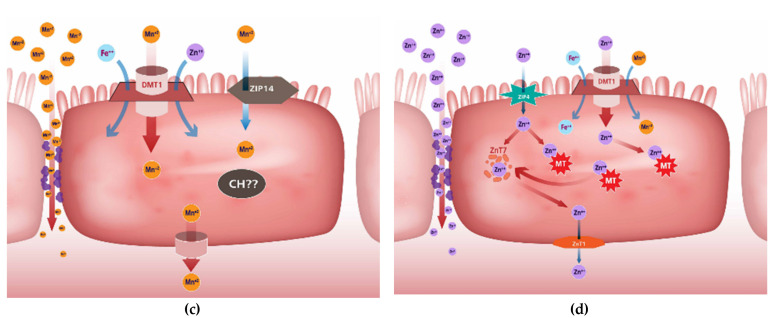
Cu (**a**), Fe (**b)**, Mn (**c**) and Zn (**d**) trace mineral uptake mechanisms at adequate mineral status. Adapted from Goff [67].

Figure 1a describes cellular Cu homeostasis which is regulated primarily by two transporters: the Cu transporter 1 (CTR1; also known as SLC31A1), which controls the uptake of Cu, and the Cu-extruding ATPase ATP7A, a recognised retromer cargo recognition complex [108]. Copper transporter 1 (CRT1) is the major transporter involved in cellular uptake of Cu by intestinal and other mammalian cells. Three different chaperone proteins have been identified—Cu chaperone protein (CCS) transports Cu to Cu/Zn superoxide dismutase in the cytosol, Cox17 transports Cu to proteins in the mitochondria that transfer Cu to cytochrome c oxidase in the inner mitochondrial membrane, and Atox1, which transports Cu to Cu ATPases in the trans-Golgi network [109,110]. If the body has adequate Cu stores, the enterocytes begin to produce MT which binds to Cu ions entering the cell in preference to the Atox1 chaperone. Much of the MT-bound Cu may be trapped in the enterocyte, which when it dies, is sloughed off and excreted with the faeces. High Cu status also reduces the amount of CTR1 in the apical membrane [66].

##### Iron

Iron in the ferric form (Fe^3+^) is poorly absorbed from the intestinal tract. The ferrous (Fe^2+^) form usually becomes bound to a chelator during digestion such as histidine, mucin, or fructose which enhances Fe absorption by solubilising the Fe ion and protecting it in the ferrous state [107,111]. Formation of Fe–amino acid complexes may allow the Fe to use amino acid transporters to move across the intestine [112]. Iron can also complex with gastric secretions allowing it to remain soluble at the more neutral pH environments of the intestine [111]. A chaperone protein, poy (rC)-binding protein-1 (rC), can be used for transport to the basolateral membrane (Figure 1b). Divalent metal transporter 1 (DMT1) is the major transporter of Fe across the apical membrane and is specific for Fe^2+^. A ferrireductase (R), such as duodenal cytochrome B (DcytB), on the apical surface of enterocytes reduces Fe^3+^ prior to transport [113,114].

When Fe stores are adequate, the amount of DMT1 is reduced. The enterocytes produce ferritin (FRT), which binds and sequesters the bulk of the Fe^2+^ crossing the apical membrane. Hepcidin (HPC), a hormone produced in the liver, binds to ferroportin (FP), a basal membrane Fe transporter, blocking its ability to transport Fe out of the cell. Expression of hepcidin is regulated by liver Fe stores and can signal the small intestine to down regulate Fe absorption [66,113,115,116].

##### Manganese

Two Mn transport proteins have been well characterised using in vitro and rodent models: the cellular Mn importer, DMT1 (Figure 1c), and cellular Mn exporter, ferroportin 1 (FPN1) [117]. Manganese absorption does not appear to require the metal transporter DMT1 at adequate levels. Both hepatic ZIP14 and ZNT10 are necessary for effective secretion of Mn into the bile to prevent Mn accumulation by tissues [118]. In broilers, specific Mn transporter proteins exist within the duodenum and jejunum but are of limited capacity. The ileum of broilers is able to absorb Mn through a non-saturable process, suggesting that the absorption is occurring paracellularly across the tight junctions when Mn concentrations are high [66,119]. Uptake of Mn from amino acid complexes is likely not only mediated through transporters specific for ionized Mn^2+^, but also through cationic amino acid transporter (CAT) 1 and CAT 2 systems in addition to system b^0,+^ amino acid transporters [117].

##### Zinc

Intestinal Zn absorption occurs primarily in the small intestine by a transcellular transport process (Figure 1d). The transporters required for Zn absorption are also present in the colon [120]. Intestinal Zn absorption is mainly mediated by the Zrt-, Irt-like protein (ZIP)4 (solute carrier (SLC)39A4), which imports ionic Zn from the lumen into enterocytes, and ZnT-1 (SLC30A1), which is a basolateral membrane protein exporting Zn on the basolateral side of enterocytes into the portal blood [121,122,123]. ZIP4 is considered to be the major intestinal Zn import transporter [109,124].

With adequate Zn levels in the body, the amount of ZIP4 in the apical membrane is downregulated and the enterocytes begin to produce high amounts of MT to bind any additional Zn^2+^. As with Cu, upon cellular death, Zn bound MT is excreted [107]. Because Zn and Cu are regulated by the same metalloprotein, one mineral can reduce the absorption and/or transfer of the other mineral [107]. Paracellular absorption of Zn is also known to occur with high Zn concentrations [125].

#### 2.3.2. Suboptimal Levels

##### Copper

**Figure 2:**
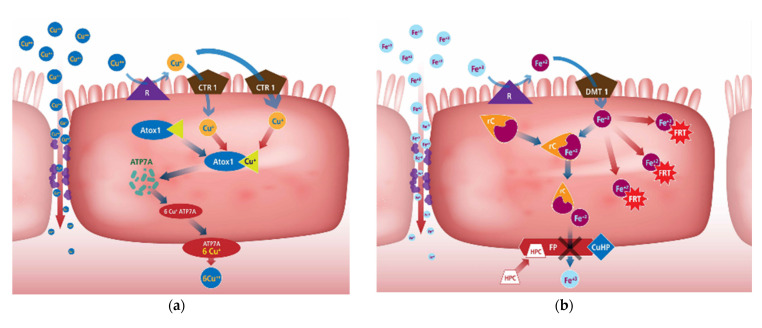

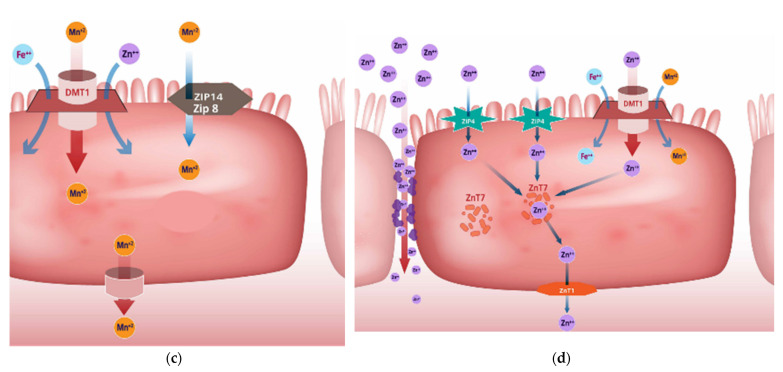
Cu (**a**), Fe (**b**), Mn (**c**) and Zn (**d**) trace mineral uptake mechanisms at adequate mineral status. Adapted from Goff [67].

Brush border Cu metalloreductases (R) convert dietary Cu^2+^ to Cu^+^ and a Cu transporter protein (CTR1) facilitates diffusion of the Cu^+^ across the apical membrane where it becomes bound to a Cu chaperone protein (Atox1) [118,126]. Subsequently, Atox1 shuttles the Cu^+^ to the Golgi apparatus, where it is transferred to a Cu transport protein (ATP7A) capable of holding six Cu^+^ ions that is within the membrane of a Golgi transport vesicle (Figure 2a). It has also been suggested that DMT1 acts as a minor pathway [127] and a Cu/Cl cotransport mechanism has also been proposed [128].

##### Iron

At suboptimal levels, the amount of DMT1 in the apical membrane is upregulated which can move Fe^2+^ across. Ferrireductase (R) can convert dietary Fe^3+^ to Fe^2+^ for absorption (Figure 2b). Once Fe^2+^ crosses the apical membrane, it is picked up by a chaperone protein, poly (rC)-binding protein-1 (rC), for transport to the basolateral membrane. Ferroportin (FP) then pumps the Fe^2+^ across the basolateral membrane. Before the Fe^2+^ enters the interstitial fluid, it is converted to Fe^3+^ by Cu-hephaestin (CuHP), linked to the FP transporter [66].

##### Manganese

Transcellular absorption of Mn^2+^ involves the use of divalent metal transporters such as DMT1, ZIP8 and ZIP14 to move Mn (and other metals) across the apical membrane (Figure 2c) [66,119].

##### Zinc

Transcription of the Zip4 gene can increase Zn deficiency and contributes to homeostatic upregulation of Zn transport at the apical surface [118]. Other ZIP transporters have been identified (ZIP 11 and ZIP 14) that may play a minor transport role [129]. Zinc can also use DMT1 to cross the apical membrane, though it must compete for binding sites with Fe and Mn (Figure 2d). Chaperone protein 2, in addition to 4, 5, 6 and 7, move Zn^2+^ to the basolateral membrane where the Zn intestinal transporter 1 (ZnT1) moves the Zn^2+^ into the interstitial fluid prior to it being bound to albumin [129].

## 3. Bioavailability

Numerous definitions of bioavailability exist [130,131,132]; but in terms of trace minerals, bioavailability may be defined as the proportion of an ingested mineral that is absorbed, transported to its site of action and converted to the physiologically active species [130]. Other terms which have been used include “biological availability”, “bioactivity”, “biopotency” and “bioefficacy” [6]. Many factors affect bioavailability [6,59,133,134,135] and although not an exhaustive list, the main contributors to variance are outlined in Table 3.

### 3.1. Evaluation of Bioavailability

Several methods to evaluate bioavailability exist and, in general, the bioavailability of a mineral element is determined relative to its functional availability from a standard source [6,67,136]. Bioavailability methods suitable for one element may be totally unsuitable for another element [134]. Use of a standard source allows expression of bioavailability in terms of relative biological availability [132]. This approach results in a number referred to as the “relative bioavailability value” or RBV. As bioavailability is an experimentally determined estimate which reflects the absorption and use of the mineral under conditions specific to the individual test [137], true differences in bioavailability can be masked by any number of the factors outlined in Table 3. By way of example, a selection of the parameters outlined in the evaluation section of Table 3 are highlighted in the following sub-sections to further emphasise their effect on published RBV.

#### 3.1.1. Reference/Standard Source

The reference standard used in bioavailability studies should be a highly available source [131]. It has been unequivocally demonstrated that Cu from CuO is poorly available to poultry, pigs and cattle [137,138,139,140]. Therefore, CuO should not be used as a standard for evaluation of bioavailability of organic Cu sources. Similarly, in another study, bioavailability estimates of four feed-grade ZnO sources ranged from 22% to 93% compared to a ZnSO_4_.7H_2_O standard set at 100% [141]. Manganese sulphate monohydrate is generally used as the standard reference for assessment of Mn bioavailability, but true absorption of the Mn in this compound is 2% to 8%, depending on the species and the diet to which Mn is added [23,142].

Commercial availability of specific forms of mineral sources used in feed production is another factor of importance. It is important that studies select readily available forms that are commonly used as the choice of mineral standard can greatly impact results, making certain products appear more (or less) bioavailable than they actually are. Many chemical forms of minerals exist and choosing one as a standard that is not commonly used as a production source may distort results. Taking sulphates, which are often recommended as a standard source, as an example: the pentahydrate form of Cu(II) sulphate is the most commonly used commercially available form. For Fe(II) sulphate both the hepta- and mono- forms are frequently used in commercial production. The monohydrate form of both Zn and Mn sulphate is the most commercially relevant. Choosing alternative sulphate forms that are not as common, diminishes the value of the calculated result and makes comparisons with other research work difficult.

#### 3.1.2. Model Selection

Most statistical assays of bioavailability use regression models and in recent years, the majority of estimates for the relative bioavailability of different mineral sources have been commonly obtained through slope-ratio assays [131]. In this assay, diets with graded levels of mineral are formulated, and responses indicative of mineral status of the animals are evaluated. The slope of the regression line obtained from animals fed the test source of mineral is compared with that from animals fed a reference source [131,143,144]. It is assumed that there is a straight-line relationship between the independent and dependent variables, x and y, for both test and standard nutrient sources. The ratio of slopes of the regression lines gives RBV, hence the name “slope ratio” assay. Other assays include parallel lines, three-point, mean ratio and standard curve. If the regressions of y on x are linear and the intercepts are equal, then the model for the slope ratio assay fits the data; however, if the regressions of y on log_10_x are linear and have equal slopes, then the model for the parallel lines assay is appropriate and the corresponding estimate of RBV should be used [131]. The other assay designs—three-point, mean ratio and standard curve should only be used if certain assumptions are known to be valid regarding the relationship between y and x. From the tables in this review, the three-point assay is commonly found in older studies and as the name implies, relies on only three design points to estimate RBV and is extremely dependent on validity of the assumptions for either the slope ratio or parallel lines assay [131]. For example, if assumptions for the slope ratio assay are known to hold, that is, if regressions of y on x are known to be linear with equal intercepts, then the three-point design ordinarily would include one point at x = 0 supplemental nutrient to define the intercept, and two other points obtained at a positive value for x for both standard and test sources. Lines are then struck by joining the intercept with each of the two points resulting from positive x. The ratio of slopes of these two lines produces the RBV estimate as in the case of the slope ratio assay [131].

#### 3.1.3. Choice of Response Criteria

The choice of response variable is another parameter of importance to highlight. Measuring the deposition or storage of minerals into selected tissues such as tibia or plasma for Zn, liver for Cu or tibia for Mn was the most common output in trace mineral relative bioavailability experiments [30]. More recently, the use of mineral-responsive biomarkers, such as changes in gene or protein expression, or the activity of a mineral-dependent enzyme, have become more common [145,146,147]. Previous studies have outlined metacarpal, coccygeal and plasma Zn percentages differing enormously [133,148,149]. One study reported bioavailability estimates of 67%, 70% and 87% for ZnO, 24%, 38% and 79% for Zn-Lys and 60%, 84% and 95% for Zn-Met, when metacarpal, coccygeal and plasma Zn respectively were used as response variables in weaned piglets with the sulphate form as reference [148]. Other studies reported bone as the most sensitive response variable in chicks for use as a bioassay criterion for determining Zn bioavailability [25,150,151]. For Cu, it has been reported that Cu concentration in liver is the best biomarker for diagnosing Cu disorders in animals [152] and several reasons were suggested as to why serum/plasma Cu concentrations are not as reliable, including ceruloplasmin concentrations being highly influenced by factors other than dietary Cu (particularly by inflammation as it is an acute phase protein); ceruloplasmin concentrations do not increase once the animal reaches an adequate Cu status and, in situations where important dietary sulphur and molybdenum interactions occur, insoluble Cu thiomolybdates in the blood do not reflect a real Cu deficiency situation [14]. A previous study provided ranking of criteria for each mineral response parameter, again demonstrating the potential variability of bioavailability estimates [135].

#### 3.1.4. Comparison of Mineral Sources

It is important to note that not all OTM are equally stable across the range of pH values encountered in the gastrointestinal tract and, therefore, chelation with differing ligands will not necessarily increase the bioavailability of a given mineral to the same extent [153,154,155,156]. Conditions such as pH can also impact the solubility of peptide-mineral complexes and previous research noted Fe salts exhibited a low solubility of 5.9% at pH 6 and pH 8, whereas peptide-Fe complexes retained over 90% Fe solubility under the same conditions [157]. The low solubility of free Fe at pH levels such as those encountered in the intestine is noted to be a significant factor that contributes to its poor availability [158]. However, although solubility of the peptide-mineral complex is important from a bioaccessibility perspective, it does not guarantee absorption of the minerals. Caetano-Silva et al. found that metal complexes with low molecular weight (<5 kDa) peptides exhibited significantly higher in-vitro bioavailability, albeit at similar levels of bioaccessibility as measured by solubility [157].

To highlight the difficulties with assessing and comparing the RBV of mineral sources, a specific example can be found in an older publication [159]. Using GLM procedures, a meta-analysis was carried out on publications between 1986 and 2010 using broiler and piglet data to investigate the bioavailability of inorganic and organic mineral sources. Results concluded the bioavailability of organic Zn relative to inorganic Zn sources was not different and ranged, depending on the variable, from 85% to 117% but never significantly different than the control (*p* > 0.05) [159]. However, the data must be kept in context. For the meta-analysis, inorganic Zn was either the oxide or sulphate form with the exception of three broiler observations using Zn acetate. Organic Zn had glycine, lysine, methionine, hydrolysed soy protein, hydrolysed protein or yeast protein as the ligand. Newer products have been developed over the years that were not included in the analysis and grouping all OTM products together when there are such differences in chemical characteristics between different OTM forms is not ideal. It is likely that some OTM will not have better bioavailability than ITM but, based on many of the publications cited in this review, others certainly do. The meta-analysis also noted there were some parameters missing in some of the experiments reflecting flaws in experimental design which can prevent detection of real differences. To compensate for this, values were estimated using the published feed ingredient formulation and the standard Zn contents of each ingredient from INRA-AFZ [160]. In cases where there was information missing with respect to the supplemented Zn level, it was calculated as the difference between total Zn and native Zn. The values used for the meta-analysis were not based on calculated RBV values but on Zn (mg kg^−1^) increase in a selection of response criteria [159]. Also, reviewing the studies chosen for the meta-analysis, 2 studies were unpublished and, therefore, not peer-reviewed at the time, and many did not directly compare OTM and ITM in the same paper. Those that did contain direct comparisons often showed the organic form was superior [161,162]. A later paper which referred to the meta-analysis also noted that the supplemental Zn levels in many of the experiments selected for the meta-analysis were beyond the linear response range, or above the tissue Zn breakpoint, which can minimize differences between sources [150,159,163,164].

### 3.2. Relative Bioavailability Tables

Incorporating results from previous studies and extending the search to include the most recently published data, a comprehensive set of tables has been compiled for this review to discuss this topic in greater detail. Key results across all production animals are included in the following multi-species tables (Table 4, Table 5, Table 6 and Table 7). Only the most frequently studied sources of mineral are included herein. The complete data set, including references, can be accessed in the Supplementary Tables S1–S17.

The sulphate form was used as the standard source to obtain the relative values except where otherwise specified although, as mentioned previously, not all of the sulphate sources have equal commercial relevance and the Supplementary Tables S1–S17 show that there were different sulphate sources selected for some studies.

Source type nomenclatures are those used by the respective researchers and may differ when referring to the same source. Often, the terms complex and chelate are incorrectly used interchangeably. Denticity, _Κ_, which refers to the number of atoms with which a ligand binds to a metal ion can be used to differentiate between the two. A monodentate ligand binds through a lone pair on a single atom forming a complex. Bidentate binding through lone pairs on two differ atoms or polydentate ligands with multiple binding sites can enable the formation of ring structures with the metal ion forming chelates (from the Greek chele, meaning “claw”). An important feature of metal chelates is their high stability due to the conformation in which the metal atom is held by coordinating groups. Due to their ability to form fused chelate rings, polydentate ligands have larger formation constants (which refer to the strength of complexation between a ligand and a metal) compared to monodentate ligands. This so called “chelate effect” is mainly due to the more favourable entropy change that occurs during complex formation involving polydentate ligands. A multitude of factors can affect the stability and subsequent absorption of both complexes and chelates, including the ligands involved in binding as outlined earlier in Section 2.2.

The wide range of relative bioavailability values can also be partly due to differences in selection of response criteria. Furthermore, real differences in bioavailability can be masked if source comparisons are not made on the linear portion of the dose-response curve [165].

### 3.3. Key Species Observations from RBV Tables

In most older studies, OTM have been shown to be at least as bioavailable, if not more so, than their inorganic counterparts, thereby allowing more mineral to be absorbed and increasing mineral status within the animal [150,167,168,169,170,171,172,173,174,175,176,177]. The tables reflect similar conclusions. Chemical characteristics considered important in predicting the bioavailability of chelated and complexed metals include the chelation effectiveness (strength of bonds between an organic ligand and a metal) and the percentage of organic ligand that remains bound to the metal under physiological pH conditions [178].

#### 3.3.1. Ruminants—Beef and Dairy

Organic trace minerals have been shown to have many benefits in ruminants including a positive effect on milk yield, milk quality, higher average daily gain (ADG), reduced incidences of respiratory issues, improved carcass characteristics and meat quality and higher reproductive efficiency [179,180,181,182,183,184,185,186,187,188,189,190,191]. When added to diets that have known antagonists such as Mo and S that can interfere with mineral absorption, OTM, and proteinates in particular, have been noted to have better bioavailability than ITM [174,175].

##### Copper Relative Bioavailability—Beef and Dairy

Cupric sulphate has primarily been used as the reference standard for comparative evaluation of the absorption and bioavailability of Cu. Recently, cupric sulphate pentahydrate has been most commonly used for experiments in ruminants, although variation within the sulphate standard source can still exist with the use of feed-grade sulphate standards in place of reagent grade sulphates. Liver and Plasma Cu are the two main response criteria identified from the tables (Table S1). However, differences exist between both, and choice of response criteriacan significantly impact RBV. In a previous study, the bioavailability of Cu proteinate relative to cupric sulphate had been calculated to be either 147% or 112% depending on whether liver Cu or plasma Cu was used as the response criterion [174,192]. Such findings are in agreement with several other studies stating that liver Cu levels, not plasma Cu levels, are a better indicator of Cu status and relative bioavailability between sources [193,194,195].

Copper deficiency is a concern for grazing ruminants in many countries due to both low concentrations of the mineral in forage as well as elevated amounts of antagonists such as molybdenum and sulphur which interfere with Cu use [192]. Interactions with Mo and S have been shown to influence the availability of Cu in numerous studies [68,177,196,197]. Kincaid et al. reported a higher bioavailability of Cu from Cu proteinate compared with Cu sulphate in calves fed diets containing molybdenum [174]. Du et al. found no difference in values between the proteinate and the sulphate form but acknowledged dietary Mo and S were not high enough in their study to affect Cu availability [72]. This ties in with other results contained in the tables that OTM perform better than ITM in the presence of dietary antagonists. Ward et al. found that Cu proteinate and Cu sulphate were equally effective at supplying Cu to cattle fed low dietary Mo. However, when the diet contained higher levels of Mo, Cu from the proteinate source was more bioavailable resulting in higher liver Cu concentrations [175].

Nockels et al. found Cu-Lys had higher RBV following stress induced by ACTH administration and feed and water restriction [166]. In contrast to the above, another study from Ward et al. found no difference in Cu bioavailability between Cu sulphate and Cu-Lys regardless of dietary Mo and S levels. Differences in chemical characteristics between the organic sources (lysine vs. proteinate) may in part explain the difference while response criteria also differed (apparent absorption vs. plasma Cu) [198]. A more recent study using Cu glycinate relative to feed-grade Cu sulphate found the organic form was more available than the inorganic sulphate when supplemented to diets high in S and Mo [199].

The high RBV of Cu from tribasic copper chloride (TBCC) observed in Table 4 may relate to the low solubility of Cu chloride in the rumen environment, which may reduce the potential for Cu to interact with molybdenum and sulphur [200], although RBV vary from a low of 86% to a high of 196% depending on the study. Such a wide range in estimated RBV of the same mineral source indicates the inherent difficulties associated with experimental estimation of bioavailability. Tribasic copper chloride and cupric sulphate actually had similar bioavailability in beef cattle and heifers when evaluated in diets that were low in molybdenum, but TBCC was more available when supplemented to diets high in Mo and S [196,197,201,202]. It has been suggested that higher bioavailability of some mineral forms is due to lower formation of insoluble complexes (thiomolybdates).

Another example of variation is due to manufacturing conditions which can produce very different products of apparently the same type. One particular study highlighted differences in the solubility of two Cu proteinates in water (75% and 10%) and in an acidic environment (99% and 87%), respectively [175]. The pH-dependent solubility of organic compounds could explain some differences in the experimental results. Higher bioavailability in comparison with Cu sulphate was found when feeding diets with high molybdenum and sulphur in Cu chelate [175] and Cu glycinate [199]; however, another experiment with high Mo and S showed similar bioavailability to Cu sulphate with Cu proteinate [203]. Van den Top summarised the results of a selection of bioavailability trials in 2005 with different Cu sources for ruminants and concluded that the evidence was not fully in agreement at that time. In the presence of low molybdenum concentration in the ration (concentration less than approximately 2 mg kg^−1^ DM), the differences in bioavailability between the different Cu sources appeared to be minor; however, in the presence of higher Mo concentrations (+5 to 7 mg kg^−1^ DM), Cu proteinate did have an advantage [204].

Copper carbonate had lower solubility in water but under acidic conditions was soluble (74%); however, it did not increase Cu in the liver in comparison with Cu sulphate even in a diet high in S and Mo [175]. Hemken et al. reported Cu proteinate to be more bioavailable than cupric sulphate in studies involving beef cattle [205]. Further studies by the same group in dairy cows demonstrated an increased hepatic Fe content in Cu proteinate—versus cupric sulphate—supplemented animals, suggesting the proteinate form did not interfere with Fe uptake and storage as might be expected with inorganic Cu sources [72]. In addition, it was found that Cu proteinate-supplemented cows had lower plasma ceruloplasmin activity than cows fed cupric sulphate even though plasma Cu was essentially the same for both groups suggesting the proteinate form may be absorbed via a different mechanism [72].

##### Iron Relative Bioavailability—Beef and Dairy

Most basal diets contain sufficient Fe to meet ruminant mineral requirements and, as such, there are very limited published RBV for Fe in cattle (Table 5 and Table S5) but for those that were obtained, ferrous sulphate was chosen as the standard source although some used feed-grade in place of reagent grade. Values in most cases were calculated using the three-point method and response criterion varied between studies. Ferric citrate gave the highest RBV of 107% compared to the sulphate standard value of 100% which was not statistically significant. Ferrous carbonate ranged from a RBV of 0% to 79% depending on response criterion chosen [206,207].

##### Manganese Relative Bioavailability—Beef and Dairy

Manganese is considered to be supplied in sufficient quantities in background feed and, as such, very little research has been carried out on this mineral for ruminants. Additionally, most ruminant mineral deficiencies are related to Cu and Zn and, consequently, Mn research is rarely focussed on.

##### Zinc Relative Bioavailability—Beef and Dairy

From the values obtained in Table 7 and Table S12 for Zn in cattle, the highest reported RBV was found from corn forage in a study by Neathery et al. from 1972 [208]. However, the standard source (^65^ZnCl_2_) and response criterion (intrinsic label) were not commonly selected parameters and, as such, the RBV obtained cannot be directly compared with later studies and serves to highlight just how varied RBV can be depending on the experimental parameters chosen. Organic minerals were not available for comparative purposes at the time of this study.

The highest RBV in studies using similar selection criteria were obtained using OTM in the form of Zn-Met and a Zn-polysaccharide complex. However, the RBV for both were obtained using ZnO as the standard as opposed to the sulphate form used for the others [209,210,211]. Considering only those measured against the sulphate form, there were very few differences between standard and source values in most cases with the exception of the chloride and carbonate forms which had nearly 50% lower RBV in plasma Zn calculated in one study [212]. From Table S12, it can be noted that the response criteria chosen varies greatly between studies making direct comparisons difficult.

Several more recent studies comparing Zn sources in cattle have often found advantages using a proteinate mineral form but did not have published RBV. Similar concentrations of Zn in plasma and tissue were found in cattle when supplemented at low levels but at higher levels of supplementation greater absorption and/or retention was observed from the proteinate form compared with sulphate [213]. Zinc proteinate was found to improve performance of finishing steers above that observed with inorganic Zn supplementation [214]. Results from another study in cattle observed similar absorption of Zn from Zn proteinate and ZnO, but with increased retention of Zn from Zn proteinate [215]. Milk yield was found to increase with supplementation of Zn in a proteinate form vs. oxide in a 2009 study examining the effects of level and form on dairy cow performance and health [216].

#### 3.3.2. Ruminants—Sheep

Relative bioavailability values for all four OTM were obtained for ewes and lambs (Table 4, Table 5, Table 6, Table 7, Table S3, Table S7, Table S10 and Table S16). Recent genetic improvement of sheep breeds common in extensive production systems can enable producers to accurately estimate specific mineral requirements respective of breed and production stage [217]. With certain breeds more susceptible to Cu toxicity due to low dietary Cu tolerance, deficiencies can sometimes be overlooked even though studies have reported issues affecting absorption. High dietary Mo, low dietary Cu:Mo ratios of <2:1, low Cu forage concentrations, dietary S-Fe-Mo antagonism, Cu concentration differences across forage species, phenological stages and uneven Cu distribution in plant tissues can all have an impact [15,217].

##### Copper Relative Bioavailability—Sheep

Ledoux et al. [135] estimated relative bioavailability values for inorganic Cu compounds in sheep by using an average of two experiments, which differs slightly from values reported on the same work in a later review which used different calculation parameters (values in parentheses): chloride 100% (96), acetate 93% (100), sulphate 142% (100), carbonate 121% (97) and oxide 35% (22) [138]. Using the slope ratio method to calculate RBV and a sulphate or chloride form as the standard, the highest RBV were found using OTM for sheep (Table 4 and Table S3).

Increasing dietary sulphur reduced Cu bioavailability by 30% to 26% in hypocupraemic ewes fed low molybdenum diets [144]. Spears cited data to show that at low ruminal sulphide concentrations, molybdenum may have little effect on Cu availability, whereas at higher ruminal sulphide concentrations Cu availability was significantly decreased [218].

Cheng et al. [219] reported that Cu-Lys and TBCC are of similar availability in lambs whereas Pott et al. [220] reported a Cu-Lys RBV of 68% compared with Cu sulphate. Pal et al. [221] concluded that ewes supplemented with organic (methionine-chelated) sources of Cu and Zn showed better feed conversion efficiency vs. the sulphate form, suggesting better nutrient use with bioavailability of 152% based on liver Cu.

Eckert et al. [222] also reported differences in the way Cu from Cu proteinate and Cu sulphate were metabolised by sheep. Copper from Cu proteinate resulted in greater ceruloplasmin activity than Cu from Cu sulphate, whereas ewes fed increasing levels of Cu from Cu sulphate deposited more Cu in the liver compared with ewes fed Cu proteinate.

##### Iron Relative Bioavailability—Sheep

According to the National Research Council, Ca, Mn and Fe have the greatest requirement during gestation, while all other minerals have a greater relative requirement in early lactation [223]. Published RBV in sheep are limited, but a 2001 study by Van Ravenswaay et al. compared the bioavailability of Fe from three feed-grade ferrous carbonates and a reagent grade ferrous sulphate (FeSO_4_.7H_2_O) [224]. In that particular study, overall average bioavailability estimates based on multiple regression slope ratios for three tissues (liver, kidney and spleen) were 55%, 0% and 20% respectively.

##### Manganese Relative Bioavailability—Sheep

As with Fe, very few published studies were found estimating RBV for Mn in sheep. Studies that did report Mn RBV values in sheep, or from which RBV were subsequently calculated, used the monohydrate sulphate form as a standard but differed in choice of standard grade (feed vs. reagent), test parameters and response criteria. A 1992 study compared a feed-grade Mn-Met complex and two feed-grade MnO sources using reagent grade MnSO_4_.H_2_O as the standard. Overall estimated RBV based on multiple linear regression coefficients of bone, kidney, and log-transformed liver Mn concentrations were 100%, 121%, 70% and 53% for the standard, Mn-Met, MnO (a) and MnO (b), respectively [225]. A later study in reagent-grade Mn sources assessed MnSO_4_.H_2_O as the standard and MnO, MnO_2_ and MnCO_3_. Based on multiple linear regression slopes for liver, kidney and bone Mn concentrations, RBV averaged 58%, 33% and 28% for MnO, MnO_2_ and MnCO_3_ respectively, compared with 100% for the MnSO_4_ standard [226]. Variations between the studies gave RBV ranging from a low of 20% with kidney Mn as the response criterion, to 164% using an organic source with bone Mn as the parameter of choice. All studies used the slope ratio method for calculation of relative bioavailability values but added levels of mineral in the studies, as well as choice of response criteria, varied (Table 6 and Table S10).

##### Zinc Relative Bioavailability—Sheep

In one of the earliest mentioned studies examining the effects of dietary chelated and sequestered Zn and Zn sulphate on growing lambs fed a purified diet, signs of Zn deficiency were seen in those fed the inorganic source whereas no signs of deficiency were observed with those fed the chelated Zn. RBV ranged from 91% to 125% for chelated Zn and 97% to 108% for sequestered Zn compared to the sulphate standard. The highest values were obtained using growth rather than plasma Zn as a response criterion [227].

Studies have also demonstrated that organic Zn sources are metabolised differently than inorganic sources. Spears and Samsell reported that Zn retention of lambs fed Zn-Met was greater than for lambs fed diets supplemented with ZnO [228]. The authors further suggested that Zn was metabolised differently after absorption, and that the attachment of Zn to methionine may alter the mode of absorption and transport of Zn in the body compared to Zn from ZnO. In a later study, Spears also reported that in lambs fed Zn methionine or ZnO, Zn absorption was similar for both treatments, but Zn retention and blood Zn levels were greater in the labs fed the organic form, indicating it may have been metabolised differently [177]. The RBV for Zn-Met in the study were determined using ZnO as the standard and ranged from 95% to 103% (Table S16). More recent studies comparing Zn-Met to reagent grade Zn sulphate found much higher RBV using the organic form in a range of response criteria [154,221]. In one such study, relative bioavailability values of Cu and Zn from Cu-Met and Zn-Met were found to be 33% and 52% higher than from inorganic Cu and Zn sulphate, respectively, in ewes [221]. Another study in ewes supplemented with 40% less Zn from chelated sources (Zn-Met) showed that milk yield increased by 12%. Increases of 26% and 31% in protein and fat production were also observed in comparison with inorganic Zn sulphate [229].

Rojas et al. [230] reported that sheep fed Zn-Lys had higher MT concentrations in the liver, kidney and pancreas although mean MT concentrations in sheep fed Zn from Zn oxide, sulphate or methionine were not different from the controls. Zinc-Lysine increased liver Zn concentrations 206% compared to the non-Zn supplemented controls, while Zn sulphate and methionine increased liver Zn by 55%. A calculated RBV for this study gave a value of 114% for Zn-Lys using reagent grade Zn sulphate as the standard [135].

Recent work assessed several OTM including a bis-glycinate chelate and a proteinate against the sulphate form. Similar bioavailability between organic sources was found, although the authors noted there may be differences between sources in their effects on mineral and antioxidant status. Furthermore, several different response criteria were assessed and RBV varied between them. The proteinate yielded higher values than either the bis-glycinate chelate or sulphate when assessed in kidney. The bis-glycinate chelate produced higher values when plasma and liver Cu/Zn SOD were considered and the sulphate yielded the highest values in the liver [231].

#### 3.3.3. Poultry

Organic trace minerals have been used in poultry feeds with positive effects on body weight gain, feed conversion efficiency, immune status and eggshell quality [232,233,234,235]. A 2014 review noted that the majority of findings from evaluated studies indicated that OTM are an effective source of microelements and can replace inorganic forms of minerals in poultry diets, often at lower inclusion levels resulting in a reduction in poultry excreta mineral content which has positive environmental benefits [236]. A 2020 review of broilers under *Eimeria* challenge noted a beneficial effect using a proteinate form of trace minerals and also stated the importance of maximising the absorption of trace minerals when intestinal function may be compromised [237]. From the published RBV data in the Supplementary Materials (Tables S2, S6, S9 and S13–S15), it is clear that the majority of studies selected poultry as the species of choice to evaluate the biological availability of trace minerals.

##### Copper Relative Bioavailability—Poultry

In the last decade, many studies have shown positive results using organic forms of Cu in poultry diets [170,238,239,240,241,242,243]. For studies with calculated RBV using the slope ratio method and with Cu sulphate as the standard, the organic forms showed higher relative bioavailability in most cases (Table S2). A distinct outlier of 188% for cupric acetate was calculated using the triple-point method rather than the slope ratio method which may provide reasoning for such an unexpectedly high value [244].

Older work from Aoyagi and Baker [245] concluded the standard, cupric sulphate, cupric basic carbonate, cuprous oxide, Cu-Met and Cu-Lys were all used similarly, whereas cuprous chloride was more available and cupric oxide was unavailable. Later work by Guo et al. [155] reported a RBV difference of 13 percentage points between two experiments for Cu-Lys, which they proposed was due to different breeds of chicks used in the two different trials.

Liu et al. [246] evaluated the bioavailability of organic Cu proteinate relative to inorganic Cu sulphate in broilers fed a conventional corn-soybean meal diet. Based on slope ratios from multiple linear regressions of log_10_ liver and bile Cu concentrations with daily Cu intake, the estimated relative bioavailability of Cu proteinate was 78.8% and 79.3%, respectively, as compared to inorganic Cu sulphate (100%); however, these differences were not statistically significant [246]. Other RBV for Cu proteinates in poultry produced values in the range 99–111% indicating the proteinate form is at least as bioavailable as the sulphate standard. This particular study reported values of 122 ± 5.3% for a Cu-amino acid chelate and 111 ± 6% for a proteinate (Cu ProC) in one experimental trial. Another experimental trial in the same study with a different breed of bird reported values of 109 ± 8.4% for one proteinate (Cu ProA) and 105 ± 7.5% for a second proteinate (CuProB) [155].

##### Iron Relative Bioavailability—Poultry

A large volume of research has been carried out on Fe bioavailability values in poultry dating back as early as the 1930s for ITM [247,248]. All published studies up to 1996 used an Fe sulphate form as the standard and haemoglobin regeneration (Hb reg) as the response criterion of choice. Newer studies began to look at both OTM and ITM and alternative response parameters such as liver Fe, haematocrit and SDH mRNA in liver and kidney [249,250,251]. Briefly, looking at Table S6 for the newer studies with OTM, the Fe proteinate “M” used in the Zhang et al. study is the same Fe proteinate as that used in the Ma et al. study but it is bioavailability value relative to FeSO_4_.7H_2_O is about 40% lower in the Ma et al. study (116% vs. 154%). The authors suggested the difference may be due to the reduced feed intake inhibiting growth and the Fe use of broilers fed a purified casein-dextrose diet in the Ma et al. study [250,251].

##### Manganese Relative Bioavailability—Poultry

The majority of experiments regarding Mn bioavailability have used chicks (Table 6). Reviewing the inorganic data compiled for poultry in Table S9, it can be noted that Mn chloride was nearly equal to the sulphate standard in multiple studies [252,253,254]. Manganese oxide values ranged from 46% to 103% although the majority of values are less available than the Mn sulphate standard [255,256,257,258,259,260,261,262]. Those that were higher used a feed-grade version of the standard to calculate RBV [263]. Manganese carbonate and Mn dioxide are also used less effectively in most reported cases [253,255,264], although some very early studies reported values similar to the sulphate standard [252,254,258].

For newer work on OTM, several studies on methionine and proteinates were assessed and their respective RBV were found to be higher than the inorganic standards using a range of criteria [261,265,266,267,268,269,270]. One study by Wang et al. [271] had lower values for proteinates (86%, 95% and 103% in heart, MnSOD and MnSOD mRNA respectively), but response criteria differed to most other studies making direct comparisons difficult. The authors also stated that bioavailability between the Mn proteinate and Mn sulphate was not significantly different (*p* > 0.21). An interesting facet of this particular study was the discussion on chelation strength of the proteinate. The authors found bioavailabilities of organic Mn sources for broilers were closely related to their chelation strengths. The proteinate in this particular study was considered to have “weak” chelation strength, and therefore was more easily dissociated in the GI tract and absorbed as ions similar to its inorganic counterparts, so bioavailability was similar [271]. Several other similar studies have shown organic sources with optimal chelation strengths were more bioavailable than inorganic sources or weakly bound organic sources [155,178,246,250,251,271,272,273,274,275,276,277,278]. This important concept is discussed in further detail in Section 3.4.

Studies on other OTM such as amino acid complexes and a chelate of HMTBa also reported higher RBV than the sulphate standard [178,257,277,279]. The only amino acid values with RBV lower than the sulphate standard were found in a 2003 study by Miles et al. This study examined an amino acid chelate based on a product derived from strong base hydrolysis and neutralisation of lipoproteins, with the source of lipoproteins being fractured cell walls of microbes generated from biological syntheses. Fatty acids were present in the final product, originating from the lipoprotein substrate according to the authors. Although they concluded the relative bioavailability of Mn in the Mn-AA chelate was found to be approximately 15% lower than the reagent-grade sulphate when all data were included in the regression modes, the bioavailability was found to be equal when the greatest level of supplementation was eliminated [280]. Recent work by our research group has shown the difference in stability between products is highly dependent on their respective production processes which may also explain the RBV for this particular product [59].

As discussed in Section 2.3, the potential of OTM to maintain their structural integrity in the digestive tract, arrive at absorptive sites in the small intestine as the original intact molecule and then be absorbed and metabolised in several possible ways is supported by several articles [155,266,281]. Increased resistance to dietary antagonists is a primary contributor to this and a study by Fly et al. supported this by showing the bioavailability of Mn from Mn-Met and MnO varied greatly in chicks fed with a purified diet compared with estimates from chicks fed a mixture of purified diet and a maize–soybean meal diet. Results showed Mn from Mn-Met was 30% more bioavailable than Mn from MnO in the purified diet but was 74% more bioavailable in the mixture [266]. A 2005 study from Li et al. also showed higher RBV for the organic products in a high Ca diet, implying that organic Mn sources with moderate or strong chelation strength offer partial or complete resistance to interference from high dietary calcium during digestion [277]. Similar results have also been found for other minerals. Huang et al. found an increase (11% to 22%) in Zn proteinate RBV in diets containing higher phytate levels compared to those under a low phytate diet for broilers [282]. Ward et al. compared carbonate and sulphate forms of Cu to a Cu proteinate product and found equal RBV but in the presence of added molybdenum, a higher use of Cu appeared to occur with the proteinate form [175]. Richards et al. also came to similar conclusions with Zn chelated to HMTBa using elevated calcium and phosphorous as the antagonistic factors although exceptionally high RBV were obtained when compared to other studies which may have been due to lack of control treatments in one of the experiments as outlined by the authors [163].

Other work supported the inclusion of OTM under heat stress conditions which magnifies the difference in bioavailability between products. Smith et al. highlighted an increase in the RBV of proteinates compared to MnO and MnSO_4_ under heat stress conditions [261]. One result from that study found that in 48-d-old birds reared under thermoneutral conditions, the corresponding RBV of Mn from proteinate and oxide sources were 125% and 83%, respectively. However, for birds under heat distress, the RBV for the proteinate increased to 154%, whereas the oxide remained practically unchanged at 82%.

##### Zinc Relative Bioavailability—Poultry

Zinc bioavailability studies in poultry encompass chickens, turkeys, and quail (Tables S13–S15). One standout result from the multi species table for Zn (Table 7) is the excessively wide range of RBV for Zn oxide (22% to 108%), which emphasises how parameters such as origin, manufacturing source, trial set up and evaluation method can impact results. Highest ZnO values were from a 1960 study using the three-point method of calculation and used growth as the response criterion [283]. Another study with striking differences for ZnO is the Edwards and Baker work from 1999. Among the feed-grade sources assessed in this work, the process by which it was manufactured had an enormous effect on the RBV: hydrosulphide process (93% to 97%), French process (84%) and Waelz product (32% to 42%) based on weight gain [141].

Later work from the same authors showed RBV for Zn sulphate with different diet types including diets containing antagonists such as phytate [284]. Results indicated that the RBV of Zn in dehulled soybean meal (SBM) was 78% when the phytate containing soy protein concentrate (SPC) was used but was only 40% when the phytate free egg white diet was used. The authors concluded the phytate contained in the SPC basal diet reduced the efficiency of using the supplemental inorganic Zn from ZnSO_4_.7H_2_O [284]. Other antagonistic interactions including those between Zn and Cu can also affect performance in chicks. A study assessing the effects of feeding different forms of Zn and Cu on the performance and tissue mineral content of chicks found the antagonism between Zn and Cu occurred when inorganic forms of the two minerals were included in a chick diet but not when OTM were used [285].

The Zinc–Methionine results noted in Table 7 and Table S13 also display significant RBV variance (77% to 292%). Wedekind et al. [150], noted that diet type had a considerable effect on the relative bioavailability estimate. Relative to feed-grade ZnSO_4_.H_2_O, the RBV for Zn-Met in an amino acid diet, a soy-isolate diet and a practical corn-soybean diet was found to be 117%, 177% and 206% respectively. It was assumed that this was due to the amount of phytate and soluble fibre, which forms complexes with the Zn of inorganic origin [150]. Later studies by the same author in swine did not show similar results for Zn-Met, and in fact the RBV was below that of the sulphate, which may be due to species and dietary differences [148]. Dietary calcium concentrations have also been demonstrated to differentially affect Zn bioavailability from organic and inorganic sources of Zn. Bioavailability of Zn-Met was 166% relative to Zn sulphate at a dietary calcium concentration of 0.60% Ca and 292% at 0.74% Ca [286].

Other studies that showed how various response parameters affected RBV included a 2000 study by Cao et al. which noted bone was a more accurate measure than mucosal Zn which was noted to produce data with very large standard deviations [154]. Sandoval et al. highlighted the differences between reagent grade and feed-grade standards. The same paper also had RBV calculated using dietary intake as well as dietary concentrations but the fit to a linear model was poor compared to that calculated with dietary concentration as the independent variable [151].

From the tables, it is clear that many studies have found positive results, with higher bioavailability being conferred when using OTM. For example, RBV for proteinates in chick diets ranged from 70% to 200%, depending on the product, but most were above the reference standard value of 100% [147,278,282,287,288,289]. In some cases, even though there appears to be no difference in relative bioavailability values between the organic source and the inorganic standard, other parameters indicated otherwise. For example, a 2013 study, based on slope ratios from multiple linear regressions of tibia ash Zn concentration and pancreas MT mRNA level with daily analysed Zn intake, reported no significant (*p* > 0.05) difference in bioavailability between Zn proteinate and Zn sulphate for chicks. However, Zn from Zn proteinate was more effective than Zn sulphate in enhancing feed intake and tended to improve the growth rate of broilers [278].

#### 3.3.4. Swine

Studies confirming the positive effect of inclusion of OTM in pig diets include reports of higher mineral retention using organic minerals such as Cu-Lys in weanling pigs [290], Zn-amino acid chelate in growing pigs [161] and Cu and Zn proteinates in weanling pigs [291]. Pigs fed mineral-proteinates have also been shown to gain weight at a higher rate than pigs fed the sulphate form in the nursery phase [292]. A study on the effects of dietary organic and inorganic trace mineral levels on sow reproductive performances found increases in litter birth weights, live pigs per litter and an increase in the number of pigs per litter when OTM in the form of proteinates were used [293].

From an environmental perspective, it has been possible to reduce dietary inclusion levels of inorganic minerals by using lower levels of an organic form without affecting pig performance [172,294,295,296,297,298,299,300,301,302]. Some brief examples include a study by Veum et al., who replaced 15% to 36% of the inorganic minerals (Fe, Zn, Mn, Cu, Se and I) in a mineral premix with proteinate forms resulting in increased ADG and gain:feed in weanling pigs [303]. Increased feed intake and ADG was found in another study with diets containing reduced levels of minerals from organic sources versus inorganic sources [295]. A study which looked at the effect of replacing ITM at lower organic levels on growth performance, blood parameters, antioxidant status, immune indices, and faecal mineral excretion in weaned piglets found that replacing high doses of ITM with low concentrations (1/3) of OTM does not adversely affect the growth performance of piglets [300]. At low levels, total replacement of ITM with OTM improved IgG and reduced faecal excretion of Cu, Zn, Fe, and Mn, thereby mitigating environmental pollution [300].

##### Copper Relative Bioavailability—Swine

From the tables compiled (Table 4 and Table S4), Cu proteinate had the highest RBV for Cu. However, the study which produced the highest results used tribasic copper chloride (TBCC) as the standard in place of the usual sulphate standard. The study also observed that pigs fed Cu proteinate absorbed and retained more Cu and excreted less Cu than those fed TBCC when supplemented with 80 mg kg^−1^ and above. Copper from Cu proteinate was significantly more bioavailable to weanling pigs than TBCC in stimulating growth and enzyme activities, decreasing diarrhoea frequency and faecal Cu contents to the environment [304]. Other studies have also found positive benefits from chelation of dietary trace minerals including improved apparent total tract digestibility and retention of Cu in pigs by preventing formation of insoluble complexes along the GI tract [299,301,305,306,307].

##### Iron Relative Bioavailability—Swine

Newborn piglets are susceptible to Fe deficiency anaemia for several reasons, including rapid growth rate, confinement rearing and lack of placental or mammary Fe transfer from dam to offspring [308]. As such, an important industry focus has been to improve the Fe status of the newborn piglet through the use of more bioavailable Fe sources. In pigs, ferric citrate and ferric choline citrate were found to be essentially equal in their bioavailability to the sulphate sources [308,309]. However, the results noted in Table 5 and Table S8 illustrate that quite high RBV for these mineral sources have been reported. Examining the parameters closely, it can be observed that the response criteria and the added level (mg kg^−1^) are quite high in the selected studies and the method used to calculate the RBV also differed which may explain the variance.

Ferrous carbonate varies in bioavailability depending on the source but overall is less bioavailable than the sulphate standard. The variation in ferrous carbonate values in Table 5 is a good example of how data from one experiment can provide bioavailability estimates which vary widely depending on the calculations used. Ferric oxide is almost completely unavailable [310]. Organic sources including Fe proteinate and Fe-Met were generally higher than the inorganic sources although the methionine values varied greatly between studies (68% to 183%) [311,312,313]. Iron chelated to amino acids has been reported to lead to increased transfer of Fe across the placenta and into the foetus [314]. Similar effects have been reported for piglets from sows whose diets were supplemented with Fe proteinate 21 days pre-farrowing, in which significantly higher erythrocyte counts and haemoglobin levels were noted than in piglets from sows receiving inorganic Fe sources. In addition, liver Fe levels were notably higher in piglets from the proteinate supplemented group [315].

##### Manganese Relative Bioavailability—Swine

Manganese deficiency is a greater issue in poultry than in swine to which the sheer volume of studies in avian models compared to swine can attest. Studies with published RBV are limited with only carbonate and oxide values available [316]. However, there are published articles demonstrating the effect of dietary supplementation with organic Mn forms on parameters such as hoof lesions, reproductive performance, mineral status and faecal mineral excretion [172,300,301,317].

##### Zinc Relative Bioavailability—Swine

With concerns about heavy metal accumulation in the environment and the potential for antimicrobial resistance, the maximum level of Zn permitted in animal feed will reduce to 150 mg kg^−1^ in the EU in June 2022. Using high levels of Zn oxide will no longer be permitted. Post-weaning diarrhoea (PWD) due to *Escherichia coli* has a significant economic impact on pig production and is one of the main reasons pharmacological levels of ZnO were used. Recent reviews on trace mineral supplementation and its effect on the intestinal health of monogastrics have highlighted the importance of precise supplementation to maintain nutritional benefits but minimise environmental impact [35,318]. Organic forms of Zn can be used as part of a nutritional solution improving gut integrity/improved growth and performance thereby reducing the need for the oxide form.

From a bioavailability perspective, Miller et al. reported that the bioavailability of Zn in Zn dust was higher (30%) for pigs relative to analytical grade ZnO [319]. Feed-grade ZnO for pigs was found to have a bioavailability of only 56% to 68% relative to that in a feed-grade sulphate [320]. A 1994 study by Wedekind et al. found estimates of Zn bioavailability differed depending on which variable (metacarpal, coccygeal vertebrae, or plasma Zn concentrations) was used. Overall trends in the study indicated the following rankings: ZnSO_4_.H_2_O > Zn-Met > ZnO > Zn-Lys [148]. Other studies using Zn-Lys showed higher bioavailability values (92% to 110%) but response criteria and added levels varied between studies [149,320,321]. A Zn-amino acid chelate study showed similar bioavailability to the sulphate standard [322]. Studies on tetrabasic zinc chloride (TBZC) reported high RBV, but were conducted using ZnO as the standard, and as such are not directly comparable with other results using the sulphate standard [323].

### 3.4. Mineral Stability and Associated Relationship with Bioavailability

As outlined in Table 3, many factors affect bioavailability. One area of interest which has generated several studies, relates to the chemical form of the mineral. Organic trace minerals are distinct from ITM due to their mineral bonding ability. The process of complexing or chelating elements such as Cu, Fe or Zn, for instance, typically involves reacting inorganic mineral salts with a suitable bonding group, such as a peptide or amino acid, after which the mineral becomes part of a biologically stable structure. Stability constants or formation constants are commonly used to provide an indication of the strength of interaction between a metal and the ligand in a chelate or complex [324,325]. In general, the higher the stability constant’s value, the greater the chelation strength and thus the relative proportion of bound mineral to free mineral is higher under a given set of conditions although there are exceptions to this.

Previous work has shown that the type of ligand influences the stability of a given complex or chelate but the position of the amino acids in a peptide sequence also has a significant impact [59,326,327,328]. This again serves to emphasise why grouping so many different products under the broad “OTM” term is inaccurate as products such as amino acid complexes, amino acid chelates, polysaccharide complexes and proteinates have been shown to have different mineral binding properties and different pH stabilities [59].

With such differences in stability between products, it is highly unlikely they will be absorbed or used in the same way or increase the bioavailability of a given mineral to the same extent. Given that absorption does not take into account the potential use of a mineral in the body, the terms absorption and bioavailability are not the same; however, they often match closely [147,178,275,277,329].

The formation quotient or Q_f_ value is a quantitative measure of chelation or complex strength between metals and ligands and is determined using polarography by measuring the shift in halfwave potential ΔE_1/2_ [330]. Several studies in poultry have determined RBV in addition to calculating Q_f_ values for a range of OTM products to determine the level of correlation [147,154,155,178,246,250,251,271,277,278,282]. In general, chelates with a Q_f_ value below 10 are considered weakly chelated; moderately strong chelation values are in the range of 10 to 100, and strongly chelated values are those above 100 [154]. Results have indicated bioavailability values were more closely related to chelation strength as measured by polarography than to chemical traits assessed by solubility or structural integrity [147,178]. Sources with moderate and high chelation strengths had the highest relative bioavailability, whereas those with weak chelation strengths were found to be only as available as their inorganic sulphate forms at best [272,273,274,275,331]. One hypothesis is that OTM with optimal chelation strength could prevent dissociation in the digestive tract and reach the intestinal brush border more efficiently resulting in a higher bioavailability. Weak chelates are likely to dissociate in a similar fashion to the inorganic forms and those with exceptionally strong chelate strengths may not release the mineral at all rendering it unavailable [250]. Those sources with exceptionally strong chelation strength were significantly less available than even the inorganic sulphate for all studies except for those in Fe [251,332]. Reasons for the noted discrepancy in the Fe studies include the fact that divalent Fe is very active and easily oxidised to ferric Fe, which is less available to animals, and organic Fe with stronger chelation strengths could better avoid this oxidation reaction and, therefore, be more available to the animal [251]. In a second study, the authors suggested that organic Fe with greater Q_f_ values could better resist the chelating effect of interference factors such as dietary calcium, rendering higher Fe absorption [332].

Based on the previous studies conclusions of strong correlation between bioavailability and chelation strength or stability, several additional studies in poultry can be found in the literature that report values for absorption and chelation strength but without published RBV. These values in Table 8 provide an indication of the bioavailability of a selection of products based on Q_f_ values [272,273,274,275,331,333,334].

Some of the values published in Table 8 (Ji et al. [273,274], Luo et al. [334]) are based on the same Mn products (MnMet E, Mn AA B, and Mn AA C) analysed in two previous studies by Li et al., which are contained in Supplementary Table S9. These products have published RBV in the earlier studies for Mn in bone, heart, MnSOD activity in heart and MnSOD mRNA in heart under both normal dietary conditions and high dietary Ca inclusion [178,277]. The RBV ranged from 93% to 133% for the 3 products under standard dietary conditions and 102% to 148% under dietary conditions with high levels of calcium as an antagonistic factor. These values are lower than those reported in Table 8 based on absorption; however, large differences were noted in the values depending on the criteria used, e.g., duodenum, ileum or jejunum. Overall, the trend observed in relation to OTM having better bioavailability than ITM is confirmed in the tables with published RBV.

## 4. Replacement of ITM with OTM in Feedstuffs

Many of the minerals consumed by production animals appear in faeces, particularly if pharmacological or “growth-promoting” levels are applied. This can result in substantial excretion of minerals in slurry and manure that pollute drainage water or affect plant physiology giving rise to long-term environmental concern [335,336].

Taking Cu as an example, the panel on Additive and Products or Substances used in Animal Feed (FEEDAP) reviewed the Cu requirements of production animals and recommended a reduction in some of the currently authorised maximum contents (CAMC) for total Cu in feed. In the case of swine, reduction from 170 mg to 25 mg kg^−1^ feed in piglets would have the capacity to save 1200 tonnes of Cu/year being spread in the field, and thus reduce total Cu emissions from farm animal production by about 20% [14].

One important strategy for reducing trace mineral concentration in diets to mitigate the environmental issue, without affecting animal performance, is inclusion of organic sources of the minerals which have greater bioavailability. Over the years, research has progressed from using solely OTM to partially replacing ITM with OTM at lower levels to total replacement of ITM with OTM with no negative effect on performance parameters [56,165,176,180,233,294,296,301,302,303,337,338,339,340,341,342,343,344,345].

### 4.1. Ruminants

In beef, a study published in 2016 used OTM at 60% of commercial levels for ITM and found improved health and performance, a reduction in mortality of 57% and reduction in mortality due to bovine respiratory disease (BRD) of 69% [346]. Another study in beef confirmed that OTM have a strong impact on animals’ metabolism and immune functions, that result in an improvement in growth performance, health and antioxidant status together with carcass and meat quality. The authors also reported significant reduction in the incidence of BRD when using OTM in place of ITM [186]. With increases in antioxidant activity, extended shelf life is feasible, and meat also showed increased water retention, improving profitability and texture.

A recent study in dairy compared the effect of complete replacement of certain ITM with organic, chelated minerals and found cows in the organic group had higher milk yield, protein synthesis and milk lactose content. Pregnancy rate also improved, and mastitis rates were lower for the OTM group. Somatic cell counts were also consistently lower in the cows fed OTM [347]. In other research, dairy scientists at Pennsylvania State University studied a herd over three generations and found that heifer calves fed OTM had improved overall health as measured by general health scores and plasma haptoglobin. This was true even for heifer calves who did not consume OTM themselves, indicating that the effect was due to maternal nutrition, but the effect is enhanced when both cow and heifer are fed OTM [348]. A second study by the group found heifers fed OTM tended to calve earlier (22 d) than those supplemented with ITM and overall milk yield was greater in OTM supplemented heifers. [189]. Reaching reproductive maturity almost 1 month sooner provides savings in management costs in addition to a reduction in carbon footprint [349]. Uchida et al. [338], replaced a portion of an inorganic mineral supplement with organic sources (Zn, Mn, Cu, and Co) in a dairy cow diet fed from calving until first breeding service. Cows fed the diet containing the OTM had fewer days to conception and tended to have fewer days to first service and fewer services per conception. In a similarly designed study, Ballantine et al. also replaced a portion of an inorganic mineral supplement with organic sources (Zn, Mn, Cu, and Co) but the diet was fed from 21 days prepartum until 250 days postpartum. In cows pregnant at 250 days in milk, those fed organic sources produced more milk, had fewer days open (147 vs. 169 days), and tended to have a higher first service conception rate (27% vs. 18%) [339].

Positive results using OTM in ewes have also been reported with lower mineral excretion observed, indicating better use. Improved feed conversion ratio, higher gut absorption and increased plasma and liver concentrations of Cu and Zn also supported the hypothesis of better bioavailability compared to the sulphate standard [221].

### 4.2. Poultry

Necrotic enteritis has enormous economic impact in the poultry industry, approaching USD 6 billion annually with mortality rates of 1%/d [350,351]. Studies have shown that poultry receiving OTM show significant positive changes to the expression of genes related to improved disease resistance [352]. In recent years, there has also been a considerable reduction in the recommended levels of organically complexed minerals in broiler chicken diets without any negative effects on their performance [56,176,353,354], antioxidant defence systems [355], haematological and biochemical parameters [356], and meat quality parameters [356]. In one study, broilers were fed OTM at levels 75% lower than commercial levels for ITM. These birds reached a desirable market weight in the standard 6-week period and showed no signs of deficiency or disease, even though they were fed only one-quarter the mineral ration of their inorganic-fed counterparts [285]. Another study in chicks stated OTM diets had a positive effect on economic efficiency and concluded that replacing ITM with OTM improved birds performance and enhanced immune response of chicks [344]. Leeson et al. showed that using OTM with greater bioavailability did not affect body weight gain and had little effect on feed efficiency of broilers even when fed at 20% of the ITM level, and at the same time reduced the environmental contamination due to lower excretion of minerals [357]. A 2011 study by Ao et al. found that replacing ITM with OTM (peptide-chelate at the rate of 50% or 100%) improved performance and enhanced immune response of chicks [342].

Based on the aforementioned studies, it is clear that many OTM can be supplied to broiler diets at much lower levels than the current recommendations for ITM without negatively impacting broiler performance and resulting in a positive environmental effect due to decreased excretion of excess mineral. However, it is important again to highlight that not all OTM are equal in terms of bioavailability and effectiveness. For example, a 2011 study reported positive benefits from substituting 100% or 80% of a particular type of OTM (Zn and Mn HMTBa) in place of 100% ITM but greater substitution levels in the region of 60% OTM significantly decreased average daily gain (*p* < 0.01), length of metatarsus, serum ALP activities and mRNA expression of Zn transfer proteins and also increased mortality and culling rate (*p* < 0.05) [358].

### 4.3. Swine

Veum et al. [303] replaced 15–36% of supplemental inorganic Zn, Fe, Cu and Mn with chelated metal proteinates and observed increased gain and feed conversion in nursery pigs compared with those fed only ITM. Total replacement with OTM in swine improved average daily gain, increased slaughter weight by 2 kg and improved slaughter efficiency which generated cost savings. Additional studies found that OTM had the same effect in pork loin and chicken breast improving the producer’s bottom line [359,360]. Zhang et al. [300] looked at the effect of replacing ITM with lower levels of OTM on growth performance, blood parameters, antioxidant status, immune indexes and faecal mineral excretion in weaned piglets and found that replacing high doses of ITM with low concentrations (1/3) of OTM does not adversely affect the growth performance of piglets. At low levels, total replacement of ITM with OTM improved IgG and reduced faecal excretion of Cu, Zn, Fe, and Mn, thereby mitigating environmental pollution [300]. Pierce et al. replaced all four minerals (Cu, Fe, Zn and Mn) with only 25% of the NRC requirement in the form of proteinate (no inorganic salts were fed). The results showed a 34% reduction in mineral excretion without compromising weight gain in broilers [298].

## 5. Conclusions

This review concludes that OTM have better bioavailability than ITM, with new data continuously proving better absorption and use in production animals. Even when some papers report no differences in relative bioavailability values between the inorganic and organic forms based on selected criteria, on closer inspection, results often show the organic form has advantages when additional parameters are assessed, in the presence of dietary antagonists or under stress conditions. Studies have also unequivocally shown the advantages of incorporating organic forms from an environmental and economic perspective. OTM have been perceived as being more expensive based on direct comparisons of cost versus ITM. However, the return on investment in numerous studies has emphatically shown the advantages of incorporating organic forms of minerals. The tables contained in the review provide a useful resource for those in the industry looking to compare and contrast products and the information required to make an informed decision when considering trace mineral sources in dietary formulations.

## Figures and Tables

**Table 1:** Primary functions and inclusion levels of selected trace minerals and signs of deficiency.

Mineral	Function	Signs of Deficiency	EU Maximum Inclusion Levels: Maximum Content of Element in mg kg^−1^ of Complete Feed with a Moisture Content of 12%
Copper	Involved in metabolic reactions including cellular respiration, tissue pigmentation, haemoglobin formation (caeruloplasmin) and connective tissue development [9,10]. Essential component of several metalloenzymes [11,12]. Protects against oxidative stress [12,13].	Muscle weakness, iron-deficient anaemia, hypopigmentation, bone changes resembling scurvy, defective connective tissue synthesis, hair abnormalities, impaired myelinisation of nerve tissues and neurological defects, altered lipid metabolism and cardiac malfunction [14,15,16].	Bovines: Bovines before the start of rumination: 15 (total), Other bovines: 30 (total) Ovines: 15 (total) Caprines: 35 (total) Piglets: Suckling and weaned up to 4 weeks after weaning: 150 (total), from 5th week after weaning up to 8 weeks after weaning: 100 (total) Crustaceans: 50 (total) Other animals: 25 (total) [17]
Iron	Important for physiological function—haemoglobin, in which the heme portion functions to carry oxygen from the lungs to the tissues, mitochondrial Fe enzymes essential for oxidative production of cellular energy through Krebs cycle, transport of oxygen by myoglobin to cells and tissue of muscle. Important for immune function and lipid metabolism.	Supressed growth and blood volume [18]. Decreased animal performance, loss of appetite and weight, spasmodic breathing and ultimately death [19].	Ovine: 500 (total (^1^)), Bovines and poultry: 450 (total (^1^)) Piglets up to 1 week before weaning: 250 mg/day (total (^1^)) Pet animals: 600 (total (^1^)) Other species: 750 (total (^1^)) [20]
Manganese	Constituent of multiple enzymes. Component of the organic matrix of bone and is essential for cartilage development. Involved in the metabolism of calcium and carbohydrates. Necessary for the utilisation of biotin, vitamin B1 and vitamin C [21]. Metabolic association between manganese and choline which affects fat metabolism in the liver [22].	Impaired growth, skeletal abnormalities, abnormal reproduction function, ataxia in newborns, impaired carbohydrate and lipid metabolism and impaired mucopolysaccharide synthesis [23]. Poultry specific issues include: Perosis (slipped tendon), thin eggshell quality, chondrodystrophy in embryonic chicks, reduced egg production and hatchability	Fish: 100 (total) Other species: 150 (total) [24]
Zinc	Activates several enzymes. Component of many important metalloenzymes. Critically involved in cell replication and in the development of cartilage and bone [25]. Involved in protein synthesis, carbohydrate metabolism and many other biochemical reactions [26,27].	Retarded growth, decreased feed intake, abnormal skeletal formation, alopecia, dermatitis, abnormal wool/hair/feather growth and impaired reproduction. Fetal abnormalities. Reduced egg hatchability [25]. Parakeratosis, diarrhoea and thymic atrophy [28].	Dogs and cats: 200 (total) Salmonids and milk replacers for calves: 180 (total) Piglets, sows, rabbits and all fish other than salmonids: 150 (total) Other species and categories: 120 (total) [29].

^1^ The amount of inert iron is not to be taken into consideration for the calculation of the total iron content of the feed.

**Table 2:** Organic trace mineral definitions comparing AAFCO and EU definitions.

	AAFCO		EU
Metal Proteinate (57.23)	The product resulting from the chelation of a soluble salt with amino acids and/or partially hydrolysed protein. It must be declared as an ingredient as the specific metal proteinate, e.g., copper proteinate, zinc proteinate etc.	Metal chelate of protein hydrolysates	A powder with a minimum content of x% metal where x = 10% copper, iron, manganese and zinc. Minimum of 50% copper, iron, manganese and 85% zinc chelated. Chemical formula: M(x)_1__–__3_. nH_2_O, M = metal, x = anion of protein hydrolysates containing any amino acid from soya protein hydrolysate.
Metal Polysaccharide Complex (57.29)	The product resulting from complexing of a soluble salt with a polysaccharide solution declared as an ingredient as the specific metal complex, e.g., copper polysaccharide complex, zinc polysaccharide complex etc.		
Metal Amino Acid Chelate (57.142)	The product resulting from the reaction of a metal ion from a soluble metal salt with amino acids with a mole ratio of 1 mole of metal to 1 to 3 (preferably 2) moles of amino acids to form coordinate covalent bonds. The average weight of the hydrolysed amino acids must be approximately 150 and the resulting molecular weight of the chelate must not exceed 800. The minimum metal content must be declared. When used as a commercial feed ingredient, it must be declared as a specific metal amino acid chelate, e.g., copper amino acid chelate, zinc amino acid chelate etc.	Metal chelate of amino acids hydrate	Metal amino acid complex where the metal and the amino acids derived from soya protein are chelated via coordinate covalent bonds, as a powder with a minimum content of 10% copper and zinc, 9% iron and 8% manganese. Chemical formula: M(x)_1__–__3_. nH_2_O, M = metal, x = anion of any amino acid from soya protein hydrolysate. Maximum of 10% of the molecules exceeding 1500 Da.
Metal Amino Acid Complex (57:150)	The product resulting from complexing a soluble metal salt with an amino acid(s). Mineral metal content must be declared. When used as a commercial feed ingredient, it must be declared as a specific metal amino acid complex, e.g., copper amino acid complex, zinc amino acid complex etc.		
Metal (specific amino acid) complex (57.151)	The product resulting from complexing a soluble metal salt with a specific amino acid. Minimum metal content must be declared. When used as a commercial feed ingredient, it must be declared as a specific metal, specific amino acid complex, e.g., copper lysine, zinc methionine etc.	Metal chelate of glycine hydrate (liquid)Metal chelate of glycine hydrate (solid)	A liquid with a minimum content of 6% copper or 7% zinc.Chemical formula: M(x)_1__–__3_. nH_2_O, M = Cu or Zn, x = anion of glycine A powder with a minimum content of 15% copper, iron, zinc and manganese and a maximum of 13% moisture for copper and 10% moisture for iron, zinc and manganese.Chemical formula: M(x)_1__–__3_. nH_2_O, M = metal, x = anion of glycine

**Table 3:** Factors affecting bioavailability in production animals.

Factor	Sub-Factor
Animal	Age Breed Health status Monogastric or ruminant Physiological state (e.g., growth, bone development, pregnancy, lactation, disease) Previous nutrition Production (performance) level and type of production Sex Species
Chemical aspects	Bond strength Chemical form and purity of the mineral sources Differences in dissociation rates of the mineral form from the ligand Particle size of the mineral Processing conditions/manufacturing method Solubility Stability
Dietary	Chemical composition of the diet (proximate analysis and mineral contents) Feedstuff composition of the diet and presence of dietary antagonists Level of supplementation of the minerals tested Overall diet digestibility Presence of antimicrobial growth promoters or (organic) acids Vitamin content
Environmental	Environmental stress Feeding method (dry or wet feeding; soaking) Housing and equipment Level of feeding expressed as energy level times maintenance requirement for energy Level of mineral intake Water supply level and quality
Evaluation	Reference/Standard source Model used for evaluation (dose-response; linear or non-liner) Choice of response criteria Direct or indirect measurement Duration of preliminary and test period Experimental design Levels of supplementation Number of replicates

**Table 4:** Multi-species table of relative bioavailability values (%) for copper ^1,8^.

Source	Cattle	Poultry	Sheep	Swine
Cupric sulphate	100	100	100	100
Copper acetate			100	
Copper amino acid complex/chelate		96–128	100	
Copper carbonate	86		97	
Copper chelate of HMTBa		111–112		
Copper chloride	98		96	
Copper chloride, basic	102–112			
Copper citrate	101			74–99
Copper EDTA	91–104		96	
Copper glycine/glycinate	131–157 ^2^		96	
Copper lysine	89–153 ^3^	92–124	68–97	73–101
Copper methionine		88–117	150–152	100–107
Copper oxide			81	
Copper proteinate	82–147	79–111	103	114–263 ^6^
Cupric acetate		93–188 ^2^	93 ^5^	
Cupric carbonate, basic		113		
Cupric carbonate		54–68 ^4^	121 ^5^	62–111
Cupric chloride	102–121	106–110	102–123	
Cupric chloride, tribasic (TBCC)	87–196 ^2^	70–134		97
Cupric oxide	0–64	0–69	22–48 ^5^	0–104 ^7^
Cupric sulphide	25		11–35	0–69
Cuprous acetate	100		98–110	
Cuprous chloride		81–145		
Cuprous iodide		46–82		
Cuprous oxide		92–98		

HMTBa = 2-hydroxy-4-(methylthio)butanoate; EDTA = ethylenediaminetetraacetic acid; TBCC = dicopper chloride trihydroxide (or tribasic copper chloride). ^1^ Complete range of values and expressed relative to response obtained with cupric sulphate except where noted. ^2^ Feed-grade sulphate used to obtain relative values. ^3^ A large SD was observed for copper lysine due to the important difference in the apparent absorption efficiency compared to the reference source [166]. Without this observation the RBV of copper lysine becomes 98 ± 5.3%. ^4^ Cupric acetate was used as relative standard in place of the sulphate form. ^5^ Cupric chloride was used as relative standard in place of the sulphate form. ^6^ TBCC was used as relative standard in place of the sulphate form. ^7^ Unexpectedly high value due to results by Buescher et al. showing cupric oxide had the same bioavailability as cupric sulphate using labelled Cu, which gave a large standard deviation (74 ± 21%). If this observation was omitted, the highest RBV of Cu in CuO for swine would have been 75%. ^8^ Extended details on each source including response criterion, method of calculation, diet type, added level (mg kg^−1^) and original source references are available in the supplementary data tables.

**Table 5:** Multi-species table of relative bioavailability values (%) for iron ^1,3^.

Source	Cattle	Poultry	Sheep	Swine
Ferrous sulphate heptahydrate	100	100	100	100
Ferric ammonium citrate		98–115		102
Ferric chloride		26–78		
Ferric choline citrate		102		118–144
Ferric citrate	107	70–76		89–192
Ferric EDTA	93			
Ferric glycerophosphate		86–100		
Ferric orthophosphate		4–36		
Ferric oxide		0–82		12
Ferric phytate	47			
Ferric polyphosphate				84–91
Ferric pyrophosphate		45		
Ferric sulphate		37–104		
Ferrous ammonium sulphate		99–100		
Ferrous carbonate–low ^2^	0–25	0–10	0–29	8–45
Ferrous carbonate–high ^2^	79	55–88	13–112	55–101
Ferrous chloride		98–106		
Ferrous EDTA		97–100		90–91
Ferrous fumarate		71–133		
Ferrous gluconate		97		
Ferrous sulphate, anhydrous		65–100		
Ferrous sulphate monohydrate		91–103		87–101
Ferrous tartrate		70–83		
Iron methionine		86–129		68–183
Fe-ZnSO_4_.H_2_O		112–126		
Iron proteinate		96–174		123
Iron, reduced		8–66		27–86
Sodium iron pyrophosphate		2–30		29–81
Zn-FeSO_4_.H_2_O		93–96		

EDTA = ethylenediaminetetraacetic acid. ^1^ Complete range of values and expressed relative to response obtained with ferrous sulphate heptahydrate. ^2^ Most ferrous carbonates have been reported to be low in Fe bioavailability; however, several were found to be of high availability and they are listed separately. ^3^ Extended details on each source including response criterion, method of calculation, diet type, added level (mg kg^−1^) and original source references are available in the supplementary data tables.

**Table 6:** Multi-species table of relative bioavailability values (%) for manganese ^1,3^.

Source	Cattle	Poultry	Sheep	Swine
Manganese sulphate		100	100	100
Manganese amino acid complex/chelate		84–148		
Manganese carbonate		32–101	20–93	95
Manganese chelate of HMTBa		116–154 ^2^		
Manganese dioxide		29–106	25–67	
Manganese methionine		95–174 ^2^	93–164	
Manganese oxide		46–103	31–91	96
Manganese propionate		139		
Manganese proteinate		86–163		
Manganous chloride		93–102		

HMTBa = 2-hydroxy-4-(methylthio)butanoate. ^1^ Complete range of values and expressed relative to response obtained with manganese sulphate except where noted. ^2^ Feed-grade oxide and sulphate used to obtain relative values. ^3^ Extended details on each source including response criterion, method of calculation, diet type, added level (mg kg^−1^) and original source references are available in the supplementary data tables.

**Table 7:** Multi-species table of relative bioavailability values (%) for zinc ^1,2^.

Source	Cattle	Poultry	Sheep	Swine
Fe-ZnSO_4_.H_2_O		107		
Zinc acetate				
Zin amino acid complex/chelate		76–164	102–110	102
Zinc chloride	42	88–107		
Zinc chloride, basic		108–119		
Zinc chloride, tetrabasic (TBZC)		102–111		122–159
Zinc sulphate (incl: basic & tribasic)	100	76–124	83–99	
Zinc aspartate				
Zinc carbonate	58	78–123	105–106	98
Zinc, chelated			91–125	
Zinc citrate		128		
Zinc EDTA		110–118	17	
Zinc, elemental		102		
Zinc glycine			82–335	
Zinc lysine	100	106–111	114	24–110
Zinc methionine	98–133	77–292	95–134	60–116
Zinc methionine hydroxy analog (ZnHMTBa)		161–441		
Zinc oxide	98–101	22–108	74–106	50–110
Zinc picolinate		31–104		
Zinc polysaccharide complex	144	94		
Zinc propionate		116–119		
Zinc proteinate		70–200	56–254	
Zinc, sequestered			97–108	
Zn-FeSO_4_.H_2_O		99		

TBZC = Zinc chloride hydroxide monohydrate (or tetrabasic zinc chloride); EDTA = ethylenediaminetetraacetic acid; HMTBa = 2-hydroxy-4-(methylthio)butanoate. ^1^ Complete range of values and expressed relative to response obtained with sulphate, chloride or acetate forms of zinc. Terminology for sources is that of the author(s). ^2^ Extended details on each source including response criterion, method of calculation, diet type, added level (mg kg^−1^) and original source references are available in the supplementary data tables.

**Table 8:** Relative absorption values with published Q_f_ values ^1^.

Source	RV1, %	Q_f_	Standard	Response Criterion	MethodCalc. ^2^	Type Diet	Added Level, mg kg^−1^	Reference
Mn amino acid complex A (M) (6.48% Mn)	114–273	45.3	MnSO_4_.7H_2_O	Plasma Mn, Abs		N—21 mg kg^−1^	90	Ji et al. [273]
Mn amino acid complex B (S) (7.86% Mn)	129–360	115.4	MnSO_4_.7H_2_O	Abs, Plasma		N—21 mg kg^−1^	90	Ji et al. [273]
MnSO4 + Gly	111–318		MnSO_4_.7H_2_O	Abs		N—21 mg kg^−1^	90	Ji et al. [273]
MnSO4 + Met	150–305		MnSO_4_.7H_2_O	Abs		N—21 mg kg^−1^	90	Ji et al. [273]
Mn-Gly chelate	139–333		MnSO_4_.7H_2_O	Abs		N—21 mg kg^−1^	90	Ji et al. [273]
Mn-Met chelate	170–373		MnSO_4_.7H_2_O	Abs		N—21 mg kg^−1^	90	Ji et al. [273]
Mn-Met complex E (W) (8.27% Mn)	110–160	3.2	MnSO_4_.7H_2_O	Abs		N—21 mg kg^−1^	90	Ji et al. [273]
Mn amino acid complex A (M) (6.48% Mn)	98–182	45.3	MnSO_4_.7H_2_O	Abs		N—21 mg kg^−1^	90	Ji et al. [274]
Mn amino acid complex B (S) (7.86% Mn)	102–213	115.4	MnSO_4_.7H_2_O	Abs		N—21 mg kg^−1^	90	Ji et al. [274]
MnSO4 + Gly	52–90		MnSO_4_.7H_2_O	Abs		N—21 mg kg^−1^	90	Ji et al. [274]
MnSO4 + Met	75–194		MnSO_4_.7H_2_O	Abs		N—21 mg kg^−1^	90	Ji et al. [274]
Mn-Gly chelate	82–159		MnSO_4_.7H_2_O	Abs		N—21 mg kg^−1^	90	Ji et al. [274]
Mn-Met chelate	161–230		MnSO_4_.7H_2_O	Abs		N—21 mg kg^−1^	90	Ji et al. [274]
Mn-Met complex E (W) (8.27% Mn)	80–168	3.2	MnSO_4_.7H_2_O	Abs		N—21 mg kg^−1^	90	Ji et al. [274]
Mn amino acid complex A (M) (6.48% Mn) d31, normal Ca	133–164	45.3	MnSO_4_.7H_2_O	Abs		N—21 mg kg^−1^	90	Ji et al. [274]
Mn amino acid complex A (M) (6.48% Mn) d31, high Ca	100–117	45.3	MnSO_4_.7H_2_O	Abs		N—21 mg kg^−1^	90	Ji et al. [274]
Mn amino acid complex B (S) (7.86% Mn) d31, normal Ca	145–191	115.4	MnSO_4_.7H_2_O	Abs		N—21 mg kg^−1^	90	Ji et al. [274]
Mn amino acid complex B (S) (7.86% Mn) d31, high Ca	107–165	115.4	MnSO_4_.7H_2_O	Abs		N—21 mg kg^−1^	90	Ji et al. [274]
Mn-Met complex E (W) (8.27% Mn) d31, normal Ca	108–182	3.2	MnSO_4_.7H_2_O	Abs		N—21 mg kg^−1^	90	Ji et al. [274]
Mn-Met complex E (W) (8.27% Mn) d31, high Ca	106–143	3.2	MnSO_4_.7H_2_O	Abs		N—21 mg kg^−1^	90	Ji et al. [274]
Mn AA complex (OW)	103–113	2.35	MnSO_4_.7H_2_O RG	Plasma Mn		N—14 mg kg^−1^	110	Liao et al. [272]
Mn AA chelate (OM)	125–141	61.9	MnSO_4_.H_2_O RG	Plasma Mn		N—14 mg kg^−1^	110	Liao et al. [272]
Mn AA proteinate (OS)	136–169	147	MnSO_4_.H_2_O RG	Plasma Mn		N—14 mg kg^−1^	110	Liao et al. [272]
Mn methionine E (W) (8.27% Mn)	102–103	3.2	MnSO_4_.H_2_O RG	Bone, Heart MnSOD mRNA, Bone		N—16 mg kg^−1^	120	Luo et al. [334]
Mn amino acid B (M) (6.48% Mn)	98–110	45.3	MnSO_4_.H_2_O RG	Bone, Heart MnSOD mRNA		N—16 mg kg^−1^	120	Luo et al. [334]
Mn amino acid C (S) (7.86% Mn)	99–102	115.4	MnSO_4_.H_2_O RG	Bone, Heart MnSOD mRNA		N—16 mg kg^−1^	120	Luo et al. [334]
Mn AA A (W)	99–105	2.35	MnSO_4_.H_2_O RG	Heart MnSOD mRNA, Heart Mn, MnSOD protein conc., MnSOD activity		N—16 mg kg^−1^	100–200	Li et al. [333]
Mn AA B (M)	104–118	16.85	MnSO_4_.H_2_O RG	Heart MnSOD mRNA, Heart Mn, MnSOD protein conc., MnSOD activity		N—16 mg kg^−1^	100–200	Li et al. [333]
Mn AA C (S)	102–112	147	MnSO_4_.H_2_O RG	Heart MnSOD mRNA, Heart Mn, MnSOD protein conc., MnSOD activity		N—16 mg kg^−1^	100–200	Li et al. [333]
Mn AA (M) (9.06% Mn) (d7, d14)	133, 136	16.85	MnSO_4_.H_2_O RG	Plasma Mn		N—13 mg kg^−1^	100	Bai et al. [275]
Mn AA (S) (10.18% Mn) (d7, d14)	146, 175	147	MnSO_4_.H_2_O RG	Plasma Mn		N—13 mg kg^−1^	100	Bai et al. [275]
Zinc amino acid complex C (W) (11.93% Zn)	105–162	6.48	ZnSO_4_.7H_2_O RG	Abs		N—90 mg kg^−1^	40	Yu et al. [331]
Zn-Gly chelate	109–160		ZnSO_4_.7H_2_O RG	Abs		N—90 mg kg^−1^	40	Yu et al. [331]
Zn-Met chelate	109–146		ZnSO_4_.7H_2_O RG	Abs		N—90 mg kg^−1^	40	Yu et al. [331]
Zinc proteinate A (S) (18.61% Zn)	112–196	944.02	ZnSO_4_.7H_2_O RG	Abs		N—90 mg kg^−1^	40	Yu et al. [331]
Zinc proteinate B (M) (13.27% Zn)	108–189	30.73	ZnSO_4_.7H_2_O RG	Abs		N—90 mg kg^−1^	40	Yu et al. [331]
ZnSO_4_.7H_2_O + Gly	77–97		ZnSO_4_.7H_2_O RG	Abs		N—90 mg kg^−1^	40	Yu et al. [331]
ZnSO_4_.7H_2_O + Met	88–99		ZnSO_4_.7H_2_O RG	Abs		N—90 mg kg^−1^	40	Yu et al. [331]

(M) = moderate; (S) = Strong; (W) = weak; N = natural diet; Abs = absorption; AA = amino acid; MnSOD = manganese superoxide dismutase activity; mRNA = messenger ribonucleic acid. ^1^ Values are relative absorption values not relative bioavailability values but there is correlation. ^2^ As the values are not relative bioavailability values, the method calculated is based on absorption and as such this column has been left blank to prevent confusion with later methods for RBV calculation.

## Data Availability

All data and related tables generated during this review are included in the published review and its supplementary information files.

## Supplementary Materials

The following supporting information can be downloaded at: https://www.mdpi.com/article/10.3390/ani12151981/s1, Individual species tables for relative bioavailability of supplemental copper sources: Table S1. Cattle; Table S2. Poultry; Table S3. Sheep; Table S4. Swine Individual species tables for relative bioavailability of supplemental iron sources; Table S5. Cattle; Table S6. Poultry; Table S7. Sheep; Table S8. Swine; Individual species tables for relative bioavailability of supplemental manganese sources; Table S9. Poultry; Table S10. Sheep; Table S11. Swine; Individual species tables for relative bioavailability of supplemental zinc sources; Table S12. Cattle; Table S13. Poultry—Chickens; Table S14. Poultry—Turkeys; Table S15. Poultry—Japanese Quail; Table S16. Sheep; Table S17. Swine.

## Abbreviations

AA	Amino acid
AAFCO	Association of American Feed Control Officials
Abs	Absorption
ACTH	Adrenocorticotropic hormone
BRD	Bovine respiratory disease
CAT	Cationic amino acid transporter
DM	Dry matter
DMT	Divalent metal transporter
EDTA	Ethylenediaminetetraacetic acid
EFSA	European Food Safety Authority
FP	Ferroportin
GLM	General linear model
HMTBa	2-hydroxy-4-(methylthio)butanoate
ITM	Inorganic trace mineral(s)
MCT1	Monocarboxylate transporter
MnSOD	Manganese superoxide dismutase activity
mRNA	Messenger ribonucleic acid
MT	Metallothionein
OTM	Organic trace mineral(s)
Qf	Formation quotient
RBV	Relative bioavailability value
SBM	Soybean meal
SOD	Superoxide dismutase
SPC	Soy protein concentrate
TBCC	dicopper chloride trihydroxide (or tribasic copper chloride)
TBZC	Zinc chloride hydroxide monohydrate (or tetrabasic zinc chloride)
