# Supplementary material for: Relative Bioavailability of Trace Minerals in Production Animal Nutrition: A Review
Source: Animals (Basel). 2022 Aug 4;12(15):1981. doi: 10.3390/ani12151981 (PMC9367456; doi:10.3390/ani12151981)
Supplement: Supplementary file 1 [file animals-12-01981-s001.zip › animals-1807974-supplementary.pdf]

animals MDPI

Relative bioavailability of trace minerals in production animal nutrition: A review

Laurann Byrne* and Richard A. Murphy

Alltech Bioscience Centre, Summerhill Road, Dunboyne, Co. Meath, Ireland. A86 X006

*Corresponding author

E-mail address: lbyrne@alltech.com (L. Byrne)

Supplementary Data tables – S1 – S17

Notes on tables S1 – S17:

- Early data is based on previously published tables by Ammerman et al. [1].
- Data from tables published by De Groote et al. [2] have also been incorporated and are identified by an asterix (*). Some of this data include earlier Ammerman data but were calculated using different metrics, causing RBV to vary in some cases.
- The remaining data has been taken directly from peer-reviewed publications as per the reference column.
- Source names and spelling are those used by the authors in the text of the respective papers and may differ between publications.
- Chemical formula for a compound is given only if provided by the author.
- The most recent database search was carried out in May 2022.
- Tables contain data for production animals only.

COPPER

Individual species tables for relative bioavailability of supplemental copper sources

Table S1. Cattle

Source	RV, %	Standard	Response Criterion	Method Calculation	Type diet	Added level, mg/kg	Reference

Copper carbonate	86*	Cupric sulfate	Liv Cu			10*, 50 mg / day	Ward et al., 1996 [3]
Copper chloride	98*	Cupric sulfate	Liv Cu			20	Engle and Spears, 2000 [4]
Copper chloride basic Diet: (high S (0.25 %) and Mo (6.8 mg/kg DM)	112	$CuSO_4.5H_2O$	Liv Cu	SR	N -5 mg/kg	5, 10	VanValin et al., 2019 [5]
Copper chloride basic Diet: (high S (0.25 %) and Mo (6.8 mg/kg DM)	102	$CuSO_4.5H_2O$	Pla Cu	SR	N -5 mg/kg	5, 10	VanValin et al., 2019 [5]
Copper citrate	101*	Cupric sulfate	Liv Cu			20	Engle and Spears, 2000 [4]
Copper glycinate Phase 1 (2mg Mo) Phase 2 (6 mg Mo)	140, 144	Copper sulfate FG	Pla Cu	SR	N – 8 mg/kg	5, 10	Hansen et al., 2008 [6]
Copper glycinate Phase 1 (2mg Mo) Phase 2 (6 mg Mo)	131, 150	Copper sulfate FG	Liv Cu	SR	N – 8 mg/kg	5, 10	Hansen et al., 2008 [6]
Copper glycinate Phase 1 (2mg Mo) Phase 2 (6 mg Mo)	140, 157	Copper sulfate FG	Cer	SR	N – 8 mg/kg	5, 10	Hansen et al., 2008 [6]
Copper-lysine	100, 102* 112	Cupric sulfate	Pla Cu	TP	N -5 mg/kg	30 mg/day	Kegley and Spears, 1994 [7]
Copper lysine	104*	Cupric sulfate	Liv Cu			15 – 30	Chase et al., 2000 [8]
Copper lysine[1]	153*	Cupric sulfate	Apparent abs.			8	Nockels et al., 1993 [9]
Copper lysine	89*	Cupric sulfate	Pla Cu		N	5	Ward et al., 1993 [10]
Copper lysine Diet: (high S (0.25 %) and Mo (6.8 mg/kg DM)	103	$CuSO_4.5H_2O$	Liv Cu	SR	N -5 mg/kg	5, 10	VanValin et al., 2019 [5]
Copper lysine	101	$CuSO_4.5H_2O$	Pla Cu	SR	N -5 mg/kg	5, 10	VanValin et al., 2019 [5]

Diet: (high S (0.25 %) and Mo (6.8 mg/kg DM)							
Copper proteinate	112	Cupric sulfate	Pla Cu[2]	MR	N – 5 mg/kg	14	Kincaid et al., 1986 [11]
Copper proteinate	147, 107*	Cupric sulfate	Liv Cu[2]	MR	N – 5 mg/kg	14, 18*	Kincaid et al., 1986 [11]
Copper proteinate	102 (110, 82)	Cupric sulfate	Liv Cu	MR	N	10	Wittenberg et al., 1990 [12]
Copper proteinate	102*	Cupric sulfate	Liv Cu, Pla			5 – 80	Du et al., 1996 [13]
Copper proteinate	103*	Cupric sulfate	Liv Cu			20	Engle and Spears, 2000 [4]
Copper proteinate	100*	Cupric sulfate	Liv Cu			10*, 50 mg/ day	Ward et al., 1996 [3]
Cupric chloride	121	Cupric sulfate	Liv Cu	TP	N-6 mg/kg	14-16	Ivan et al., 1990 [14]
Cupric chloride	114, 102*	Cupric sulfate	Liv Cu	TP	N-6 mg/kg	14-16	Ivan et al., 1990 [14]
Cupric oxide	25	Cupric sulfate	Liv Cu	TP	N-7 mg/kg	18	Xin et al., 1991 [15]
Cupric oxide	0, 64*, 7	Cupric sulfate	Pla Cu	TP	N – 5 mg/kg	30 mg/day	Kegley and Spears, 1994 [7]
$Na_2CuEDTA.3H_2O$	91	Cupric sulfate	Liv Cu	MR	N-7 mg/kg	105 mg/day	Miltimore et al., 1978 [16]
$Na_2CuEDTA.3H_2O$	104	Cupric sulfate	Liv Cu	MR	N-7 mg/kg	105 mg/day	Miltimore et al., 1978 [16]
Tribasic copper chloride (TBCC) Cu_2OH_3Cl	121[3]	Cupric sulfate	Pla Cu				Spears et al., 1997 [17]
Tribasic copper chloride (TBCC) Cu_2OH_3Cl	196[3]	Cupric sulfate	Liv Cu				Spears et al., 1997 [17]
Tribasic copper chloride (TBCC) Cu_2OH_3Cl	132	$CuSO_4.5H_2O$ FG	Pla Cu	SR	N – 5 mg/kg (+ High Mo + S)	5, 10	Spears et al., 2004 [18]

Tribasic copper chloride (TBCC) Cu_2OH_3Cl	118	$CuSO_4.5H_2O$ FG	Pla Cer	SR	N – 5 mg/kg (+ High Mo + S)	5, 10	Spears et al., 2004 [18]
Tribasic copper chloride (TBCC) Cu_2OH_3Cl	196	$CuSO_4.5H_2O$ FG	Liv Cu	SR	N – 5 mg/kg (+ High Mo + S)	5, 10	Spears et al., 2004 [18]
Tribasic copper chloride (TBCC) Cu_2OH_3Cl (Exp. 2, Cu depleted steers)	104	$CuSO_4.5H_2O$ FG	Pla Cu	SR	N – 5 mg/kg	50, 100	Spears et al., 2004 [18]
Tribasic copper chloride (TBCC) Cu_2OH_3Cl (Exp. 2, Cu depleted steers)	110	$CuSO_4.5H_2O$ FG	Pla Cer	SR	N – 5 mg/kg	50, 100	Spears et al., 2004 [18]
Tribasic copper chloride (TBCC) Cu_2OH_3Cl (Exp. 2, Cu depleted steers)	87	$CuSO_4.5H_2O$ FG	Liv Cu	SR	N – 5 mg/kg	50, 100	Spears et al., 2004 [18]

Liv = liver; Pla = plasma, SR = slope ratio; N = natural; FG = feed grade; abs = absorption; Cer = ceruloplasmin; MR = mean ratio; TP = three-point

[1] A large SD was observed for copper lysine due to the important difference in the apparent absorption efficiency compared to the reference source (Nockels et al., 1993). Without this observation the RBV of copper lysine becomes 98 % ± 5.3 %.

[2] Value for standard source was ≤ that for unsupplemented control group

[3] The higher bioavailability of copper from tribasic copper chloride may relate to the low solubility of copper chloride in the rumen environment, which may reduce the potential for copper to interact with molybdenum and sulfur in the rumen. Tribasic copper chloride and cupric sulfate were similar in bioavailability when evaluated in copper-deficient steers fed diets that were low in molybdenum (Spears et al., 1997)

Table S2. Poultry

**Source**	**RV, %**	**Q_f**	**Standard**	**Response Criterion**	**Method Calculation**	**Type diet**	**Added level, mg/kg**	**Reference**
Copper chloride, tribasic, TBCC (59.5 % Cu)	93, 84		$CuSO_4.H_2O$	Liv Cu, Feather Cu conc.	SR	N – 13 mg/kg	100, 200, 300	Kim et al., 2015 [19]
Copper chloride, tribasic, TBCC (62.9 % Cu)	107, 70		$CuSO_4.H_2O$	Egg yolk Cu conc., Feather Cu conc.	SR	N – 15 mg/kg	100, 200, 300	Kim et al., 2016 [20]

Copper-lysine	99, 92*		Cupric sulfate RG ($CuSO_4.5H_2O$)	Liv Cu	SR	N -11 mg/kg	150, 300, 450	Pott et al., 1994 [21]
Copper-lysine	116, 107*		Cupric sulfate	Liv Cu	SR	N – 290 mg/kg	75, 150	Baker et al., 1991 [22]
Copper-lysine	120, 99*, 102*		Cupric sulfate	Bile Cu	SR	SP – 5 mg/kg	0.5, 1	Aoyagi and Baker, 1993b [23]
Copper-methionine	96		Cupric sulfate	Bile Cu	SR	SP – 0.5 mg/kg	0.5, 1	Aoyagi and Baker, 1993d [24]
Copper-methionine	88, 91*		Cupric sulfate	Liv Cu	SR		100, 200	Aoyagi and Baker, 1993d [24]
Copper methionine	117		Copper sulfate	Liv Cu	SR	N - 8	20, 40	Wen et al., 2019
Corn gluten meal	48		Cupric sulfate	Bile Cu	SR	SP – 0.5 mg/kg	1	Aoyagi et al., 1993 [25]
Cottonseed meal	41		Cupric sulfate	Bile Cu	SR	SP – 0.5 mg/kg	1	Aoyagi et al., 1993 [25]
Cu amino acid chelate (8.92 % Cu)	122	3.2	Cupric sulfate RG	Liv Cu	SR	N – 16 mg/kg	150, 300, 450	Guo et al., 2001a [26]
Cu amino acid chelate	128		Cupric sulfate	Liv Cu	SR		150, 300, 450	Guo et al., 2001b [27]
Cu amino acid chelate	96		Cupric sulfate RG ($CuSO_4.5H_2O$)	Liv Cu	SR	N – 18 mg/kg	150, 300, 450	Miles et al., 2003 [28]
Cu chelate of 2-hydroxy-4(methylthio) butanoic acid (HMTBa)	112 (d 14), 111 (d 35)		$CuSO_4.7H_2O$ RG	Liv Cu	SR	N	10, 25, 50, 125, 250, 500	Wang et al., 2007 [29]
Cu Lysine (Exp. 1), (10.1 % Cu)	124,	2.8	Cupric sulfate	Liv Cu	SR	N – 16 mg/kg	150, 300, 450	Guo et al., 2001a [26]
Cu Lysine (Exp. 2), (10.1 % Cu)	111[1]		Cupric sulfate	Liv Cu	SR	N – 22 mg/kg	150, 300, 450	Guo et al., 2001a [26]
Cu oxide	76*		Cupric sulfate	Liv Cu	SC	N	1, 2	McNaughton et al., 1974 [30]
Cu proteinate A (10.21 % Cu) (Exp. 2)	109	1.1	Cupric sulfate	Liv Cu	SR	N – 22 mg/kg	150, 300, 450	Guo et al., 2001a [26]
Cu proteinate B (8.68 % Cu) (Exp. 2)	105	160,000	Cupric sulfate	Liv Cu	SR	N – 22 mg/kg	150, 300, 450	Guo et al., 2001a [26]

Cu proteinate C (9.33 % Cu)	111	220,000	Cupric sulfate	Liv Cu	SR	N – 16 mg/kg	150, 300, 450	Guo et al., 2001a [26]
Cu proteinate	99		Cupric sulfate	Liv Cu	SR		150, 300, 450	Guo et al., 2001b [27]
Cu proteinate (11.7 % Cu)	79	1.52	Cupric sulfate RG	Liv Cu	SR	N – 6 mg/kg	125, 250	Liu et al., 2012 [31]
Cu proteinate (11.7 % Cu)	79	1.52	Cupric sulfate RG	Bile Cu	SR	N – 6 mg/kg	125, 250	Liu et al., 2012 [31]
Cupric acetate	188		Cupric sulfate	Liv Cu	TP	N – 16 mg/kg	720	Norvell et al., 1974 [32]
Cupric acetate	93*		Cupric sulfate FG	Liv Cu	SR	N – 11 mg/kg	150 – 450	Ledoux et al., 1987 [33]
Cupric acetate RG (31.8 % Cu)	99, 112*, 100		Cupric sulfate FG	Liv Cu	SR	N – 11 mg/kg	150 – 450	Ledoux et al., 1991 [34]
Cupric basic carbonate ($CuCO_3.Cu(OH)_2$)	113		Cupric sulfate	Bile Cu	SR	SP – 0.5 mg/kg	0.5, 1	Aoyagi and Baker, 1993a [35]
Cupric carbonate FG	66, 68*		Cupric acetate RG	Liv Cu	SR	N – 5 mg/kg	5, 10, 20	Zanetti et al., 1991 [36]
Cupric carbonate FG	64*		Cupric acetate RG	Liv Cu	SR	N – 11 mg/kg	150, 300, 450	Ledoux et al., 1987 [33]
Cupric carbonate FG (54.6 % Cu)	68, 61* 54		Cupric acetate RG	Liv Cu	SR	N – 11 mg/kg	150, 300, 450	Ledoux et al., 1991 [34]
$Cu_2(OH)_3Cl$ FG	106		Cupric sulfate RG	Liv Cu	SR	N – 26	150, 300, 450	Ammerman et al., 1995 [1]
$Cu_2(OH)_3Cl$ (Exp.1, 21 d)	103*, 106		Cupric sulfate	Liv Cu	SR	N – 26	150, 300, 450	Miles et al., 1998 [37]
$Cu_2(OH)_3Cl$ (Exp. 2, 42 d)	112		Cupric sulfate	Liv Cu	SR	N – 20 (S), N – 11 (G)	200, 400, 600	Miles et al., 1998 [37]
$Cu_2(OH)_3Cl$	100		Cupric sulfate	Liv Cu	SR	N – 26	150, 300, 450	Miles et al., 1998 [37]
Cupric chloride, tribasic [$Cu_2(OH)_3Cl$] TBCC	112		Cupric sulfate	Liv Cu	SR		150, 300, 450	Guo et al., 2001b [27]
Cupric chloride, tribasic [$Cu_2(OH)_3Cl$] TBCC (56.7 % Cu)	109		$CuSO_4.5H_2O$ RG	Liv Cu	SR	N – 11 mg/kg	150, 300, 450	Luo et al., 2005 [38]
Cupric chloride, tribasic	134		$CuSO_4.5H_2O$	Egg weight	SR	N – 6 mg/kg	65, 130, 195, 260	Liu et al., 2005 [39]

[$Cu_2(OH)_3Cl$] TBCC (58 % Cu)								
Cupric chloride	110, 106		Cupric sulfate	Liv Cu	TP	N – 16 mg/kg	720	Norvell et al., 1974 [32]
Cupric chloride	106		Cupric sulfate	Liv Cu	TP	N – 15 mg/kg	720	Norvell et al., 1975 [40]
Cupric chloride, tribasic FG [$Cu_2(OH)_3Cl$] TBCC	100		Cupric sulfate RG	Liv Cu	SR	N	200, 400, 600	Miles et al., 1994 [41]
Cupric oxide FG	-5*		Cupric acetate RG	Liv Cu	SR	N – 11 mg/kg	150, 300, 450	Ledoux et al., 1987 [33]
Cupric oxide FG (74.1 % Cu)	< 1, 1* 0.54		Cupric acetate RG	Liv Cu	SR	N – 11 mg/kg	150, 300, 450	Ledoux et al., 1991 [34]
Cupric oxide	< 1, 0*		Cupric sulfate	Liv Cu	SR	N – 290 mg/kg	75, 150	Baker et al., 1991 [22]
Cupric oxide	0		Cupric sulfate	Bile Cu	SR	SP – 0.5 mg/kg	1, 2	Aoyagi and Baker, 1993a [35]
Cupric oxide	0		Cupric sulfate	Liv Cu	TP	N – 15 mg/kg	720	Norvell et al., 1975 [40]
Cupric oxide RG	1		Cupric sulfate RG	Liv Cu	TP	N – 14 mg/kg	750	Jackson and Stevenson, 1981 [42]
Cupric oxide RG	69*		Cupric sulfate RG	Liv Cu	TP	N – 14 mg/kg	750	Jackson and Stevenson, 1981 [42]
Cupric Sulfate FG	100*		Cupric acetate RG	Liv Cu	SR	N – 11 mg/kg	150, 300, 450	Ledoux et al., 1987 [33]
Cupric Sulfate FG (25.1% Cu)	98, 100*, 89		Cupric acetate RG	Liv Cu	SR	N – 11 mg/kg	150, 300, 450	Ledoux et al., 1991 [34]
Cuprous chloride	143		Cupric sulfate	Bile Cu	SR	SP – 0.5 mg/kg	0.5, 1	Aoyagi and Baker, 1993a [35]
Cuprous chloride	145, 81*		Cupric sulfate	Liv Cu	SR		50, 100	Aoyagi and Baker, 1993d [24]
Cuprous iodide	46, 82*		Cupric sulfate	Liv Cu	SC	N	1, 2	McNaughton et al., 1974 [30]
Cuprous oxide	92, 98*		Cupric sulfate	Liv Cu	SR	N – 290 mg/kg	75 – 150	Baker et al., 1991 [22]

Cuprous oxide	98		Cupric sulfate	Bile Cu	SR	SP – 0.5 mg/kg	0.5, 1	Aoyagi and Baker, 1993a [35]
Feather meal	< 1		Cupric sulfate	Bile Cu	SR	SP – 0.5 mg/kg	0.5	Aoyagi et al., 1995 [43]
Hair meal, swine	9		Cupric sulfate	Bile Cu	SR	SP – 0.5 mg/kg	1	Aoyagi et al., 1995 [43]
Liver, beef	82		Cupric sulfate	Bile Cu	SR	SP – 0.5 mg/kg	1	Aoyagi et al., 1993 [25]
Liver, pork	< 1		Cupric sulfate	Bile Cu	SR	SP – 0.5 mg/kg	1	Aoyagi et al., 1993 [25]
Liver, poultry	105		Cupric sulfate	Bile Cu	SR	SP – 0.5 mg/kg	1	Aoyagi et al., 1993 [25]
Liver, sheep	113		Cupric sulfate	Bile Cu	SR	SP – 0.5 mg/kg	1	Aoyagi et al., 1993 [25]
Meat and bone meal, beef	4		Cupric sulfate	Bile Cu	SR	SP – 0.5 mg/kg	1	Aoyagi et al., 1995 [43]
Meat and bone meal, mixed	28		Cupric sulfate	Bile Cu	SR	SP – 0.5 mg/kg	1	Aoyagi et al., 1995 [43]
Meat and bone meal, pork	53		Cupric sulfate	Bile Cu	SR	SP – 0.5 mg/kg	1	Aoyagi et al., 1995 [43]
Peanut hulls	44		Cupric sulfate	Bile Cu	SR	SP – 0.5 mg/kg	1	Aoyagi et al., 1993 [25]
Pork liver (Barrow, Gilt)	0		$CuSO_4.5H_2O$	Bile Cu	SR	SP – 0.6	1	Aoyagi et al., 1995 [43]
Poultry by-product meal	67		Cupric sulfate	Bile Cu	SR	SP – 0.5 mg/kg	1	Aoyagi et al., 1993 [25]
Soybean meal	38		Cupric sulfate	Bile Cu	SR	SP – 0.5 mg/kg	1	Aoyagi et al., 1993 [25]
Soybean mill run	47		Cupric sulfate	Bile Cu	SR	SP – 0.5 mg/kg	1	Aoyagi et al., 1993 [25]
Swine feces	36		Cupric sulfate	Liv Cu	SR	SP – 20 mg/kg	250 – 748	Izquierdo and Baker, 1986 [44]

Liv = liver; SR = slope ratio; N = natural; SP = semi-purified; RG = reagent grade; FG = feed grade; abs = absorption; SC = standard curve; TP = three-point

[1] Guo et al. reported a RBV difference of 13 percentage points between Exp. 1 and Exp. 2 for Cu Lys which they proposed was due to different strains of chicks used in the 2 experiments

Table S3. Sheep

Source	RV, %	Standard	Response Criterion	Method Calculation	Type diet	Added level, mg/kg	Reference
Copper AA complex	100*	Cupric sulfate	Liv Cu			10	Hatfield et al., 2001 [45]
Copper acetate	93, 100*	Cupric chloride RG / $CuSO_4.5H_2O$	Liv Cu	SR		60 – 120	Ledoux et al., 1995 [46], EMFEMA [2]
Copper carbonate	121, 97*	Cupric chloride RG / $CuSO_4.5H_2O$	Liv Cu	SR		60 – 120	Ledoux et al., 1995 [46], EMFEMA [2]
Copper chloride	100, 96*	Cupric chloride RG / $CuSO_4.5H_2O$	Liv Cu	SR		60 – 120	Ledoux et al., 1995 [46], EMFEMA [2]
Copper EDTA	96	Cupric sulfate	Liv Cu	TP	N	70 mg Cu 1x/wk	MacPherson and Hemingway, 1968 [47]
Copper glycine	96	Cupric sulfate	Liv Cu	TP	N	70 mg Cu 1x/wk	MacPherson and Hemingway, 1968 [47]
Copper lysine	97*, 93	Cupric sulfate	Liv Cu	SR	N – 10 mg/kg	180	Luo et al., 1996 [48]
Copper lysine	97*, 68	Cupric sulfate	Liv Cu		N – 10 mg/kg	60, 120, 180	Pott et al., 1994 [21]
Copper methionine FG	152	Cu sulfate FG	Liv Cu	SR	N – 9 mg/kg	15	Pal et al., 2010 [49]
Copper methionine FG	150	Cu sulfate FG	Pla Cu	SR	N – 9 mg/kg	15	Pal et al., 2010 [49]
Copper methionine FG	151	Cu sulfate FG	Gut abs.	SR	N – 9 mg/kg	15	Pal et al., 2010 [49]
Copper oxide	81*	Cupric chloride RG / $CuSO_4.5H_2O$	Liv Cu	SR		60 – 120	Ledoux et al., 1995 [46], EMFEMA [2]
Copper proteinate	103*	Cupric sulfate	Liv Cu, Kid, Pla			10 -20 -30	Eckert et al., 1999 [50]
Cupric acetate RG, Exp. 3	93	Cupric chloride RG	Liv Cu	SR	N – 13 mg/kg	120	Ledoux et al., 1995 [46]
Cupric carbonate FG, Exp. 2	121	Cupric chloride RG	Liv Cu	SR	N – 8 mg/kg	120	Ledoux et al., 1995 [46]

Cupric chloride	123, 118*	Cupric sulfate	Liv Cu	TP	N – 7 mg/kg	29	Charmley and Ivan, 1989 [51]
Cupric chloride	105, 102*	Cupric sulfate	Liv Cu	TP	N – 9 mg/kg	5, 10	Ivan et al., 1990 [14]
Cupric oxide FG, Exp. 2	35, 22	Cupric chloride RG	Liv Cu	SR	N – 8 mg/kg	120	Ledoux et al., 1995 [46]
Cupric oxide FG, Exp. 3	48	Cupric chloride RG	Liv Cu	SR	N – 13 mg/kg	120	Ledoux et al., 1995 [46]
Cupric sulfate FG, Exp. 2	175	Cupric chloride RG	Liv Cu	SR	N – 8 mg/kg	120	Ledoux et al., 1995 [46]
Cupric sulfate FG, Exp. 3	110	Cupric chloride RG	Liv Cu	SR	N – 13 mg/kg	120	Ledoux et al., 1995 [46]
Cupric sulfide	35	Cupric sulfate	Liv Cu	MR	N	30 mg/day	Dick, 1954 [52]
Cupric sulfide	11	Cupric sulfate	Pla Cu reg	MR	SP – 2 mg/kg	5	Suttle, 1974b [53]
Cuprous acetate	98, 110*	Cupric sulfate	Liv Cu	TP	N – 7 mg/kg	29	Charmley and Ivan, 1989 [51]
Swine feces	35	Cupric sulfate	Liv Cu	MR		30, 60	Prince et al., 1975 [54]
Swine feces	70	Cupric sulfate	Liv Cu	MR	N	4	Dalgarno and Mills, 1975 [55]
Swine feces	60	Cupric sulfate	Pla Cu	MR	N	4	Dalgarno and Mills, 1975 [55]
Swine feces	80	Cupric sulfate	Pla Cu	TP	SP	8	Suttle and Price, 1976 [56]
Swine feces	75	Cupric sulfate	Pla Cu	TP	SP	4	Suttle and Price, 1976 [56]
Swine feces	25	Cupric sulfate	Liv Cu	TP	N – 8 mg/kg	13 – 16	Poole et al., 1990 [57]

Liv = liver; Pla = plasma; AA = amino acid; abs = absorption; Kid = kidney; reg = regeneration; SR = slope ratio; MR = mean ratio; N = natural; SP = semi-purified; RG = reagent grade; TP = three-point; EDTA = ethylenediaminetetraacetate

Table S4. Swine

**Source**	**RV, %**	**Standard**	**Response Criterion**	**Method Calculation**	**Type diet**	**Added level, mg/kg**	**Reference**
Copper citrate + sodium molybdate	93, 99*	Cupric sulfate	Liv Cu	MR	N	20	Dowdy and Matrone, 1968 [58]
Copper citrate + sodium molybdate	74	Cupric sulfate	Cer	MR	N	20	Dowdy and Matrone, 1968 [58]

Copper chloride, tribasic	97*	Cupric sulfate	Liv Cu		12 mg/kg	20	Cromwell et al., 1998 [59]
Copper lysine	101	Cupric sulfate	Liv Cu		2 mg/kg	100, 150, 200	Apgar et al., 1995 [60]
Copper lysine	73	Cupric sulfate	Cu abs		3 mg/kg	200	Apgar et al., 1996 [61]
Copper lysine	101*	Cupric sulfate	Liv Cu		9 mg/kg	50, 100, 200, 250	Coffey et al., 1994 [62]
Copper methionine	107, 100*	Cupric sulfate	Liv Cu	TP	N – 10+ mg/kg	250	Bunch et al., 1965 [63]
Copper-molybdenum complex	68, 95*	Cupric sulfate	Liv Cu	MR	N	20	Dowdy and Matrone, 1968 [58]
Copper-molybdenum complex	0	Cupric sulfate	Cer	MR	N	20	Dowdy and Matrone, 1968 [58]
Copper proteinate (10 % Cu)	183	TBCC	ADG	SR	N	5, 10, 20, 40, 80, 160	Lin et al., 2020 [64]
Copper proteinate (10 % Cu)	172	TBCC	FCR	SR	N	5, 10, 20, 40, 80, 160	Lin et al., 2020 [64]
Copper proteinate (10 % Cu)	156	TBCC	ALP	SR	N	5, 10, 20, 40, 80, 160	Lin et al., 2020 [64]
Copper proteinate (10 % Cu)	263	TBCC	Cer	SR	N	5, 10, 20, 40, 80, 160	Lin et al., 2020 [64]
Copper proteinate (10 % Cu)	205	TBCC	Cu/Zn SOD	SR	N	5, 10, 20, 40, 80, 160	Lin et al., 2020 [64]
Copper proteinate (10 % Cu)	114	TBCC	GSH-Px	SR	N	5, 10, 20, 40, 80, 160	Lin et al., 2020 [64]
Copper proteinate (10 % Cu)	133	TBCC	MDA	SR	N	5, 10, 20, 40, 80, 160	Lin et al., 2020 [64]
Copper proteinate (10 % Cu)	208	TBCC	Jejunal Cu	SR	N	5, 10, 20, 40, 80, 160	Lin et al., 2020 [64]
Cupric carbonate	62, 92*	Cupric sulfate	Liv Cu	TP	N	250	Allen et al., 1961 [65]
Cupric carbonate	111, 108*	Cupric sulfate	^{64}Cu WB	MR	N – 15 mg/kg	Single oral dose – 10 mg	Buescher et al., 1961 [66]
Cupric carbonate,	99*	Cupric sulfate	Cer, Liv Cu			250	Bunch et al., 1965 [63]
Cupric oxide	89, 104*[1]	Cupric sulfate	^{64}Cu WB	MR	N – 15 mg/kg	Single oral dose	Buescher et al., 1961 [66]

Cupric oxide	15, 64*	Cupric sulfate	Liv Cu	MR	N – 3 mg/kg	250	Bunch et al., 1961 [67]
Cupric oxide	27	Cupric sulfate	Liv Cu	TP	N – 10 mg/kg	250	Bunch et al., 1963 [68]
Cupric oxide	55*	Cupric sulfate	Liv Cu		5	250	Bekaert et al., 1967 [69]
Cupric oxide	0, 75*	Cupric sulfate	Liv Cu	MR	N – 30 mg/kg	250	Cromwell et al., 1989 [70]
Cupric sulfide	0, 69*	Cupric sulfate	Liv Cu	TP	N	250	Barber et al., 1961 [71]
Cupric sulfide	33	Cupric sulfate	Cu^{64} upt	MR	N	250	Bowland et al., 1961 [72]
Cupric sulfide	23	Cupric sulfate	Cu^{64} upt	MR	N	250	Bowland et al., 1961 [72]
Cupric sulfide	<1, 53*	Cupric sulfate	Liv Cu	MR	N	250	Cromwell et al., 1978 [73]

Liv = liver; Cer = ceruloplasmin; abs = absorption; SR = slope ratio; MR = mean ratio; N = natural; TP = three-point; TBCC = dicopper chloride trihydroxide (tribasic copper chloride); Upt = uptake; ADG = average daily gain; FCR = feed conversion ratio; ALP = alkaline phosphatase; SOD = superoxide dismutase; GSH-Px = glutathione peroxidase; MDA = malondialdehyde; WB = whole blood

[1] Unexpectedly high value due to results by Buescher et al. showing cupric oxide had the same bioavailability as cupric sulfate using labelled copper, which gave value obtained from De Groote et al. (*) a large standard deviation (74 % ± 21 %). If this observation was omitted the RBV of Cu in CuO would have been 63 % ± 11 %.

IRON

Individual species tables for relative bioavailability of supplemental iron sources

Table S5. Cattle

Source	RV, %	Standard	Response Criterion	Method Calculation	Type diet	Added level, mg/kg	Reference
Ferric citrate	107	$FeSO_4$	Hb	TP	N – 10 mg/kg	30	Bremner and Dalgarno, 1973 [74]
Ferric EDTA	93	$FeSO_4$	Hb	TP	N – 10 mg/kg	30	Bremner and Dalgarno, 1973 [74]
Ferrous carbonate FG	0	$FeSO_4.H_2O$ FG	Liv Fe	TP	N – 180 mg/kg	1000	McGuire et al., 1985 [75]
Ferrous carbonate FG	79	$FeSO_4.H_2O$ FG	Kid Fe	TP	N – 180 mg/kg	1000	McGuire et al., 1985 [75]

Ferrous carbonate FG	23	$FeSO_4.H_2O$ FG	Spl Fe	TP	N – 180 mg/kg	1000	McGuire et al., 1985 [75]
Ferrous carbonate	25	Ferrous sulfate FG	Growth		N - 170	500, 1000, 2000, 4000	Miller et al., 1991 [76]
Iron phytate (17.7 % Fe)	47	$FeSO_4$	Hb	TP	N – 10 mg/kg	30	Bremner and Dalgarno, 1973 [74]

Hb = hemoglobin; Liv = liver; Kid = kidney; Spl = spleen; N = natural; FG = feed grade; TP = three-point; EDTA = ethylenediaminetetraacetate

Table S6. Poultry

Source	RV, %	Q_f	Standard	Response Criterion	Method Calculation	Type diet	Added level, mg/kg	Reference
Alfalfa meal (340 mg/kg Fe AD)	65		$FeSO_4.7H_2O$	Hb reg	SC	P	10 – 20	Chausow and Czarnecki-Maulden, 1988b [77]
Beef liver (235 mg/kg Fe AD)	90		$FeSO_4.7H_2O$	Hb reg	SR	P – 5 mg/kg	10, 20	Chausow and Czarnecki-Maulden, 1988a [78]
Beet pulp	26		Ferrous sulfate	Hb reg	SC	P – 10 mg/kg		Fly et al., 1998 [79]
Blood meal (1610 mg/kg Fe AD)	22		$FeSO_4.7H_2O$	Hb reg	SC	P	5 – 15	Chausow and Czarnecki-Maulden, 1988b [77]
Blood meal	35		$FeSO_4.7H_2O$ RG	Hb reg	SC[1]	SP	5 – 20	Pla and Fritz, 1970 [80]
Blood meal	35		$FeSO_4.H_2O$ RG	Hb reg	SC[1]	SP – 7 mg/kg	5 – 20	Fritz et al., 1970 [81]
Bone meal, steamed	0		$FeSO_4.7H_2O$ RG	Hb reg	SC	P – 5 mg/kg	5 – 20	Deming and Czarnecki-Maulden, 1989 [82]
Corn fiber, coarse	69		Ferrous sulfate	Hb reg	SC	P- 10 mg/kg		Fly et al., 1998 [79]
Corn germ meal	40		$FeSO_4.7H_2O$ RG	Hb reg	SC[1]	SP	5 – 20	Pla and Fritz, 1970 [80]
Corn germ	40		$FeSO_4.7H_2O$ RG	Hb reg	SC[1]	SP – 7 mg/kg	5 – 20	Fritz et al., 1970 [81]

Corn gluten meal (102 mg/kg Fe AD)	84		$FeSO_4.7H_2O$ RG	Hb reg	SC[1]	SP – 5 mg/kg	10, 20	Chausow and Czarnecki-Maulden, 1988a [78]
Corn, ground (100 mg/kg Fe AD)	20		$FeSO_4.7H_2O$ RG	Hb reg	SC	P	10 – 20	Chausow and Czarnecki-Maulden, 1988b [77]
Cottonseed meal (200 mg Fe / kg)	56		$FeSO_4.7H_2O$	Hb reg	SR	SP – 20 mg/kg	10 – 20	Boling et al., 1998 [83]
Defluorinated phosphate	48		$FeSO_4.7H_2O$ RG	Hb reg			7.5, 15.0, 22.5	Henry et al., 1992 [84]
Egg, yolk	33		$FeSO_4.7H_2O$ RG	Hb reg	SC[1]	SP – 7 mg/kg	5 – 20	Fritz et al., 1970 [81]
Feather meal (570 mg/kg Fe AD)	39		$FeSO_4.7H_2O$ RG	Hb reg	SC	P	5 – 15	Chausow and Czarnecki-Maulden, 1988b [77]
Fe proteinate (14.15 % Fe)[5]	117	43.6	$FeSO_4.7H_2O$ RG	Hb conc.	SR	P – 5 mg/kg	10, 20, 40	Ma et al., 2014 [85]
Fe proteinate (14.15 % Fe)	114	43.6	$FeSO_4.7H_2O$ RG	Total body Hb	SR	P – 5 mg/kg	10, 20, 40	Ma et al., 2014 [85]
Fe proteinate (14.15 % Fe)	103	43.6	$FeSO_4.7H_2O$ RG	Hematocrit	SR	P – 5 mg/kg	10, 20, 40	Ma et al., 2014 [85]
Fe proteinate (14.15 % Fe)	96	43.6	$FeSO_4.7H_2O$ RG	Liv Fe	SR	P – 5 mg/kg	10, 20, 40	Ma et al., 2014 [85]
Fe proteinate (14.15 % Fe)	100	43.6	$FeSO_4.7H_2O$ RG	Kid Fe	SR	P – 5 mg/kg	10, 20, 40	Ma et al., 2014 [85]
Fe methionine	88, 86*		$FeSO_4.7H_2O$ RG	Liv Fe	SR	P- 188 mg/kg	400, 600, 800	Cao et al., 1996 [86]
Fe – Met (W) (14.7 % Fe)	129	1.37	$FeSO_4.7H_2O$ RG	SDH mRNA liver	SR	N – 56 mg/kg	20, 40, 60	Zhang et al., 2016 [87]
Fe – Met (W) (14.7 % Fe)	102	1.37	$FeSO_4.7H_2O$ RG	SDH mRNA kidney	SR	N – 56 mg/kg	20, 40, 60	Zhang et al., 2016 [87]
Fe – Prot. (M) (14.2 % Fe)[5]	164	43.6	$FeSO_4.7H_2O$ RG	SDH mRNA liver	SR	N – 56 mg/kg	20, 40, 60	Zhang et al., 2016 [87]
Fe – Prot. (M) (14.2 % Fe)	143	43.6	$FeSO_4.7H_2O$ RG	SDH mRNA kidney	SR	N – 56 mg/kg	20, 40, 60	Zhang et al., 2016 [87]
Fe – Prot. (ES) (10.2 % Fe)	174	8,590	$FeSO_4.7H_2O$ RG	SDH mRNA liver	SR	N – 56 mg/kg	20, 40, 60	Zhang et al., 2016 [87]
Fe – Prot. (ES) (10.2 % Fe)	174	8,590	$FeSO_4.7H_2O$ RG	SDH mRNA kidney	SR	N – 56 mg/kg	20, 40, 60	Zhang et al., 2016 [87]

Fe-$ZnSO_4.H_2O$ (20.2 % Fe, 13.0 % Zn)	112*[3] 126[4]		Ferrous sulfate	Hb reg	SR	SP – 20 mg/kg	10, 20	Boling et al., 1998 [83]
Ferric ammonium citrate	107		$FeSO_4.7H_2O$ RG	Hb reg	SC[1]	SP	5 – 20	Pla and Fritz, 1970 [80]
Ferric ammonium citrate	115, 115* (98 – 115)[2]		$FeSO_4.7H_2O$ RG	Hb reg	SC[1]	SP	5 – 20	Fritz et al., 1970 [81]
Ferric chloride	44 (26 – 67)[2]		$FeSO_4.7H_2O$ RG	Hb reg	SC[1]	SP – 7 mg/kg	5 – 20	Fritz et al., 1970 [81]
Ferric chloride	78, 78*		$FeSO_4.7H_2O$ RG	Hb reg	SC[1]	SP	5 – 20	Pla and Fritz, 1970 [80]
Ferric choline citrate	102		$FeSO_4.7H_2O$ RG	Hb reg	SC[1]	SP – 7 mg/kg	5 – 20	Fritz et al., 1970 [81]
Ferric choline citrate	102		$FeSO_4.7H_2O$ RG	Hb reg	SC[1]	SP	5 – 20	Pla and Fritz, 1970 [80]
Ferric choline chloride	93*, 94		Ferrous sulfate	Hb reg	SC	SP	5 – 20	McNaughton et al., 1974 [30]
Ferric citrate	73 (70 – 76)[2]		$FeSO_4.7H_2O$ RG	Hb reg	SC[1]	SP – 7 mg/kg	5 – 20	Fritz et al., 1970 [81]
Ferric glycerophosphate	93 (86 – 100)[2]		$FeSO_4.7H_2O$ RG	Hb reg	SC[1]	SP – 7 mg/kg	5 – 20	Fritz et al., 1970 [81]
Ferric orthophosphate (0.8%) + Rice ($FePO_4.4H_2O$)	36		$FeSO_4.7H_2O$ RG	Hb reg	SR	P < 7 mg/kg	5 – 40	Amine et al., 1972 [88]
$FePO_4.4H_2O$	11, 13*		$FeSO_4.7H_2O$ RG	Hb reg	SR	P < 7 mg/kg	5 – 40	Amine et al., 1972 [88]
Ferric orthophosphate – 1	18, 13* (7-32)[2]		$FeSO_4.7H_2O$ RG	Hb reg	SC[1]	SP – 7 mg/kg	5 – 20	Fritz et al., 1970 [81]
Ferric orthophosphate – 2	9		$FeSO_4.7H_2O$ RG	Hb reg	SC[1]	SP – 7 mg/kg	5 – 20	Fritz et al., 1970 [81]
Ferric orthophosphate – 3	12		$FeSO_4.7H_2O$ RG	Hb reg	SC[1]	SP – 7 mg/kg	5 – 20	Fritz et al., 1970 [81]
Ferric orthophosphate $FePO_4.4H_2O$	15		$FeSO_4.7H_2O$ RG	Hb reg	SR	P < 7 mg/kg	10, 20, 40	Amine et al., 1972 [88]
Ferric orthophosphate (28.6 % Fe)	12		$FeSO_4.7H_2O$ RG	Hb reg	SC[1]	SP – 7 mg/kg	5 – 20	Pla and Fritz, 1970 [80]
Ferric orthophosphate	15		$FeSO_4.7H_2O$ RG	Hb reg	SC[1]	SP	5 – 20	Pla and Fritz, 1970 [80]
Ferric orthophosphate	12*		$FeSO_4.7H_2O$ RG	Hb reg	SC[1]	SP	5 – 20	Pla and Fritz, 1971 [89]

Ferric orthophosphate	4*		Ferrous sulfate	Hb reg				Pennell et al., 1976 [90]
Ferric oxide	4, 4* (0 – 6)[2]		$FeSO_4.7H_2O$ RG	Hb reg	SC[1]	SP – 7 mg/kg	5 – 20	Fritz et al., 1970 [81]
Ferric oxide Fe_2O_3	17		$FeSO_4.7H_2O$	Hb reg	TP	N	2 mg/day	Elvehjem et al., 1929 [91]
Ferric oxide (60 % Fe)	67		$FeSO_4.7H_2O$	Hb reg	SR	N	10 – 20	Poitevint, 1979 [92]
Ferric oxide	77*, 82		Ferrous sulfate	Hb reg	SC	N	5 – 20	McNaughton et al., 1974 [30]
Ferric pyrophosphate	45		$FeSO_4.7H_2O$ RG	Hb reg	SC[1]	SP	5 – 20	Pla and Fritz, 1970 [80]
Ferric pyrophosphate	45		$FeSO_4.7H_2O$ RG	Hb reg	SC[1]	SP – 7 mg/kg	5 – 20	Fritz et al., 1970 [81]
Ferric sulfate	65*		$FeSO_4.7H_2O$ RG	Hb reg	SC[1]	SP – 7 mg/kg	5 – 20	Fritz et al., 1970 [81]
Ferric sulfate	104		Ferrous sulfate	Hb reg	MR		60, 100	Moore et al., 1988 [93]
Ferric sulfate (22.7 % Fe) $Fe_2(SO_4)_3.7H_2O$	37*		$FeSO_4.7H_2O$	Hb reg	SR	SP – 20 mg/kg	10 – 20	Boling et al., 1998 [83]
Ferrous ammonium sulfate	99 (99 – 100)[2]		$FeSO_4.7H_2O$ RG	Hb reg	SC[1]	SP – 7 mg/kg	5 – 20	Fritz et al., 1970 [81]
Ferrous carbonate	8, 55*,		$FeSO_4.7H_2O$ RG	Hb reg	SR	P < 7 mg/kg	5 – 40	Amine et al., 1972 [88]
Ferrous carbonate	10		$FeSO_4.7H_2O$ RG	Hb reg	SR	P < 7 mg/kg	5 – 40	Amine et al., 1972 [88]
Ferrous carbonate – 1	2, 3* (0 – 6)[2]		$FeSO_4.7H_2O$ RG	Hb reg	SC[1]	SP – 7 mg/kg	5 – 20	Fritz et al., 1970 [81]
Ferrous carbonate – 2	2		$FeSO_4.7H_2O$ RG	Hb reg	SC[1]	SP – 7 mg/kg	5 – 20	Fritz et al., 1970 [81]
Ferrous carbonate – 3	6		$FeSO_4.7H_2O$ RG	Hb reg	SC[1]	SP – 7 mg/kg	5 – 20	Fritz et al., 1970 [81]
Ferrous carbonate – 4	2		$FeSO_4.7H_2O$ RG	Hb reg	SC[1]	SP – 7 mg/kg	5 – 20	Fritz et al., 1970 [81]
Ferrous carbonate – 1	2, 5*		$FeSO_4.7H_2O$ RG	Hb reg	SC[1]	SP	5 – 20	Pla and Fritz, 1970 [80]
Ferrous carbonate – 2	7		$FeSO_4.7H_2O$ RG	Hb reg	SC[1]	SP	5 – 20	Pla and Fritz, 1970 [80]
Ferrous carbonate – 1	2		$FeSO_4.7H_2O$ RG	Hb reg	SC[1]	SP	5 – 20	Pla and Fritz, 1970 [80]

Ferrous carbonate – 2	7		$FeSO_4.7H_2O$ RG	Hb reg	SC[1]	SP	5 – 20	Pla and Fritz, 1970 [80]
Ferrous carbonate – 3	0		$FeSO_4.7H_2O$ RG	Hb reg	SC[1]	SP	5 – 20	Pla and Fritz, 1970 [80]
Ferrous carbonate – 4	2		$FeSO_4.7H_2O$ RG	Hb reg	SC[1]	SP	5 – 20	Pla and Fritz, 1970 [80]
Ferrous carbonate (42 % Fe)	88		$FeSO_4.7H_2O$	Hb	SR	N	10 – 20	Poitevint, 1979 [92]
Ferrous carbonate ore (38 % Fe)	3, 3*		$FeSO_4.7H_2O$ RG	Hb reg	SC[1]	SP – 7 mg/kg	5 – 20	Pla and Fritz, 1971 [89]
Ferrous chloride	98		$FeSO_4.7H_2O$ RG	Hb reg	SC[1]	SP – 7 mg/kg	5 – 20	Fritz et al., 1970 [81]
Ferrous chloride	106, 106*		$FeSO_4.7H_2O$ RG	Hb reg	SC[1]	SP – 7 mg/kg	5 – 20	Pla and Fritz, 1971 [89]
Ferrous EDTA-dihydrogen	99 (97 – 100)[2]		$FeSO_4.7H_2O$	Hb reg	SC[1]	SP – 7 mg/kg	5 – 20	Fritz et al., 1970 [81]
Ferrous fumarate	95 (71 – 133)[2]		$FeSO_4.7H_2O$ RG	Hb reg	SC[1]	SP – 7 mg/kg	5 – 20	Fritz et al., 1970 [81]
Ferrous fumarate	102		$FeSO_4.7H_2O$ RG	Hb reg	SC[1]	SP – 7 mg/kg	5 – 20	Pla and Fritz, 1970 [80]
Ferrous gluconate	97		$FeSO_4.7H_2O$ RG	Hb reg	SC[1]	SP – 7 mg/kg	5 – 20	Fritz et al., 1970 [81]
Ferrous gluconate	97		$FeSO_4.7H_2O$ RG	Hb reg	SC[1]	SP – 7 mg/kg	5 – 20	Pla and Fritz, 1970 [80]
Ferrous sulfate, FG	100, 65*		$FeSO_4.7H_2O$ RG	Hb reg	SC[1]	SP – 7 mg/kg	5 – 20	Fritz et al., 1970 [81]
Ferrous sulfate ($FeSO_4.H_2O$) (31 %)	103		$FeSO_4.7H_2O$ RG	Hb reg	SR	N	10 – 20	Poitevint, 1979 [92]
Ferrous sulfate FG ($FeSO_4.H_2O$)	102		$FeSO_4.7H_2O$ RG	Hb reg	SR	P – 4 mg/kg	7.5 – 22.5	Ammerman et al., 1993 [94]
Ferrous sulfate FG ($FeSO_4.H_2O$)	92, 91*		$FeSO_4.7H_2O$ RG	Liv Fe	SR	N – 123 mg/kg	400, 600, 800	Cao et al., 1996 [86]
Ferrous sulfate monohydrate	102*		$FeSO_4.7H_2O$ RG	Hb reg			7.5, 15.0, 22.5	Henry et al., 1992 [84]
Ferrous sulfate, anhydrous	100		$FeSO_4.7H_2O$ RG	Hb reg	SC[1]	SP – 7 mg/kg	5 – 20	Fritz et al., 1970 [81]
Ferrous sulfate, encapsulated	97		$FeSO_4.7H_2O$ RG	Hb reg	SC[1]	SP – 7 mg/kg	5 – 20	Pla et al., 1973 [95]
Ferrous tartrate	77 (70 – 83)[2]		$FeSO_4.7H_2O$ RG	Hb reg	SC[1]	SP – 7 mg/kg	5 – 20	Fritz et al., 1970 [81]

Fishmeal (700 mg/kg Fe AD)	32		$FeSO_4.7H_2O$ RG	Hb reg	SC[1]	P	5 – 15	Chausow and Czarnecki-Maulden, 1988b [77]
Fish protein concentrate	22		$FeSO_4.7H_2O$ RG	Hb reg	SC[1]	SP – 7 mg/kg	5 – 20	Fritz et al., 1970 [81]
Hemoglobin	70		$FeSO_4.7H_2O$ RG	Hb reg	SR	P < 7 mg/kg	5 – 40	Amine et al., 1972 [88]
Hemoglobin, bovine	44		$FeSO_4.7H_2O$ RG	Hb reg	SC[1]	SP – 7 mg/kg	5 – 20	Pla et al., 1973 [95]
Iron, reduced (97.0 % Fe)	64		$FeSO_4.7H_2O$ RG	Hb reg	SR	P – 7 mg/kg	5 – 40	Amine et al., 1972 [88]
Iron, reduced	63, 36*		$FeSO_4.7H_2O$ RG	Hb reg	SR	P < 7 mg/kg	5 – 40	Amine et al., 1972 [88]
Iron, reduced – 1	59, 52* (8 – 66)[2]		$FeSO_4.7H_2O$ RG	Hb reg	SC[1]	SP – 7 mg/kg	5 – 20	Fritz et al., 1970 [81]
Iron, reduced – 2	41		$FeSO_4.7H_2O$ RG	Hb reg	SC[1]	SP – 7 mg/kg	5 – 20	Fritz et al., 1970 [81]
Iron, reduced – 3	66		$FeSO_4.7H_2O$ RG	Hb reg	SC[1]	SP – 7 mg/kg	5 – 20	Fritz et al., 1970 [81]
Iron, reduced – 4	43		$FeSO_4.7H_2O$ RG	Hb reg	SC[1]	SP – 7 mg/kg	5 – 20	Fritz et al., 1970 [81]
Iron, reduced (97.0 %)	18, 46*		$FeSO_4.7H_2O$ RG	Hb reg	SC[1]	SP – 7 mg/kg	5 – 20	Pla and Fritz, 1971 [89]
Iron, reduced – 1, 90 % < 10 µm	61		$FeSO_4.7H_2O$ RG	Hb reg	SC[1]	SP – 7 mg/kg	5 – 20	Pla et al., 1973 [95]
Iron, reduced – 2, 90 % < 10 µm	53		$FeSO_4.7H_2O$ RG	Hb reg	SC[1]	SP – 7 mg/kg	5 – 20	Pla et al., 1973 [95]
Iron, reduced – 3, 90 % < 10 µm	60		$FeSO_4.7H_2O$ RG	Hb reg	SC[1]	SP – 7 mg/kg	5 – 20	Pla et al., 1973 [95]
Iron, reduced – 4, 90 % < 10 µm	44		$FeSO_4.7H_2O$ RG	Hb reg	SC[1]	SP – 7 mg/kg	5 – 20	Pla et al., 1973 [95]
Iron, reduced – 5, 90 % < 10 µm	48		$FeSO_4.7H_2O$ RG	Hb reg	SC[1]	SP – 7 mg/kg	5 – 20	Pla et al., 1973 [95]
Iron, reduced – 6, 95 % < 5 µm	51		$FeSO_4.7H_2O$ RG	Hb reg	SC[1]	SP – 7 mg/kg	5 – 20	Pla et al., 1973 [95]
Iron, reduced – 7, 40 % < 10 µm; 1 % < 40 µm; electrolytic reduction	46		$FeSO_4.7H_2O$ RG	Hb reg	SC[1]	SP – 7 mg/kg	5 – 20	Pla et al., 1973 [95]

Iron, reduced – 8, 3 % < 10 μm; 21 % < 40 μm; hydrogen reduction	44		$FeSO_4.7H_2O$ RG	Hb reg	SC[1]	SP – 7 mg/kg	5 – 20	Pla et al., 1973 [95]
Iron, reduced – 9, 35 % < 32 μm; carbon monoxide reduction	42		$FeSO_4.7H_2O$ RG	Hb reg	SC[1]	SP – 7 mg/kg	5 – 20	Pla et al., 1973 [95]
Iron, reduced – 9, > 32 μm	41		$FeSO_4.7H_2O$ RG	Hb reg	SC[1]	SP – 7 mg/kg	5 – 20	Pla et al., 1973 [95]
Iron, reduced – 10, 77 % < 32 μm; electrolyte reduction	46		$FeSO_4.7H_2O$ RG	Hb reg	SC[1]	SP – 7 mg/kg	5 – 20	Pla et al., 1973 [95]
Iron, reduced – 10, > 32 μm	59		$FeSO_4.7H_2O$ RG	Hb reg	SC[1]	SP – 7 mg/kg	5 – 20	Pla et al., 1973 [95]
Iron, reduced – 11, 59 % < 32 μm; hydrogen reduction	56		$FeSO_4.7H_2O$ RG	Hb reg	SC[1]	SP – 7 mg/kg	5 – 20	Pla et al., 1973 [95]
Iron, reduced – 11, > 32 μm	49		$FeSO_4.7H_2O$ RG	Hb reg	SC[1]	SP – 7 mg/kg	5 – 20	Pla et al., 1973 [95]
Iron, reduced – 12, < 44 μm; electrolyte reduction	66		$FeSO_4.7H_2O$ RG	Hb reg	SC[1]	SP – 7 mg/kg	5 – 20	Pla et al., 1973 [95]
Iron, reduced – 13, RG; electrolyte reduction	59		$FeSO_4.7H_2O$ RG	Hb reg	SC[1]	SP – 7 mg/kg	5 – 20	Pla et al., 1973 [95]
Iron, reduced – 14, < 149 μm; hydrogen reduction	55		$FeSO_4.7H_2O$ RG	Hb reg	SC[1]	SP – 7 mg/kg	5 – 20	Pla et al., 1973 [95]
Limestone	52		$FeSO_4.7H_2O$ RG	Hb reg	SC	P – 5 mg/kg	5 – 20	Deming and Czarnecki-Maulden, 1989 [82]
Limestone, dolomitic	34		$FeSO_4.7H_2O$ RG	Hb reg	SC	P – 5 mg/kg	5 – 20	Deming and Czarnecki-Maulden, 1989 [82]
Meat and bone meal (575 mg/kg Fe AD)	48		$FeSO_4.7H_2O$ RG	Hb reg	SC	P	5 – 15	Chausow and Czarnecki-Maulden, 1988b [77]
Mono-dicalcium phosphate	67		$FeSO_4.7H_2O$ RG	Hb reg			7.5, 15.0, 22.5	Henry et al., 1992 [84]
Oat flour	21		$FeSO_4.7H_2O$ RG	Hb reg	SC[1]	SP – 7 mg/kg	5 – 20	Fritz et al., 1970 [81]

Oat hulls	69		Ferrous sulfate	Hb reg	SC	P – 10 mg/kg		Fly et al., 1998 [79]
Orchardgrass	69		Ferrous sulfate	Hb reg	SC	P – 10 mg/kg		Fly et al., 1998 [79]
Oystershell	10		$FeSO_4.7H_2O$ RG	Hb reg	SC	P – 5 mg/kg	5 – 20	Deming and Czarnecki-Maulden, 1989 [82]
Phosphate, defluorinated	48		$FeSO_4.7H_2O$ RG	Hb reg	SR	P – 4 mg/kg	7.5 – 22.5	Ammerman et al., 1993 [94]
Phosphate, defluorinated	44		$FeSO_4.7H_2O$ RG	Hb reg	SC	P – 5 mg/kg	5 – 20	Deming and Czarnecki-Maulden, 1989 [82]
Phosphate, dicalcium	55		$FeSO_4.7H_2O$ RG	Hb reg	SC	P – 5 mg/kg	5 – 20	Deming and Czarnecki-Maulden, 1989 [82]
Phosphate, monocalcium	61		$FeSO_4.7H_2O$ RG	Hb reg	SC	P – 5 mg/kg	5 – 20	Deming and Czarnecki-Maulden, 1989 [82]
Phosphate, monocalcium/dicalcium	66		$FeSO_4.7H_2O$ RG	Hb reg	SR	P – 4 mg/kg	7.5 – 22.5	Ammerman et al., 1993 [94]
Phosphate, soft rock	0		$FeSO_4.7H_2O$ RG	Hb reg	SC	P – 5 mg/kg	5 – 20	Deming and Czarnecki-Maulden, 1989 [82]
Pork liver (Barrow, Gilt)	46, 32		$FeSO_4.7H_2O$	Hb reg	SR	P - 47	18 - 20	Aoyagi et al., 1995 [96]
Poultry by-product meal (630 mg/kg Fe AD)	68		$FeSO_4.7H_2O$ RG	Hb reg	SC	P	5 – 15	Chausow and Czarnecki-Maulden, 1988b [77]
Psyllium husk, 5 % of diet	0			Hb reg	SR	P – 10 mg/kg		Fly et al., 1998 [79]
Rice, bran (120 mg/kg Fe AD)	77		$FeSO_4.7H_2O$	Hb reg	SC	P	10 – 20	Chausow and Czarnecki-Maulden, 1988b [77]
Sesame seed meal (120 mg/kg Fe AD)	96		$FeSO_4.7H_2O$	Hb reg	SC	P	10 – 20	Chausow and Czarnecki-

								Maulden, 1988b [77]
Sequestered Iron,<br>A,<br>B,<br>C	106*,<br>100,<br>104,<br>104		Ferrous sulfate	Hb reg	SC	N	5 – 20	McNaughton et al., 1974 [30]
Sodium iron pyrophosphate (14.5 % Fe)	30		$FeSO_4.7H_2O$ RG	Hb reg	SR	P < 7 mg/kg	10, 20, 40	Amine et al., 1972 [88]
Sodium iron pyrophosphate	14, 22*		$FeSO_4.7H_2O$ RG	Hb reg	SR	P < 7 mg/kg	5 – 40	Amine et al., 1972 [88]
Sodium iron pyrophosphate	2 (2 – 23)[2]		$FeSO_4.7H_2O$ RG	Hb reg	SC[1]	SP – 7 mg/kg	5 – 20	Fritz et al., 1970 [81]
Sodium iron pyrophosphate	13, 8*		$FeSO_4.7H_2O$ RG	Hb reg	SC[1]	SP – 7 mg/kg	5 – 20	Fritz et al., 1970 [81]
Sodium iron pyrophosphate (14.5 % Fe)	13, 13*		$FeSO_4.7H_2O$ RG	Hb reg	SC[1]	SP – 7 mg/kg	5 – 20	Pla and Fritz, 1971 [89]
Sodium iron pyrophosphate	12		$FeSO_4.7H_2O$ RG	Hb reg	SC[1]	SP	5 – 20	Pla and Fritz, 1970 [80]
Sodium iron pyrophosphate	5*		Ferrous sulfate	Hb reg				Pennell et al., 1976 [90]
Soybeans (heated to 140 °C)	90		$FeSO_4.7H_2O$ RG	Hb reg	SC[1]	SP – 7 mg/kg	5 – 20	Pla et al., 1973 [95]
Soybeans (heated to 170 °C)	77		$FeSO_4.7H_2O$ RG	Hb reg	SC[1]	SP – 7 mg/kg	5 – 20	Pla et al., 1973 [95]
Soybean meal (345 mg/kg Fe AD)	45		$FeSO_4.7H_2O$ RG	Hb reg	SC	P	10 – 20	Chausow and Czarnecki-Maulden, 1988b [77]
Soybean meal	39							Biehl et al., 1997 [97]
Soybean hulls	94		Ferrous sulfate	Hb reg	SC	P – 10 mg/kg		Fly et al., 1998 [79]
Soybean protein isolate	97 (70 – 125)[2]		$FeSO_4.7H_2O$ RG	Hb reg	SC[1]	P – 7 mg/kg	5 – 20	Fritz et al., 1970 [81]
Tomato pomace	82		Ferrous sulfate	Hb reg	SC	P – 10 mg/kg		Fly et al., 1998 [79]
Wheat germ	53		$FeSO_4.7H_2O$ RG	Hb reg	SC[1]	P – 7 mg/kg	5 – 20	Fritz et al., 1970 [81]

Wheat germ meal	54		$FeSO_4.7H_2O$ RG	Hb reg	SC[1]	SP	5 – 20	Pla and Fritz, 1970 [80]
Zn-$FeSO_4.H_2O$ (20.2 % Zn, 14.2 % Fe)	96*[3] 93		Ferrous sulfate	Hb reg	SR	SP – 20 mg/kg	10, 20	Boling et al., 1998 [83]

AD = air – dried; Hb = hemoglobin; reg = regeneration; SC = standard curve; SR = slope ratio; P = purified; SP = semi-purified; N = natural; Liv = liver; Kid = kidney; RG = reagent grade; FG = feed grade; Met = methionine; Prot = proteinate; SDH = succinate dehydrogenase; mRNA = messenger ribonucleic acid; (W) = weak; (M) = moderate; (ES) = exceptionally strong; MR = mean ratio; TP = three-point; EDTA = ethylenediaminetetraacetate

[1] RV = 100 x (mg/kg Fe from standard / mg/kg from test source to give equal curative effect).

[2] Values in brackets represent author values where more than one availability test was made and reflects variation both between samples and between repeated determinations on the same sample

[3] Recalculated using ferrous sulfate monohydrate as a reference

[4] Authors state proper conclusion is that this value is equivalent rather than greater than the Fe present in the feed-grade ferrous sulfate after additional analyses

[5] Fe Prot. (M) in the Zhang et al. study is the same as that used in the Ma et al. study but its bioavailability value relative to $FeSO_4.7H_2O$ is about 40 % lower in the Ma et al. study (116 % vs. 154 %) with authors suggesting the difference may be due to the reduced feed intake and inhibited growth and Fe utilisation of broilers fed a purified casein-dextrose diet in the Ma study.

Table S7. Sheep

**Source**	**RV, %**	**Standard**	**Response Criterion**	**Method Calculation**	**Type diet**	**Added level, mg/kg**	**Reference**
Ferrous carbonate A	55 (40, 112, 13)	$FeSO_4.7H_2O$ FG	Liv, Kid, spl	SR	N – 90 mg/kg	600	Van Ravenswaay et al., 2001 [98]
Ferrous carbonate B	0 (0, 0, 1)	$FeSO_4.7H_2O$ FG	Liv, Kid, spl	SR	N – 90 mg/kg	600	Van Ravenswaay et al., 2001 [98]
Ferrous carbonate C	20 (13, 18, 29)	$FeSO_4.7H_2O$ FG	Liv, Kid, spl	SR	N – 90 mg/kg	600	Van Ravenswaay et al., 2001 [98]

N = natural; Liv = liver; Kid = kidney; Spl = spleen; FG = feed grade; SR = slope ratio

Table S8. Swine

**Source**	**RV, %**	**Standard**	**Response Criterion**	**Method Calculation**	**Type diet**	**Added level, mg/kg**	**Reference**
Ferric ammonium citrate	102, 102*	Ferrous sulfate	Hb	TP	P – 39 mg/kg	88	Harmon et al., 1967 [99]
Ferric choline citrate	144, 118*	$FeSO_4.7H_2O$	Hb reg	SC	N	50, 100	Miller et al., 1981 [100]

Ferric citrate	89	$FeSO_4.7H_2O$	Hb	MR	N	176 mg/day	Ullrey et al., 1973 [101]
Ferric citrate	192	$FeSO_4.7H_2O$	Liv Fe	MR	N	176 mg/day	Ullrey et al., 1973 [101]
Ferric citrate	190	$FeSO_4.7H_2O$	Hb	MR	N	176 mg/day	Ullrey et al., 1973 [101]
Ferric citrate	125, 114*	$FeSO_4.7H_2O$	Liv Fe	MR	N	176 mg/day 100 mg/kg *	Ullrey et al., 1973 [101]
Ferric copper, cobalt choline citrate	144, 114*	$FeSO_4.7H_2O$	Hb reg	SC	N	50, 100	Miller et al., 1981 [100]
Ferric oxide RG Fe_2O_3	12	$FeSO_4.7H_2O$ RG	Hb	TP	SP – 15 mg/kg	35 – 65	Pickett et al., 1961 [102]
Ferric polyphosphate	84, 91*	$FeSO_4.7H_2O$	Hb	TP	P – 8 mg/kg	64 – 69	Anderson et al., 1974 [103]
Ferrous carbonate (40 % Fe)	22, 98*	$FeSO_4.7H_2O$	Hb	TP	N		Poitevint, 1979 [92]
Ferrous carbonate FG-A	97	Ferrous sulfate RG	Hb reg	MR	N – 61 mg/kg	40	Ammerman et al., 1974 [104]
Ferrous carbonate FG-B	87, 81*	Ferrous sulfate RG	Hb reg	MR	N – 61 mg/kg	40	Ammerman et al., 1974 [104]
Ferrous carbonate FG-C	101	Ferrous sulfate RG	Hb reg	MR	N – 61 mg/kg	40	Ammerman et al., 1974 [104]
Ferrous carbonate FG-A – Exp. 1	74	Ferrous sulfate RG	Hb reg	TP	N – 61 mg/kg	40	Ammerman et al., 1974 [104]
Ferrous carbonate FG-B – Exp. 1	40	Ferrous sulfate RG	Hb reg	TP	N – 61 mg/kg	40	Ammerman et al., 1974 [104]
Ferrous carbonate FG-C – Exp. 1	34	Ferrous sulfate RG	Hb reg	TP	N – 61 mg/kg	40	Ammerman et al., 1974 [104]
Ferrous carbonate FG-A – Exp. 2	45, 95, 55, 66[1]	Ferrous sulfate RG	Hb reg	MR	N – 23 mg/kg	80	Ammerman et al., 1974 [104]
Ferrous carbonate FG-B – Exp. 2	30, 97, 40, 56[1]	Ferrous sulfate RG	Hb reg	MR	N – 23 mg/kg	80	Ammerman et al., 1974 [104]
Ferrous carbonate FG-C – Exp. 2	20, 85, 25, 42[1]	Ferrous sulfate RG	Hb reg	MR	N – 23 mg/kg	80	Ammerman et al., 1974 [104]
Ferrous carbonate	8, 74*	Ferrous sulfate	Hb reg	TP	P – 12 – 34 mg/kg	36 – 51	Harmon et al., 1969 [105]
Ferrous carbonate – 1	13	$FeSO_4.7H_2O$ RG	Hb	TP	SP – 15 mg/kg	35 – 65	Pickett et al., 1961 [102]
Ferrous carbonate – 2	16	$FeSO_4.7H_2O$ RG	Hb	TP	SP – 15 mg/kg	35 – 65	Pickett et al., 1961 [102]
Ferrous sulfate ($FeSO_4.H_2O$)	87, 99*	$FeSO_4.7H_2O$	Hb reg	SR	N – 56 mg/kg	50, 100	Miller, 1978 [106]

Ferrous sulfate ($FeSO_4.H_2O$)	101*	$FeSO_4.7H_2O$	Hb	TP	N	320 – 360	Poitevint, 1979 [92]
Fe methionine	103	$FeSO_4.7H_2O$	Erythrocyte counts				Kuznetsov, 1987 [107]
Iron EDTA, disodium	90, 91*	$FeSO_4.7H_2O$	Hb	TP	P – 8 mg/kg	64 – 68	Anderson et al., 1974 [103]
Iron – methionine	183	Ferrous sulfate	Hb	TP		200 mg	Spears et al., 1992 [108]
Iron – methionine	81	Ferrous sulfate	Growth				Lewis et al., 1996 [109]
Iron – methionine	68	Ferrous sulfate	Hb				Lewis et al., 1996 [109]
Iron – proteinate	123	$FeSO_4.7H_2O$	Hb	TP	N	500	Brady et al., 1978 [110]
Iron, reduced catalytic	27, 78*	$FeSO_4.7H_2O$	Hb	TP	P – 8 mg/kg	64 – 69	Anderson et al., 1974 [103]
Iron, reduced electrolytic	63, 86*	$FeSO_4.7H_2O$	Hb	TP	P – 8 mg/kg	64 – 69	Anderson et al., 1974 [103]
Humic substances (8,700 mg/kg Fe)	71	$FeSO_4$	RBC	SR	N – 27 mg/kg	70	Kim et al., 2004 [111]
Phosphate, defluorinated	35	Ferrous sulfate	Hb	TP	SP – 20 mg/kg	70 – 125	Kornegay, 1972 [112]
Phosphate, defluorinated	73	Ferrous sulfate	Liv Fe	TP	SP – 20 mg/kg	70 – 125	Kornegay, 1972 [112]
Sodium iron pyrophosphate	29, 81*	$FeSO_4.7H_2O$	Spl Fe	TP	P – 8 mg/kg	64 – 69	Anderson et al., 1974 [103]

Hb = hemoglobin; reg = regeneration; TP = three-point; SC = standard curve; MR = mean ratio; SR = slope ratio; P = purified; SP = semi-purified; N = natural; Liv = liver; Kid = kidney; Spl = spleen; RG = reagent grade; FG = feed grade; EDTA = ethylenediaminetetraacetate; RBC = red blood cells

[1] Variation in the Ammerman et al. values show how data from one experiment can provide bioavailability estimates which vary widely depending on the calculations used (Van Ravenswaay et al., 2001)

MANGANESE

Individual species tables for relative bioavailability of supplemental manganese sources

Table S9. Poultry

**Source**	**RV, %**	**Q_f**	**Standard**	**Response Criterion**	**Method Calculation**	**Type diet**	**Added level, mg/kg**	**Reference**

Hausmannite (Mn_3O_4)	97		$MnSO_4.2H_2O$	Growth	MR	N – 11 mg/kg	20, 30	Schaible et al., 1938 [113]
$KmnO_4$ RG	94		$MnSO_4.4H_2O$ RG	Growth	MR	N – 10 mg/kg	50	Gallup and Norris, 1939 [114]
Manganese carbonate ($MnCO_3$)	101		$MnSO_4.2H_2O$	Growth	MR	N – 11 mg/kg	20, 30	Schaible et al., 1938 [113]
Manganese dioxide (MnO_2)	106		$MnSO_4.2H_2O$	Growth	MR	N – 11 mg/kg	20, 30	Schaible et al., 1938 [113]
Manganese hematite	104		$MnSO_4.2H_2O$	Growth	MR	N – 11 mg/kg	20, 30	Schaible et al., 1938 [113]
Manganese – methionine	111		MnO	Bone Mn	MR	N	30, 60, 200	Scheideler, 1991 [115]
Manganese – methionine	101		MnO	Liv Mn	MR	N	30, 60, 200	Scheideler, 1991 [115]
Manganese – methionine (16 % Mn)	130		MnO FG	Bone Mn	SR	P – 1.4 mg/kg	13 – 1285	Fly et al., 1989 [116]
Manganese – methionine	174		MnO FG	Bone Mn	SR	P + 10 % N	13 – 1285	Fly et al., 1989 [116]
Manganese – methionine (16 % Mn)	108, 101*		$MnSO_4.H_2O$	Bone Mn	SR	N – 93 mg/kg	700, 1400, 2100	Henry et al., 1989 [117]
Manganese – methionine	132		$MnSO_4.H_2O$	Kid Mn	SR	N – 93 mg/kg	700, 1400, 2100	Henry et al., 1989 [117]
Manganese monoxide RG (MnO) (76.7 % Mn)	60, 66, 81*		$MnSO_4.H_2O$	Bone Mn	SR	N – 116 mg/kg	1000, 2000, 4000	Black et al., 1984 [118]
Manganese oxide FG ($MnO + Mn_3O_4$)	103		$MnSO_4.H_2O$ RG	Bone Mn	MR	P – 4 mg/kg	10, 20	Watson et al., 1970 [119]
Manganese oxide FG ($MnO + Mn_3O_4$)	98		$MnSO_4.H_2O$ RG	PI[1]	MR	P – 4 mg/kg	10, 20	Watson et al., 1970 [119]
Manganese oxide FG ($MnO + Mn_3O_4$)	70		$MnSO_4.H_2O$	Bone Mn	SR	N – 82 mg/kg	1000, 2000, 3000	Wong-Valle et al., 1989a [120]
Manganese oxide FG ($MnO + Mn_3O_4$)	53		$MnSO_4.H_2O$	Kid Mn	SR	N – 82 mg/kg	1000, 2000, 3000	Wong-Valle et al., 1989a [120]
Manganese oxide FG ($MnO + Mn_2SiO_4$) (58.0 % Mn)	96		$MnSO_4.H_2O$ RG	Bone Mn	MR	P – 5 mg/kg	10	Watson et al., 1971 [121]

Manganese oxide FG ($MnO + Mn_2SiO_4$)	78		$MnSO_4.H_2O$ RG	PI[2]	MR	P – 5 mg/kg	10	Watson et al., 1971 [121]
Manganese oxide FG [(Fe, $Mn)_2O_3$ + Mn_3O_4 + MnO + MnO_2] (57.3 % Mn)	86		$MnSO_4.H_2O$ RG	Bone Mn	MR	P – 5 mg/kg	10	Watson et al., 1971 [121]
Manganese oxide FG [(Fe, $Mn)_2O_3$ + Mn_3O_4 + MnO + MnO_2]	60		$MnSO_4.H_2O$ RG	PI[2]	MR	P – 5 mg/kg	10	Watson et al., 1971 [121]
Manganese – proteinate (10 % Mn)	86, 101*		$MnSO_4.H_2O$	Bone Mn	MR	P – 1.4 mg/kg	1000	Baker and Halpin, 1987 [122]
Manganese – proteinate	118		$MnSO_4.H_2O$	Bile Mn	MR	P – 1.4 mg/kg	1000	Baker and Halpin, 1987 [122]
Manganese – proteinate	114		$MnSO_4.H_2O$	Bone Mn	MR	P + 10 % wheat bran	1000	Baker and Halpin, 1987 [122]
Manganese – proteinate	122		$MnSO_4.H_2O$	Bile Mn	MR	P + 10 % wheat bran	1000	Baker and Halpin, 1987 [122]
Manganese proteinate (15 % Mn)	117*		$MnSO_4.H_2O$	Bone Mn	SR	N – 26 mg/kg	1000, 2000, 3000	Smith et al., 1995 [123]
Manganese proteinate (21 d) (15 % Mn)	120		$MnSO_4.H_2O$	Bone Mn	SR	N – 26 mg/kg	1000, 2000, 3000	Smith et al., 1995 [123]
Manganese proteinate (49 d, TN) (15 % Mn)	125		$MnSO_4.H_2O$	Bone Mn	SR	N – 26 mg/kg	1000, 2000, 3000	Smith et al., 1995 [123]
Manganese proteinate (49 d, HD) (15 % Mn)	145		$MnSO_4.H_2O$	Bone Mn	SR	N – 26 mg/kg	1000, 2000, 3000	Smith et al., 1995 [123]
Manganese proteinate	86	1.45	$MnSO_4.H_2O$	Heart	SR	N – 15	60, 120, 180	Wang et al., 2012 [124]
Manganese proteinate	95	1.45	$MnSO_4.H_2O$	MnSOD	SR	N – 15	60, 120, 180	Wang et al., 2012 [124]
Manganese proteinate	103	1.45	$MnSO_4.H_2O$	MnSOD mRNA	SR	N – 15	60, 120, 180	Wang et al., 2012 [124]
Manganese proteinate (18.3 % Mn)	111		$MnSO_4.H_2O$	Bone strength	SR	N – 13 mg/kg	35, 70, 105, 140	Saldanha et al., 2020 [125]

Manganese proteinate (18.3 % Mn)	128		$MnSO_4.H_2O$	Liv Mn	SR	N – 13 mg/kg	35, 70, 105, 140	Saldanha et al., 2020 [125]
Manganese proteinate (18.3 % Mn)	105		$MnSO_4.H_2O$	Bone Mn	SR	N – 13 mg/kg	35, 70, 105, 140	Saldanha et al., 2020 [125]
Manganite Mn_2O_3	93		$MnSO_4.2H_2O$	Growth	MR	N – 11 mg/kg	20, 30	Schaible et al., 1938 [113]
Manganomanganic oxide FG (Mn_3O_4) (36.1 % Mn)	101		$MnSO_4.H_2O$ RG	Bone Mn	MR	P – 4 mg/kg	10, 20	Watson et al., 1970 [119]
Mn_3O_4 FG	88		$MnSO_4.H_2O$ RG	PI[2]	MR	P – 4 mg/kg	10, 20	Watson et al., 1970 [119]
Manganous chloride ($MnCl_2.4H_2O$)	101		$MnSO_4.2H_2O$	Growth	MR	N – 11 mg/kg	20, 30	Schaible et al., 1938 [113]
Mn amino acid B	96[3]	45.3	$MnSO_4.H_2O$ RG	Bone Mn	SR	N – 23 mg/kg	60, 120, 180	Li et al., 2004 [126]
Mn amino acid B	127[3]	45.3	$MnSO_4.H_2O$ RG	Heart	SR	N – 23 mg/kg	60, 120, 180	Li et al., 2004 [126]
Mn amino acid B	133[3]	45.3	$MnSO_4.H_2O$ RG	MnSOD mRNA (heart)	SR	N – 23 mg/kg	60, 120, 180	Li et al., 2004 [126]
Mn amino acid B	132		$MnSO_4.H_2O$ RG	MnSOD mRNA (heart)	SR		60, 120, 180	Luo et al., 2004 [127]
Mn amino acid B (6.48 % Mn)	102[3]	45.3	$MnSO_4.H_2O$ RG	Bone Mn	SR	N – 20 mg/kg + high Ca (18.5 g/kg)	60, 80, 120	Li et al., 2005 [128]
Mn amino acid B (6.48 % Mn)	137[3]	45.3	$MnSO_4.H_2O$ RG	Heart	SR	N – 20 mg/kg + high Ca (18.5 g/kg)	60, 80, 120	Li et al., 2005 [128]
Mn amino acid B (6.48 % Mn)	108[3]	45.3	$MnSO_4.H_2O$ RG	MnSOD activity (heart)	SR	N – 20 mg/kg + high Ca (18.5 g/kg)	60, 80, 120	Li et al., 2005 [128]

Mn amino acid B (6.48 % Mn)	145[3]	45.3	$MnSO_4.H_2O$ RG	MnSOD mRNA (heart)	SR	N – 20 mg/kg + high Ca (18.5 g/kg)	60, 80, 120	Li et al., 2005 [128]
Mn amino acid C	93[3]	115.4	$MnSO_4.H_2O$ RG	Bone Mn	SR	N – 23 mg/kg	60, 120, 180	Li et al., 2004 [126]
Mn amino acid C	114[3]	115.4	$MnSO_4.H_2O$ RG	Heart	SR	N – 23 mg/kg	60, 120, 180	Li et al., 2004 [126]
Mn amino acid C	113[3]	115.4	$MnSO_4.H_2O$ RG	MnSOD mRNA (heart)	SR	N – 23 mg/kg	60, 120, 180	Li et al., 2004 [126]
Mn amino acid C	113		$MnSO_4.H_2O$ RG	MnSOD mRNA (heart)	SR		60, 120, 180	Luo et al., 2004 [127]
Mn amino acid C (7.86 % Mn)	102[3]	115.4	$MnSO_4.H_2O$ RG	Bone Mn	SR	N – 20 mg/kg + high Ca (18.5 g/kg	60, 120, 180	Li et al., 2005 [128]
Mn amino acid C (7.86 % Mn)	135[3]	115.4	$MnSO_4.H_2O$ RG	Heart	SR	N – 20 mg/kg + high Ca (18.5 g/kg	60, 120, 180	Li et al., 2005 [128]
Mn amino acid C (7.86 % Mn)	120[3]	115.4	$MnSO_4.H_2O$ RG	MnSOD activity (heart)	SR	N – 20 mg/kg + high Ca (18.5 g/kg	60, 120, 180	Li et al., 2005 [128]
Mn amino acid C (7.86 % Mn)	148[3]	115.4	$MnSO_4.H_2O$ RG	MnSOD mRNA (heart)	SR	N – 20 mg/kg + high Ca (18.5 g/kg	60, 120, 180	Li et al., 2005 [128]
Mn amino acid chelate	84		$MnSO_4.H_2O$ RG	Bone Mn	SR	N – 118 mg/kg	500, 1000, 1500	Miles et al., 2003 [28]
Mn chelate of 2-hydroxy-4(methylthio) butanoic acid (HMTBa)	116		$MnSO_4.5H_2O$ RG	Bone Mn	SR	N	100, 200, 400, 600, 800	Yan and Waldroup, 2006 [129]

Mn chelate of 2-hydroxy-4(methylthio) butanoic acid (HMTBa)	154		MnO RG	Bone Mn	SR	N	100, 200, 400, 600, 800	Yan and Waldroup, 2006 [129]
$MnCl_2.4H_2O$ RG	93		$MnSO_4.4H_2O$ RG	Growth	MR	N – 10 mg/kg	50	Gallup and Norris, 1939 [114]
$MnCl_2.4H_2O$ RG	102, 97*		$MnSO_4.H_2O$ RG	Bile Mn	SR	N – 168 mg/kg	3000, 4000	Southern and Baker, 1983 [130]
$MnCO_3$ RG	94		$MnSO_4.4H_2O$ RG	Growth	MR	N – 10 mg/kg	50	Gallup and Norris, 1939 [114]
$MnCO_3$ RG (45 % Mn)	93		$MnSO_4.H_2O$ RG	Bone Mn	MR		10	Watson et al., 1971 [121]
$MnCO_3$ RG	98		$MnSO_4.H_2O$ RG	PI[2]	MR		10	Watson et al., 1971 [121]
$MnCO_3$ RG	77		$MnSO_4.H_2O$ RG	Bile Mn	SR	N – 168 mg/kg	3000, 4000	Southern and Baker, 1983 [130]
$MnCO_3$ RG (47 % Mn)	32, 66*		$MnSO_4.H_2O$	Bone Mn	SR	N – 116 mg/kg	1000, 2000, 4000	Black et al., 1984 [118]
$MnCO_3$ RG	36		$MnSO_4.H_2O$	Liv Mn	SR	N – 116 mg/kg	1000, 2000, 4000	Black et al., 1984 [118]
Mn methionine E	99[3]	3.2	$MnSO_4.H_2O$ RG	MnSOD mRNA (heart)	SR	P – 23 mg/kg	60, 120, 180	Li et al., 2004 [126]
Mn methionine E	95[3]	3.2	$MnSO_4.H_2O$ RG	Bone Mn	SR	P – 23 mg/kg	60, 120, 180	Li et al., 2004 [126]
Mn methionine E	110[3]	3.2	$MnSO_4.H_2O$ RG	Heart	SR	P – 23 mg/kg	60, 120, 180	Li et al., 2004 [126]
Mn methionine E	99		$MnSO_4.H_2O$ RG	MnSOD mRNA (heart)	SR		60, 120, 180	Luo et al., 2004 [127]
Mn methionine E (8.27 % Mn)	104[3]	3.2	$MnSO_4.H_2O$ RG	Bone Mn	SR	N – 20 mg/kg + high Ca (18.5 g/kg	60, 120, 180	Li et al., 2005 [128]

Mn methionine E (8.27 % Mn)	126[3]	3.2	$MnSO_4.H_2O$ RG	Heart Mn	SR	N – 20 mg/kg + high Ca (18.5 g/kg	60, 120, 180	Li et al., 2005 [128]
Mn methionine E (8.27 % Mn)	108[3]	3.2	$MnSO_4.H_2O$ RG	MnSOD activity (heart)	SR	N – 20 mg/kg + high Ca (18.5 g/kg	60, 120, 180	Li et al., 2005 [128]
Mn methionine E (8.27 % Mn)	112[3]		$MnSO_4.H_2O$ RG	MnSOD mRNA (heart)	SR	N – 20 mg/kg + high Ca (18.5 g/kg	60, 120, 180	Li et al., 2005 [128]
MnO RG	77, 79		$MnSO_4.H_2O$	Liv Mn	SR	N – 116 mg/kg	1000, 2000, 4000	Black et al., 1984 [118]
MnO	75		$MnSO_4.5H_2O$ RG	Bone Mn	SR	N	100, 200, 400, 600, 800	Yan and Waldroup, 2006 [129]
MnO RG	81, 70*		$MnSO_4.H_2O$	Bone Mn	SR	N – 35 mg/kg	40, 80, 120	Henry et al., 1986 [131]
MnO RG	46		$MnSO_4.H_2O$	Kid Mn	SR	N – 35 mg/kg	40, 80, 120	Henry et al., 1986 [131]
MnO RG	70		$MnSO_4.H_2O$	Liv Mn	SR	N – 35 mg/kg	40, 80, 120	Henry et al., 1986 [131]
MnO RG (73.0 % Mn)	82		$MnSO_4.H_2O$	Bone Mn	SR	N – 82 mg/kg	1000, 2000, 3000	Wong-Valle et al., 1989a [120]
MnO RG	86		$MnSO_4.H_2O$	Kid Mn	SR	N – 82 mg/kg	1000, 2000, 3000	Wong-Valle et al., 1989a [120]
MnO RG (77.2 % Mn)	96, 94*		$MnSO_4.H_2O$	Bone Mn	SR	N – 93 mg/kg	700, 1400, 2100	Henry et al., 1989 [117]
MnO RG	86		$MnSO_4.H_2O$	Kid Mn	SR	N – 93 mg/kg	700, 1400, 2100	Henry et al., 1989 [117]
MnO FG (14.8 % Mn)	82		$MnSO_4.H_2O$ RG	Bone Mn	MR	P – 5 mg/kg	10	Watson et al., 1971 [121]
MnO FG	73		$MnSO_4.H_2O$ RG	PI[2]	MR	P – 5 mg/kg	10	Watson et al., 1971 [121]
MnO FG (61.0 % Mn)	95		$MnSO_4.H_2O$ RG	Bone Mn	MR	P – 5 mg/kg	10	Watson et al., 1971 [121]
MnO FG	83		$MnSO_4.H_2O$ RG	PI[2]	MR	P – 5 mg/kg	10	Watson et al., 1971 [121]

MnO FG (64.2 % Mn)	93, 91*		$MnSO_4.H_2O$	Bone Mn	SR	N – 82 mg/kg	1000, 2000, 3000	Wong-Valle et al., 1989a [120]
MnO FG	68		$MnSO_4.H_2O$	Kid Mn	SR	N – 82 mg/kg	1000, 2000, 3000	Wong-Valle et al., 1989a [120]
MnO FG (45.6 % Mn)	75		$MnSO_4.H_2O$	Bone Mn	SR	N – 82 mg/kg	1000, 2000, 3000	Wong-Valle et al., 1989a [120]
MnO FG	52		$MnSO_4.H_2O$	Kid Mn	SR	N – 82 mg/kg	1000, 2000, 3000	Wong-Valle et al., 1989a [120]
MnO FG (46 % Mn)	83		$MnSO_4.H_2O$ RG	Bone Mn	SR	N – 22 mg/kg	50, 100, 150, 200	Luo, 1989 [132]
MnO FG	83		$MnSO_4.H_2O$ RG	Toe Mn	SR	N – 22 mg/kg	50, 100, 150, 200	Luo, 1989 [132]
MnO (15 % Mn)	99*		$MnSO_4.H_2O$	Bone Mn	SR	N – 26 mg/kg	1000, 2000, 3000	Smith et al., 1995 [123]
MnO (21 d) (15 % Mn)	91		$MnSO_4.H_2O$	Bone Mn	SR	N – 26 mg/kg	1000, 2000, 3000	Smith et al., 1995 [123]
MnO (49 d, TN) (15 % Mn)	83		$MnSO_4.H_2O$	Bone Mn	SR	N – 26 mg/kg	1000, 2000, 3000	Smith et al., 1995 [123]
MnO (49 d, HD) (15 % Mn)	82		$MnSO_4.H_2O$	Bone Mn	SR	N – 26 mg/kg	1000, 2000, 3000	Smith et al., 1995 [123]
MnO	74*		$MnSO_4.H_2O$	Bone Mn			50	Korol et al., 1996 [133]
$MnO.Mn_2O_3$	68*		$MnSO_4.H_2O$	Bone Mn			50	Korol et al., 1996 [133]
MnO_2 RG	95		$MnSO_4.4H_2O$ RG	Growth	MR	N – 10 mg/kg	50	Gallup and Norris, 1939 [114]
MnO_2 RG	29		$MnSO_4.H_2O$ RG	Bile Mn	SR	N – 168 mg/kg	3000, 4000	Southern and Baker, 1983 [130]
MnO_2 RG (56.5 % Mn)	34, 31*		MnO RG (77 % Mn)	Bone Mn	SR	N – 102 mg/kg	1000, 2000, 3000	Henry et al., 1987 [134]
MnO_2 RG	46		MnO RG	Kid Mn	SR	N – 102 mg/kg	1000, 2000, 3000	Henry et al., 1987 [134]
MnO_2 FG (36 % Mn)	37		MnO RG	Bone Mn	SR	N – 102 mg/kg	1000, 2000, 3000	Henry et al., 1987 [134]
MnO_2 FG	44		MnO RG	Kid Mn	SR	N – 102 mg/kg	1000, 2000, 3000	Henry et al., 1987 [134]

Mn Propionate (high Ca and P diet)	139		Mn sulfate FG	Bone Mn	SR	N – 30 mg/kg	20, 100, 500	Brooks et al., 2012 [135]
Mn Proteinate	163		$MnSO_4$.	Liv Mn	SR			Ao et al., 2008 [136]
Mn Proteinate	120		$MnSO_4$.	Bone Mn	SR			Ao et al., 2008 [136]
Mn Proteinate	133		$MnSO_4$	Kid Mn	SR			Ao et al., 2008 [136]
Potassium permanganate ($KmnO_4$)	109		$MnSO_4.2H_2O$	Growth	MR	N – 11 mg/kg	20, 30	Schaible et al., 1938 [113]
Psilomelane	87		$MnSO_4.2H_2O$	Growth	MR	N – 11 mg/kg	20, 30	Schaible et al., 1938 [113]
Pyrolusite (MnO_2)	81		$MnSO_4.2H_2O$	Growth	MR	N – 11 mg/kg	20, 30	Schaible et al., 1938 [113]
Rhodochrosite ($MnCO_3$)	64		$MnSO_4.2H_2O$	Growth	MR	N – 11 mg/kg	20, 30	Schaible et al., 1938 [113]
Rhodochrosite FG (24 % Mn)	80		$MnSO_4.H_2O$ RG	Bone Mn	MR	P – 5 mg/kg	10	Watson et al., 1971 [121]
Rhodochrosite FG	39		$MnSO_4.H_2O$ RG	PI[2]	MR	P – 5 mg/kg	10	Watson et al., 1971 [121]
Rhodonite	85		$MnSO_4.2H_2O$	Growth	MR	N – 11 mg/kg	20, 30	Schaible et al., 1938 [113]

MR = mean ratio; SR = slope ratio; P = purified; N = natural; Liv = liver; Kid = kidney; PI = perosis index; RG = reagent grade; FG = feed grade; MnSOD = manganese superoxide dismutase activity; mRNA = messenger ribonucleic acid

[1] Value for standard source was ≤ that for unsupplemented control group

[2] Authors provided a leg score of 0 = normal to 4 = swelling and marked slipping of tendon. Relative value calculated as: (Leg score test source – 4) / (leg score standard source – 4) x 100.

[3] Li et al. (2004 & 2005) – 2005 results in high Ca diet show higher RBV implying organic Mn sources with moderate or strong chelation strength offer partial or complete resistance to interference from high dietary calcium during digestion.

Table S10. Sheep

**Source**	**RV, %**	**Standard**	**Response Criterion**	**Method Calculation**	**Type diet**	**Added level, mg/kg**	**Reference**
Manganese carbonate RG ($MnCO_3$)	93, 74*	MnO FG	Bone Mn	SR	N – 31 mg/kg	500 – 8000	Black et al., 1985 [137]
Manganese dioxide RG (MnO_2)	39, 67*	$MnSO_4.H_2O$	Bone Mn	SR	N – 38 mg/kg	1500, 3000	Wong-Valle et al., 1989b [138]

Manganese – methionine (15.7 % Mn)	157, 113*, 164	$MnSO_4.H_2O$ RG	Bone Mn	SR	N – 34 mg/kg	900, 1800, 2700	Henry et al., 1992 [139]
Manganese – methionine	102, 93, 116	$MnSO_4.H_2O$ RG	Kid Mn	SR	N – 34 mg/kg	900, 1800, 2700	Henry et al., 1992 [139]
Manganese – methionine	132	$MnSO_4.H_2O$ RG	Bone Mn	SR	N – 32 mg/kg	900 – 2700	Henry et al., 1992 [139]
$MnCO_3$ RG	46	MnO FG	Kid Mn	SR	N – 31 mg/kg	500 – 8000	Black et al., 1985 [137]
$MnCO_3$ RG	23, 61*	$MnSO_4.H_2O$	Bone Mn	SR	N – 38 mg/kg	1500, 3000	Wong-Valle et al., 1989b [138]
$MnCO_3$ RG	20	$MnSO_4.H_2O$	Kid Mn	SR	N – 38 mg/kg	1500, 3000	Wong-Valle et al., 1989b [138]
$MnCO_3$ RG	40	$MnSO_4.H_2O$	Liv Mn	SR	N – 38 mg/kg	1500, 3000	Wong-Valle et al., 1989b [138]
MnO RG (73.0 % Mn)	57, 80*	$MnSO_4.H_2O$	Bone Mn	SR	N – 38 mg/kg	1500, 3000	Wong-Valle et al., 1989b [138]
MnO RG	55	$MnSO_4.H_2O$	Kid Mn	SR	N – 38 mg/kg	1500, 3000	Wong-Valle et al., 1989b [138]
MnO RG	61	$MnSO_4.H_2O$	Liv Mn	SR	N – 38 mg/kg	1500, 3000	Wong-Valle et al., 1989b [138]
MnO FG (64.2 % Mn)	70, 91*, 67	$MnSO_4.H_2O$ RG	Bone Mn	SR	N – 32 mg/kg	900 – 2700	Henry et al., 1992 [139]
MnO FG	75, 55	$MnSO_4.H_2O$ RG	Kid Mn	SR	N – 32 mg/kg	900 – 2700	Henry et al., 1992 [139]
MnO FG (58.1 % Mn)	50, 46	$MnSO_4.H_2O$ RG	Bone Mn	SR	N – 32 mg/kg	900 – 2700	Henry et al., 1992 [139]
MnO FG	59, 31	$MnSO_4.H_2O$ RG	Kid Mn	SR	N – 32 mg/kg	900 – 2700	Henry et al., 1992 [139]
MnO_2 RG	25	$MnSO_4.H_2O$	Kid Mn	SR	N – 38 mg/kg	1500, 3000	Wong-Valle et al., 1989b [138]
MnO_2 RG	35	$MnSO_4.H_2O$	Liv Mn	SR	N – 38 mg/kg	1500, 3000	Wong-Valle et al., 1989b [138]

SR = slope ratio; N = natural; Liv = liver; Kid = kidney; RG = reagent grade; FG = feed grade

Table S11. Swine

**Source**	**RV, %**	**Standard**	**Response Criterion**	**Method Calculation**	**Type diet**	**Added level, mg/kg**	**Reference**

Manganese carbonate	95*	$MnSO_4.H_2O$	Mn Abs			10	Kayongo-Male et al., 1980 [140]
Manganese oxide	96*	$MnSO_4.H_2O$	Mn Abs			10	Kayongo-Male et al., 1980 [140]

Abs = absorption

ZINC

Individual species tables for relative bioavailability of supplemental zinc sources

Table S12. Cattle

Source	RV, %	Standard	Response Criterion	Method Calculation	Type diet	Added level, mg/kg	Reference
Corn forage	100	$^{65}Zn\ Cl_2$	Int lab	MR	N – 3 mg/kg	Single oral dose – 35 μCi	Neathery et al., 1972 [141]
Corn forage	139	$^{65}Zn\ Cl_2$	Int lab	MR	N – 3 mg/kg	Single oral dose – 35 μCi	Neathery et al., 1972 [141]
Zinc carbonate	58, 58*	Zinc sulfate	Pla Zn	MR	N – 40 mg/kg	Single oral dose – 20 mg/kg BW	Kincaid, 1979 [142]
Zinc chloride	42, 42*	Zinc sulfate	Pla Zn	MR	N – 40 mg/kg	Single oral dose – 20 mg/kg BW	Kincaid, 1979 [142]
Zinc lysine	100*	Zinc sulfate RG	Liv Zn		N – 17 mg/kg	23 mg/kg	Engle et al., 1997 [143]
Zinc lysine + methionine	105	ZnO	Liv Zn			300	Kincaid, 1979 [142]
Zinc methionine	98*	Zinc sulfate RG	Liv Zn		N – 17 mg/kg	23	Engle et al., 1997 [143]
Zinc – methionine	103	Zinc oxide	Milk yield	MR	N – 50 mg/kg	400 mg/day	Kincaid et al., 1984 [144]
Zinc – methionine	133	Zinc oxide	Milk somatic cell counts	MR	N – 50 mg/kg	400 mg/day	Kincaid et al., 1984 [144]
Zinc – methionine (4 % Zn)	99	Zinc oxide	Growth	MR	N – 81 mg/kg	360 mg/day	Greene et al., 1988 [145]
Zinc – methionine	106	ZnO	Growth	MR	N	25	Spears, 1989 [146]

Zinc methionine	99*	Zinc sulfate RG	App abs		N	20	Spears, 1989 [146]
Zinc – methionine	105	Zinc oxide	Growth	MR	N	2500 mg/kg in mineral supplement	Spears and Kegley, 1991 [147]
Zinc methionine	105*	Zinc sulfate RG	App abs			30	Nockels et al., 1993 [9]
ZnO	100	$ZnSO_4.H_2O$	Wound healing	MR	N – 30 mg/kg	400	Miller et al., 1967 [148]
Zinc oxide	98, 101*	Zinc sulfate	Liv Zn	MR	N – 33 mg/kg	600	Miller et al., 1970 [149]
Zinc oxide	98	Zinc sulfate	App abs	MR	N – 33 mg/kg	600	Miller et al., 1970 [149]
Zinc oxide	98, 98*	Zinc sulfate	Pla Zn	MR	N – 40 mg/kg	Single oral dose – 20 mg/kg BW	Kincaid, 1979 [142]
Zinc oxide	98*	Zinc sulfate RG	App abs			20	Spears, 1989 [146]
Zinc – polysaccharide complex	144	ZnO	Rum fl microbial Zn	MR	N	172	Kennedy et al., 1988 [150]

Int = intrinsic; Lab = label; MR = mean ratio; N = natural; Pla = plasma; Liv = liver; abs = absorption; Rum = rumen; Fl = fluid; RG = reagent grade

Poultry

Table S13. Chickens

**Source**	**RV, %**	**Q_f**	**Standard**	**Response Criterion**	**Method Calculation**	**Type diet**	**Added level, mg/kg**	**Reference**
Beef	97		$ZnCO_3$	Growth	SR	P	24	Hempe, 1987 [151]
Beef	117		$ZnCO_3$	Pla Zn	SR	P	24	Hempe, 1987 [151]
Beef	102		$ZnCO_3$	Tibia Zn	SR	P	24	Hempe, 1987 [151]
Beef (SPC & Egg white diets)	100 (111 – 116)		$ZnCO_3$	Tibia Zn	SR	P	8 – 22	Hortin et al., 1991 [152]
Corn	63		$ZnCO_3$	Growth	MR	P	5 – 10	O'Dell et al., 1972 [153]
Corn, high lysine	65		$ZnCO_3$	Growth	MR	P	5 – 10	O'Dell et al., 1972 [153]

Corn germ	54		$ZnCO_3$	Growth	MR	P	5 – 10	O'Dell et al., 1972 [153]
Corn germ, high lysine	56		$ZnCO_3$	Growth	MR	P	5 – 10	O'Dell et al., 1972 [153]
Egg yolk	79		$ZnCO_3$	Growth	MR	P	5 – 10	O'Dell et al., 1972 [153]
Egg white	40		$ZnSO_4.7H_2O$	Growth	SR	P	7 - 10	Edwards and Baker, 2000 [154]
Fe-$ZnSO_4.H_2O$	107*		$ZnSO_4.H_2O$ FG	Per, Bone Zn			4.88 (5.02), 9.75 (10.04) (reference) 5.01 – 10.22	Edwards et al., 1998 [155]
Fish meal	75		$ZnCO_3$	Growth	MR	P	5 – 10	O'Dell et al., 1972 [153]
Franklinite (Fe, Mn, Zn, $FeO_2)_2$ (16.3 % Zn)	70, 70*		$ZnSO_4.7H_2O$ RG (22.7 % Zn)	Growth	MR	SP	10, 20	Edwards, 1959 [156]
Hemimorphite ($2ZnO.SiO_2.H_2O$)	98, 98*		$ZnSO_4.7H_2O$ RG	Growth	MR	SP	10, 20	Edwards, 1959 [156]
Milk, defatted	82		$ZnCO_3$	Growth	MR	P	5 – 10	O'Dell et al., 1972 [153]
Ore – franklinite + willemite + calcite (16.8 % Zn)	95		$ZnSO_4.7H_2O$ RG	Growth	MR	SP	10, 20	Edwards, 1959 [156]
Oysters	95		$ZnCO_3$	Growth	MR	P	5 – 10	O'Dell et al., 1972 [153]
Pork liver (Barrow, Gilt)	100 (118, 137)		$ZnSO_4.H_2O$	Bone	SR	P - 13	4	Aoyagi et al., 1995 [157]
Rice	62		$ZnCO_3$	Growth	MR	P	5 – 10	O'Dell et al., 1972 [153]
Sesame meal	59		$ZnCO_3$	Growth	MR	P	5 – 10	O'Dell et al., 1960 [158]
Smithsonite ($ZnCO_3$) (46.8 % Zn)	96		$ZnSO_4.7H_2O$ RG	Growth	MR	SP	10, 20	Edwards, 1959 [156]
Soybean meal	61, 67		$ZnCO_3$	Growth	MR	P	5 – 10	O'Dell et al., 1972 [153]
SBM, SPC, SPI	78, 65, 74		$ZnSO_4.7H_2O$	Growth	SR	P – 14	7 – 10	Edwards and Baker, 2000 [154]

SBM, SPC, SPI	41, 34, 39		$ZnSO_4.7H_2O$					Edwards and Baker, 1999 [159]
Sphalerite (ZnS) (64.2 % Zn)	60, 60*		$ZnSO_4.7H_2O$ RG	Growth	MR	SP	10, 20	Edwards, 1959 [156]
Wheat	59		$ZnCO_3$	Growth	MR	P	5 – 10	O'Dell et al., 1972 [153]
Willemite Zn_2SiO_4 (46.7 %)	103, 103*		$ZnSO_4.7H_2O$ RG	Growth	MR	SP	10, 20	Edwards, 1959 [156]
Zinc amino acid chelate (9.42 % Zn)	83, 76		$ZnSO_4.7H_2O$ RG	Bone Zn, Mucosa	SR	N – 59 mg/kg	200, 400	Cao et al., 2000 [160]
Zinc carbonate	100		Zinc chloride	Growth	MR	P – 6 mg/kg	20, 40	Pensack et al., 1958 [161]
Zinc carbonate	107, 109*		Zinc sulfate	Growth	TP	P – 10 mg/kg	10, 20	Roberson and Schaible, 1960a [162]
Zinc carbonate	123		Zinc sulfate	Decreased growth	TP	N	1000, 2000, 3000	Roberson and Schaible, 1960b [163]
Zinc chloride	99		Zinc sulfate	Growth	TP	P – 10 mg/kg	100	Roberson and Schaible, 1958 [164]
Zinc chloride basic	107*		$ZnSO_4.7H_2O$ RG	Bone Zn			(200), 400	Cao et al., 2000 [160]
Zinc chloride basic	110, 98-119		$ZnSO_4.7H_2O$ RG	Growth, Bone, Pla ALP, Cu/Zn SOD, Pan MT mRNA			20, 40, 80	Yu et al., 2021 [165]
Zinc – methionine	79		$ZnCl_2$	^{65}Zn abs by gut sacs	MR		10 μCi	Hill et al., 1987 [166]
Zinc – methionine FG	100		Zinc oxide	Bone Zn	TP	SP – 8 mg/kg	10, 20, 30, 40, 50	Pimentel et al., 1991 [167]
Zinc proteinate	105	1.98	$ZnSO_4.7H_2O$ RG	Bone Zn	SR	N -26.45 mg/kg	10, 20, 40, 80	Liu et al., 2013 [168]
Zinc proteinate	104	1.98	$ZnSO_4.7H_2O$ RG	Pan MT mRNA	SR	N -26.45 mg/kg	10, 20, 40, 80	Liu et al., 2013 [168]
Zinc sulphate basic	101*		$ZnSO_4.7H_2O$ RG	Bone Zn			(200), 400	Cao et al., 2000 [160]

Zinc sulfate tribasic ($Zn_4(OH)_6SO_4$), (54.8 % Cu) Day 6, 14	96, 100		$ZnSO_4.7H_2O$ RG	Bone Zn	SR	N – 27 mg/kg	30, 60, 90	Li et al., 2015 [169]
Zinc sulfate tribasic ($Zn_4(OH)_6SO_4$), (54.8 % Cu) Day 6, 14	84, 116		$ZnSO_4.7H_2O$ RG	Pan Zn	SR	N – 27 mg/kg	30, 60, 90	Li et al., 2015 [169]
Zinc sulfate tribasic ($Zn_4(OH)_6SO_4$), (54.8 % Cu) Day 6, 14	88, 124		$ZnSO_4.7H_2O$ RG	Pla Zn	SR	N – 27 mg/kg	30, 60, 90	Li et al., 2015 [169]
Zinc sulfate tribasic ($Zn_4(OH)_6SO_4$), (54.8 % Cu) Day 6, 14	76, 100		$ZnSO_4.7H_2O$ RG	Pan MT mRNA	SR	N – 27 mg/kg	30, 60, 90	Li et al., 2015 [169]
Zinc oxide	49*		$ZnSO_4.7H_2O$ RG	Bone Zn			(200), 400	Cao et al., 2000 [160]
Zinc oxide	100		Zinc chloride	Growth	MR	P – 6 mg/kg	20, 40	Pensack et al., 1958 [161]
Zinc oxide	108, 105*		Zinc sulfate	Growth	TP	P – 10 mg/kg	10, 20	Roberson and Schaible, 1960a [162]
Zinc oxide	61		Zinc sulfate	Decreased growth	MR	N	1000, 2000, 3000	Roberson and Schaible, 1960b [163]
Zinc oxide	67* 37,		$ZnSO_4.7H_2O$ RG	Per, Bone Zn	SR	P – 13.5 mg/kg	4.73-10.52	Edwards and Baker, 1999 [159]
Zinc oxide FG: (Assay 1) 1 (7.13 mg/kg), 2 (8.48 mg/kg) 3 (8.33 mg/kg)	94, 32, 47		$ZnSO_4.7H_2O$ RG	Growth	SR	P – 13.5 mg/kg	4.76, 9.90	Edwards and Baker, 1999 [159]
Zinc oxide FG: (Assay 1) 1 (7.13 mg/kg), 2 (8.48 mg/kg) 3 (8.33 mg/kg)	91, 22, 44		$ZnSO_4.7H_2O$ RG	Bone Zn	SR	P – 13.5 mg/kg	4.76, 9.90	Edwards and Baker, 1999 [159]
Zinc, elemental powder (100.0 % Zn)	102, 102*		$ZnSO_4.7H_2O$ RG	Growth	MR	SP	10, 20	Edwards, 1959 [156]
Zincite ZnO (65.3 % Zn)	97		$ZnSO_4.7H_2O$ RG	Growth	MR	SP	10, 20	Edwards, 1959 [156]
Zn amino acid complex (phytase	103		$ZnSO_4.7H_2O$	Growth	SR	SP - 44	10, 20, 40	Swiatkiewicz et al., 2001 [170]

supplemented, 750 FTU/kg)								
Zn amino acid complex (phytase supplemented, 750 FTU/kg)	104		$ZnSO_4.7H_2O$	Feed conversion	SR	SP - 44	10, 20, 40	Swiatkiewicz et al., 2001 [170]
Zn amino acid complex (phytase supplemented, 750 FTU/kg)	114, 114, 109		$ZnSO_4.7H_2O$	Bone Zn (dried tibia, tibia ash, total)	SR	SP - 44	10, 20, 40	Swiatkiewicz et al., 2001 [170]
Zn amino acid complex (no phytase supplement)	121		$ZnSO_4.7H_2O$	Growth	SR	SP - 44	10, 20, 40	Swiatkiewicz et al., 2001 [170]
Zn amino acid complex (no phytase supplement)	116		$ZnSO_4.7H_2O$	Feed conversion	SR	SP - 44	10, 20, 40	Swiatkiewicz et al., 2001 [170]
Zn amino acid complex (no phytase supplement)	139, 142, 117		$ZnSO_4.7H_2O$	Bone Zn (dried tibia, tibia ash, total)	SR	SP - 44	10, 20, 40	Swiatkiewicz et al., 2001 [170]
Zn amino acid complex	164		$ZnSO_4.7H_2O$ RG	Bone Zn	SR	N - 34	5, 10, 15	Star et al., 2012 [171]
Zn amino acid complex C (11.93 % Zn)	100, 105, 101, 103	6.5	$ZnSO_4$ RG	Pan MT mRNA, Bone, Pan, Pan MT	SR	N – 28 mg/kg	30, 60, 90	Huang et al., 2009 [172]
Zn amino acid L (119 g Zn/kg))	101	6.6	$ZnSO_4.7H_2O$	Pan MT mRNA	SR	N – 28 mg/kg, low phytate (4.6 g)	30, 60	Huang et al., 2013 [173]
Zn amino acid L (119 g Zn/kg))	105	6.6	$ZnSO_4.7H_2O$	Pan MT mRNA	SR	N – 28 mg/kg, high phytate (14.2 g)	30, 60	Huang et al., 2013 [173]
$ZnCO_3$ RG (52.1 % Zn)	100, 98*		$ZnSO_4.7H_2O$ RG	Growth	MR	SP	10, 20	Edwards, 1959 [156]
$ZnCO_3$ RG	78*		$ZnSO_4.7H_2O$ RG	Bone Zn	SR	N – 63 mg/kg	400, 800, 1200	Sandoval et al., 1997 [174]

$Zn_5Cl_2(OH)_8$, Tetrabasic zinc chloride (TBZC) (60.2 % Zn)	102, 111		$ZnSO_4.7H_2O$	Growth	SR	P	5.81, 10.81, 15.10, 20.25	Batal et al., 2001 [175]
Zn-$FeSO_4.H_2O$	99*		$ZnSO_4.H_2O$ FG	Per, Bone Zn		P – 13.5 mg/kg	4.88 (5.02), 9.75 (10.04) (reference) 5.01 – 10.22	Edwards et al., 1998 [155]
Zn – lysine (7.21 % Zn)	106, 111		$ZnSO_4.H_2O$ FG	Bone Zn	SR	N – 13	2.9, 5.8	Aoyagi and Baker, 1993b [23]
Zn metal RG	46*		$ZnSO_4.7H_2O$ RG	Bone Zn	SR	N – 63 mg/kg	400, 800, 1200	Sandoval et al., 1997 [174]
Zn metal dust (100 % Zn) (7.88 mg/kg) (Assay 2)	67*		$ZnSO_4.7H_2O$ RG	Growth	SR	P – 13.5 mg/kg	5.06, 10.12	Edwards and Baker, 1999 [159]
Zn metal fume (91.5% Zn), (10.52 mg/kg) (Assay 3)	36*		$ZnSO_4.7H_2O$ RG	Growth	SR	P – 13.5 mg/kg	4.73, 9.13	Edwards and Baker, 1999 [159]
Zn methionine	121*		$ZnSO_4.H_2O$	Bone Zn, Per			7.5, 15	Wedekind and Baker, 1990 [176]
Zn – methionine (+ 0.6 or 0.74% Ca)	166, 292		$ZnSO_4.H_2O$ RG	Bone Zn	SR	P – amino acid	5, 10	Wedekind 1994 [177]
Zn methionine (Day 3, 6, 9)	91, 94, 77		Zn acetate RG	Bone Zn	SR	N – 24 mg/kg	30, 60	Cao et al., 2002 [178]
Zn methionine (Day 3, 6, 9)	88, 91, 78		Zn acetate RG	Mucosal MT	SR	N – 24 mg/kg	30, 60	Cao et al., 2002 [178]
Zn methionine chelate (17.6 % Zn) (Day 7)	99.4, 103, 120		$ZnSO_4.7H_2O$	Bone, Pancreas, Pan MT mRNA	SR	N – 29 mg/kg	30, 60, 90	Suo et al., 2015 [179]
Zn methionine chelate (17.6 % Zn) (Day 21)	107, 115, 106		$ZnSO_4.7H_2O$	Bone, Pancreas, Pan MT mRNA	SR	N – 29 mg/kg	30, 60, 90	Suo et al., 2015 [179]
Zn – methionine FG	124, 136*		$ZnSO_4.H_2O$ RG	Growth	SR	P	7.5, 15	Wedekind et al., 1992 [180]
Zn – methionine FG	176[1]		$ZnSO_4.H_2O$ RG	Bone Zn	SR	SP – soy isolate	7.5, 15	Wedekind et al., 1992 [180]

Zn – methionine FG	206[1,2]		$ZnSO_4.H_2O$ RG	Bone Zn	SR	C – SBM	7.5, 15	Wedekind et al., 1992 [180]
Zn – methionine FG	117[1]		$ZnSO_4.H_2O$ RG	Bone Zn	SR	P – crystalline amino acid	7.5, 15	Wedekind et al., 1992 [180]
Zn methionine hydroxy analog chelate (Zn bis(-2-hydroxy-4-(methylthio)butanoic acid) ZnHMTBa	161, 165, 248		$ZnSO_4.H_2O$ RG	Bone (total), Bone conc. MT mRNA	SR	C-SBM – 21 mg/kg	5, 10, 15, 20, 30	Richards et al., 2015 [181]
Zn methionine hydroxy analog chelate (Zn bis(-2-hydroxy-4-(methylthio)butanoic acid) ZnHMTBa	441, 307, 426		$ZnSO_4.H_2O$ RG	Bone (total), Bone conc. MT mRNA	SR	C-SBM – 27 mg/kg + Ca (1.2 %) + P (1.3 %)	7.5, 15, 30	Richards et al., 2015 [181]
ZnO RG	97, 99*		$ZnSO_4.7H_2O$ RG	Growth	MR	SP	10, 20	Edwards, 1959 [156]
ZnO (79.6 % Zn)	104		$ZnSO_4.7H_2O$ RG	Growth	MR	SP	10, 20	Edwards, 1959 [156]
ZnO FG (72 % Zn)	61		$ZnSO_4.H_2O$ RG	Growth	SR	P – 13 mg/kg	7.5, 15	Wedekind and Baker, 1990 [176]
ZnO FG (72 % Zn)	44, 44*		$ZnSO_4.H_2O$ RG	Bone Zn	SR	P – 13 mg/kg	7.5, 15	Wedekind and Baker, 1990 [176]
ZnO	53		$ZnSO_4.H_2O$ RG	Growth, Bone Zn	SR	P	7.5, 15	Wedekind et al., 1992 [180]
ZnO	36*		$ZnSO_4.H_2O$ RG	Bone Zn, Per			7.5, 15	Wedekind and Baker, 1990 [176]
ZnO RG	72*, 77		$ZnSO_4.7H_2O$ RG	Bone Zn	SR	N – 63 mg/kg	400, 800, 1200	Sandoval et al., 1997 [174]
ZnO FG – A	78		$ZnSO_4.7H_2O$ RG	Bone Zn	SR	N – 75 mg/kg	300, 600, 900	Sandoval et al., 1997 [174]
ZnO FG – B	54		$ZnSO_4.7H_2O$ RG	Bone Zn	SR	N – 75 mg/kg	300, 600, 900	Sandoval et al., 1997 [174]
ZnO FG	74		$ZnSO_4.7H_2O$ RG	Bone Zn	SR	N – 35 mg/kg	40, 80, 120	Sandoval et al., 1997 [174]

ZnO AG (7.46 mg/kg), FG-1 (7.75 mg/kg), FG-2 (8.23 mg.kg) (Assay 2)	89, 97, 41		$ZnSO_4.7H_2O$ RG	Growth	SR	P – 13.5 mg/kg	5.06, 10.12	Edwards and Baker, 1999 [159]
ZnO FG 1 (6.71 mg/kg), FG-2 (8.98 mg/kg), FG-4 (6.69 mg.kg) (Assay 3)	93, 39, 84		$ZnSO_4.7H_2O$ RG	Growth	SR	P – 13.5 mg/kg	4.73, 9.13	Edwards and Baker, 1999 [159]
Zn oxide FG 1 (72 % Zn)	59, 99, 45		$ZnSO_4.H_2O$	Growth	SR	N – 26 mg/kg	100, 150, 200	Sahraei et al., 2013 [182]
Zn oxide FG 2 (75 % Zn)	64, 78, 31		$ZnSO_4.H_2O$	Growth	SR	N – 26 mg/kg	100, 150, 200	Sahraei et al., 2013 [182]
Zn polysaccharide complex (19.02 % Zn)	94	3.8	$ZnSO_4.7H_2O$ RG	Bone Zn	SR	N – 100 mg/kg	200	Cao et al., 2000 [160]
Zn propionate	119		Zinc sulfate FG	Growth	SR	N – 21 mg/kg	6, 12	Brooks et al., 2013 [183]
Zn propionate	116, 116		Zinc sulfate FG	Bone Zn, total tibia Zn	SR	N – 21 mg/kg	6, 12	Brooks et al., 2013 [183]
Zn proteinate A (13.63 % Zn)	139, 133	13	$ZnSO_4.7H_2O$ RG	Bone Zn, Mucosa	SR	N – 59 mg/kg	200, 400	Cao et al., 2000 [160]
Zn proteinate B (13.65 % Zn)	99	91	$ZnSO_4.7H_2O$ RG	Bone Zn	SR	N – 100 mg/kg	200	Cao et al., 2000 [160]
Zn proteinate C (13.01 % Zn)	108	120	$ZnSO_4.7H_2O$ RG	Bone Zn	SR	N – 100 mg/kg	200	Cao et al., 2000 [160]
Zn proteinate (Day 3, 6, 9)	110, 124, 116		Zn acetate RG	Bone Zn	SR	N – 24 mg/kg	30, 60	Cao et al., 2002 [178]
Zn proteinate (Day 3, 6, 9)	128, 99, 130		Zn acetate RG	Mucosal MT	SR	N – 24 mg/kg	30, 60	Cao et al., 2002 [178]
Zn proteinate	183		$ZnSO_4.7H_2O$ RG	Growth	SR	N – 23 mg/kg	5, 10, 20, 40	Ao et al., 2006 [184]
Zn proteinate	157		$ZnSO_4.7H_2O$ RG	Bone Zn	SR	N – 23 mg/kg	5, 10, 20, 40	Ao et al., 2006 [184]
Zinc proteinate	100		Zinc chloride	Growth	MR	P – 6 mg/kg	20, 40	Pensack et al., 1958 [161]
Zn proteinate A (18.61 % Zn)	72, 97, 101,	944	$ZnSO_4$ RG	Pan MT mRNA, Bone,	SR	N – 28 mg/kg	30, 60, 90	Huang et al., 2009 [172]

	92			Pan,<br>Pan MT				
Zn proteinate B (13.27 % Zn)	121,<br>106,<br>104,<br>111	30.7	$ZnSO_4$ RG	Pan MT mRNA,<br>Bone,<br>Pan,<br>Pan MT	SR	N – 28 mg/kg	30, 60, 90	Huang et al., 2009 [172]
Zn proteinate M (133 g Zn/kg)	128	30.7		Pan MT mRNA	SR	N – 28 mg/kg, low phytate (4.6 g)	30, 60	Huang et al., 2013 [173]
Zn proteinate H (186 g Zn/kg)	70	944		Pan MT mRNA	SR	N – 28 mg/kg, low phytate (4.6 g)	30, 60	Huang et al., 2013 [173]
Zn proteinate M (133 g Zn/kg)	139	30.7		Pan MT mRNA	SR	N – 28 mg/kg, high phytate (14.2 g)	30, 60	Huang et al., 2013 [173]
Zn proteinate H (186 g Zn/kg)	92	944		Pan MT mRNA	SR	N – 28 mg/kg, high phytate (14.2 g)	30, 60	Huang et al., 2013 [173]
Zn proteinate (15 % Zn)	151,<br>200,<br>147		$ZnSO_4.H_2O$	Growth	SR	N – 26 mg/kg	100, 150, 200	Sahraei et al., 2013 [182]
$ZnSO_4.H_2O$ FG (7.29 mg/kg)<br>FG – 1 (7.25 mg/kg)<br>FG -2 (7.04 mg/kg) (Assay 2)	87*,<br>89,<br>86,<br>87		$ZnSO_4.7H_2O$ RG	Growth		P – 13.5 mg/kg	5.06, 10.12	Edwards and Baker, 1999 [159]
$ZnSO_4$ FG	89*		$ZnSO_4.7H_2O$ RG	Bone Zn	SR	N – 63 mg/kg	300, 600, 900	Sandoval et al., 1997 [174]
$ZnSO_4$ FG – A (granular)	99		$ZnSO_4.7H_2O$ RG	Bone Zn	SR	N – 75 mg/kg	300, 600, 900	Sandoval et al., 1997 [174]
$ZnSO_4$ FG – B (spray dried)	81		$ZnSO_4.7H_2O$ RG	Bone Zn	SR	N – 75 mg/kg	300, 600, 900	Sandoval et al., 1997 [174]

$ZnSO_4$ FG	94		$ZnSO_4.7H_2O$ RG	Bone Zn	SR	N – 35 mg/kg	40, 80, 120	Sandoval et al., 1997 [174]

MR = mean ratio; SR = slope ratio; TP = three-point; P = purified; SP = semi-purified; N = natural; C-SBM = corn-soybean meal; Pla = plasma; RG = reagent grade; FG = feed grade; Per = performance; SBM = soybean meal; SPC = soy protein concentrate; SPI = soy protein isolate; ALP = alkaline phosphatase; Cu/Zn SOD = Cu, Zn – superoxide dismutase; Pan = pancreatic; MT = metallothionein; mRNA = messenger ribonucleic acid; abs = absorption;

[1] An important consideration concerning the mean RBV of 131 % ± 10.9 for Zn methionine is that among other factors, the type of diet has a considerable effect on the relative bioavailability estimate (Wedekind et al., 1992). Relative to $ZnSO_4.H_2O$ FG, the RBV for Zn methionine assayed on an amino acid diet, a soy-isolate diet and a practical corn-soybean diet was found to be 117 % over 177 % to 206 % respectively. It is assumed that this is due to the amount of phytate and soluble fibre, which forms complexes with the Zn of inorganic origin.

[2] Later studies (94) by same author in swine did not show similar results for Zn met and in fact RBV was below that of the sulfate.

Table S14. Turkeys

Source	RV, %	Standard	Response Criterion	Method Calculation	Type diet	Added level, mg/kg	Reference
1,2 – Diaminocyclohexane-tetraacetic acid – Zn ($ZnC_{14}H_{20}O_8N_2$)	108	ZnO	Growth	TP	P – 17 mg/kg	15	Vohra and Kratzer, 1966 [185]
Diethylenetriamine-pentaacetic acid – Zn ($ZnC_{14}O_{10}H_{23}N_3$)	118	ZnO	Growth	TP	P – 17 mg/kg	15	Vohra and Kratzer, 1966 [185]
Zinc carbonate (54.0 % Zn)	100	$ZnSO_4.7H_2O$ RG	Growth	TP	P – 14 mg/kg	20, 30, 40	Sullivan, 1961 [186]
Zinc citrate [$Zn_3(C_6H_5O_7)_2.2H_2O$	128	ZnO	Growth	TP	P – 17 mg/kg	15	Vohra and Kratzer, 1966 [185]
Zinc – EDTA ($ZnC_{10}H_{14}O_8N_2$) (19.1 % Zn)	118	ZnO	Growth	TP	P – 17 mg/kg	15	Vohra and Kratzer, 1966 [185]
Zinc – EDTA	110	Zn	Growth	TP	P – 25 mg/kg	30	Kratzer et al., 1959 [187]
ZnCl2 RG (45.6 % Zn)	88	$ZnSO_4.7H_2O$ RG	Growth	TP	P – 14 mg/kg	20, 30, 40	Sullivan, 1961 [186]
$Zn_2HP_3O_{10}$ (29.7 % Zn)	69	ZnO	Growth	TP	P – 17 mg/kg	15	Vohra and Kratzer, 1966 [185]
ZnO (71.6 % Zn)	78	$ZnSO_4.7H_2O$ RG	Growth	TP	P – 14 mg/kg	20, 30, 40	Sullivan, 1961 [186]

$Zn_3(PO_4)_2.4H_2O$ (43.4 % Zn)	48	ZnO	Growth	TP	P – 17 mg/kg	15	Vohra and Kratzer, 1966 [185]
$Zn_2P_2O_{27}.4H_2O$ (34.9 % Zn)	61	ZnO	Growth	TP	P – 17 mg/kg	15	Vohra and Kratzer, 1966 [185]
$Zn_3P_6O_{18}$ (29.8 % Zn)	70	ZnO	Growth	TP	P – 17 mg/kg	15	Vohra and Kratzer, 1966 [185]
Zn – phytate [$Zn_6C_6H_6P_6O_{24}.3H_2O$] (19.1 % Zn)	76	ZnO	Growth	TP	P – 17 mg/kg	15	Vohra and Kratzer, 1966 [185]
$ZnSO_4.H_2O$ (36.4 % Zn)	75	$ZnSO_4.7H_2O$ RG	Growth	TP	P – 14 mg/kg	20, 30, 40	Sullivan, 1961 [186]

TP = three-point; P = purified; RG = reagent grade; EDTA = ethylenediaminetetraacetate

Table S15. Japanese Quail

**Source**	**RV, %**	**Standard**	**Response Criterion**	**Method Calculation**	**Type diet**	**Added level, mg/kg**	**Reference**
Casein (42 mg/kg Zn)	47[1]	-	^{65}Zn ext lab		P – 60 mg/kg	Single oral dose	Jones et al., 1985 [188]
Egg white (0.8 mg/kg Zn)	38[1]	-	^{65}Zn ext lab		P – 60 mg/kg	Single oral dose	Jones et al., 1985 [188]
Soy concentrate (37 mg/kg Zn)	25[1]	-	^{65}Zn ext lab		P – 60 mg/kg	Single oral dose	Jones et al., 1985 [188]
Soy flour (48 mg/kg Zn)	24[1]	-	^{65}Zn ext lab		P – 60 mg/kg	Single oral dose	Jones et al., 1985 [188]

Ext = extrinsic; Lab = label; P = purified

[1] Percentage absorption, not relative bioavailability value

Table S16. Sheep

**Source**	**RV, %**	**Q_f**	**Standard**	**Response Criterion**	**Method Calculation**	**Type diet**	**Added level, mg/kg**	**Reference**
Zinc amino acid complex	102*		Zinc sulfate RG	Liv Zn			90	Hatfield et al., 2001 [45]
Zinc amino acid chelate (9.42 % Zn)	110	180	$ZnSO_4.7H_2O$ RG	Liv, Kid, Pan, Liv MT	SR	N – 58 mg/kg	1400	Cao et al., 2000 [160]
Zinc carbonate RG	105*, 106		Zinc sulfate RG	Liv, Kid Zn	SR	N – 44 mg/kg	1400	Sandoval et al., 1997 [189]

Zinc, chelated	91, 96*		$ZnSO_4.H_2O$ RG	Pla Zn	MR	P	25	Ho and Hidiroglou, 1977 [190]
Zinc, chelated	125		$ZnSO_4.H_2O$ RG	Growth	MR	P	25	Ho and Hidiroglou, 1977 [190]
Zinc chelate	98*		Zinc sulfate RG	Liv, Kid Zn	SR		1400	Cao et al., 2000 [160]
Zinc EDTA	17		Zinc sulfate	Liv Zn	TP	N	240 mg/kg BW	Smith and Embling, 1984 [191]
Zinc glycine	335, 82, 86, 179		$ZnSO_4.H_2O$ RG	Pla, Liv, Kid, Liv Cu/Zn SOD	SR	N – 30 mg/kg	50	Gresakova et al., 2021 [192]
Zinc lysine	114*		Zinc sulfate RG	Liv, Kid Zn			360 mg/kg	Rojas et al., 1995 [193]
Zinc metal RG	95*, 76, 68		Zinc sulfate RG	Liv, Kid Zn	SR	N – 44 mg/kg	1400	Sandoval et al., 1997 [189]
Zinc methionine	100*		Zinc sulfate RG	Liv, Kid Zn		N – 20 mg/kg	360 mg/kg	Rojas et al., 1995 [193]
Zinc – methionine	95		ZnO	Pla Zn	MR	SP – 3 mg/kg	5	Spears, 1989 [146]
Zinc – methionine	103		ZnO	App abs	MR	SP – 3 mg/kg	5	Spears, 1989 [146]
Zinc – methionine	99*		Zinc sulfate RG	App abs			20	Spears, 1989 [146]
Zinc methionine A	98*	1.9	Zinc sulfate RG	Liv, Kid Zn	SR		1400	Cao et al., 2000 [160]
Zinc methionine B (9.29 %)	113	1.5	$ZnSO_4.7H_2O$ RG	Liv, Kid, Pan, Liv MT	SR		1400	Cao et al., 2000 [160]
Zinc methionine FG	133		Zn sulfate FG	Liv Cu	SR	N – 76 mg/kg	94	Pal et al., 2010 [49]
Zinc methionine FG	132		Zn sulfate FG	Pla Cu	SR	N – 76 mg/kg	94	Pal et al., 2010 [49]
Zinc methionine FG	134		Zn sulfate FG	Gut abs	SR	N – 76 mg/kg	94	Pal et al., 2010 [49]
Zinc oxide	93*		Zinc sulfate RG	Liv, Kid Zn		N – 20 mg/kg	360 mg/kg	Rojas et al., 1995 [193]
Zinc oxide	100*, 106		Zinc sulfate RG	Liv, Kid Zn	SR	N – 44 mg/kg	1400	Sandoval et al., 1997 [189]

Zinc oxide A, B	87, 79, 80, 74		Zinc sulfate RG	Liv, Kid Zn	SR	N – 64 mg/kg	1400	Sandoval et al., 1997 [189]
Zinc proteinate	102*	13	Zinc sulfate RG	Liv, Kid Zn	SR		1400	Cao et al., 2000 [160]
Zn proteinate A (13.63 % Zn)	130	13	$ZnSO_4.7H_2O$ RG	Liv, Kid, Pan, Liv MT	SR		1400	Cao et al., 2000 [160]
Zn proteinate	254, 56, 126, 58		$ZnSO_4.H_2O$ RG	Plas, Liv, Kid, Liv Cu/Zn SOD	SR	N – 30 mg/kg	80	Gresakova et al., 2021 [192]
Zinc sulfate FG – B	99*, 86, 83		Zinc sulfate RG	Liv, Kid Zn	SR	N – 64 mg/kg	1400	Sandoval et al., 1997 [189]
Zinc, sequestered	108, 97*		$ZnSO_4.H_2O$ RG	Plas Zn	MR	P	25	Ho and Hidiroglou, 1977 [190]
Zinc, sequestered	103		$ZnSO_4.H_2O$ RG	Growth	MR	P	25	Ho and Hidiroglou, 1977 [190]

RG = reagent grade; Liv = liver; Kid = kidney; Pan = pancreatic; Pla = plasma; MT = metallothionein; SR = slope ratio, MR = mean ratio; TP = three-point; N = natural; P = purified; SP = semi-purified; Cu/Zn SOD = Cu, Zn-superoxide dismutase; App = apparent; Abs = absorption

Table S17. Swine

**Source**	**RV, %**	**Standard**	**Response Criterion**	**Method Calculation**	**Type diet**	**Added level, mg/kg**	**Reference**
Zinc amino acid chelate	102*	$ZnSO_4.xH_2O$	Pan Zn			45	Swinkels et al., 1996 [194]
Zinc carbonate	98*	$ZnSO_4.7H_2O$	Abs			127	Hap and Zeman, 1994 [195]
Zinc chloride, tetrabasic (TBZC) ($Zn_5(OH)_8Cl_2.H_2O$) (58 % Zn) – week 2	159	ZnO	Pla Zn	SR	N - 208	1500, 2250, 3000	Zhang and Guo, 2007 [196]
Zinc chloride, tetrabasic (TBZC) – week 4	125, 128, 123, 122	ZnO	Pla, Liv, Kid, Bone	SR	N - 208	1500, 2250, 3000	Zhang and Guo, 2007 [196]

Zinc – lysine	100, 110	$ZnSO_4$	Pla Zn	SR	N	3000, 5000	Hahn and Baker, 1993 [197]
Zinc lysine	98*	$ZnSO_4.H_2O$	Abs, Ser, Bone			100	Cheng et al., 1998 [198]
Zinc lysine	92*	$ZnSO_4.xH_2O$	Ser, Bone			1000, 2000, 3000	Schell and Kornegay, 1996 [199]
Zinc lysine	64*	$ZnSO_4.H_2O$	Per, Bone, Pla	SR	N – 32p pm	5, 10, 20, 40, 80	Wedekind et al., 1994 [200]
Zinc lysine	38	$ZnSO_4.H_2O$	Metacarpal	SR	N – 32p pm	5, 10, 20, 40, 80	Wedekind et al., 1994 [200]
Zin lysine	24	$ZnSO_4.H_2O$	Coccygeal vertebrae	SR	N – 32p pm	5, 10, 20, 40, 80	Wedekind et al., 1994 [200]
Zinc lysine	79	$ZnSO_4.H_2O$	Pla Zn	SR	N – 32p pm	5, 10, 20, 40, 80	Wedekind et al., 1994 [200]
Zinc metal dust (99.3 % Zn)	133, 105*	Zinc oxide	Ser Zn	SR	N – 20 mg/kg	25, 50	Miller et al., 1981 [201]
Zinc methionine	101*	$ZnSO_4.H_2O$	Per, Bone, Ser			9, 12, 15	Hill et al., 1986 [202]
Zinc – methionine	100	$ZnCl_2$	^{65}Zn abs by gut sacs	MR	-	10 μCi	Hill et al., 1987 [166]
Zinc – methionine	>100, 116	$ZnSO_4$	Pla Zn	SR	N	3000, 5000	Hahn and Baker, 1993 [197]
Zinc methionine	95*	$ZnSO_4.xH_2O$	Ser, Bone			1000, 2000, 3000	Schell and Kornegay, 1996 [199]
Zinc methionine	86*	$ZnSO_4.H_2O$	Per, Bone, Pla	SR	N – 32mg/kg	5, 10, 20, 40, 80	Wedekind et al., 1994 [200]
Zinc methionine	60	$ZnSO_4.H_2O$	Metacarpal	SR	N – 32mg/kg	5, 10, 20, 40, 80	Wedekind et al., 1994 [200]
Zinc methionine	84	$ZnSO_4.H_2O$	Coccygeal vertebrae	SR	N – 32mg/kg	5, 10, 20, 40, 80	Wedekind et al., 1994 [200]
Zinc methionine	95	$ZnSO_4.H_2O$	Pla Zn	SR	N – 32mg/kg	5, 10, 20, 40, 80	Wedekind et al., 1994 [200]
Zinc methionine complex (Zn Org)	100	$ZnSO_4.7H_2O$	Growth	SR	N - 28	10, 20, 30	Dourmad et al., 2002 [203]
Zinc oxide	50, 56	$ZnSO_4$	Pla Zn	SR	N	3000, 5000	Hahn and Baker, 1993 [197]

Zinc oxide	110*	$ZnSO_4.7H_2O$	Abs			127	Hap and Zeman, 1994 [195]
Zinc oxide	87*	$ZnSO_4.xH_2O$	Ser, Bone			1000, 2000, 3000	Schell and Kornegay, 1996 [199]
Zinc oxide	82*	$ZnSO_4.H_2O$	Per, Bone, Pla	SR	N – 32mg/kg	5, 10, 20, 40, 80	Wedekind et al., 1994 [200]
Zinc oxide	67	$ZnSO_4.H_2O$	Metacarpal	SR	N – 32mg/kg	5, 10, 20, 40, 80	Wedekind et al., 1994 [200]
Zinc oxide	70	$ZnSO_4.H_2O$	Coccygeal vertebrae	SR	N – 32mg/kg	5, 10, 20, 40, 80	Wedekind et al., 1994 [200]
Zinc oxide	87	$ZnSO_4.H_2O$	Pla Zn	SR	N – 32mg/kg	5, 10, 20, 40, 80	Wedekind et al., 1994 [200]

Pan = pancreatic; Abs = absorption; Pla = plasma; Liv = liver; Kid = kidney; SR = slope ratio, MR = mean ratio; N = natural; Ser = serum; Per = performance;

References:

1. Ammerman, C.B.; Baker, D.H.; Lewis, A.J. Preface. In *Bioavailability of Nutrients for Animals*, Ammerman, C.B., Baker, D.H., Lewis, A.J., Eds. Academic Press: San Diego, 1995; https://doi.org/10.1016/B978-012056250-3/50026-3pp. xiii-xiv.
2. De Groote, G., Lippens, M., Jongbloed, A.W. and Meschy, F. *Study on the bioavailability of major and trace minerals*; International Association of the European (EU) Manufacturers of Major, Trace and Specific Feed mineral Materials (Emfema): Brussels, 2002.
3. Ward, J.D.; Spears, J.W.; Kegley, E.B. Bioavailability of copper proteinate and copper carbonate relative to copper sulfate in cattle. *J. Dairy Sci.* **1996**, *79*, 127-132, doi:10.3168/jds.S0022-0302(96)76343-9.
4. Engle, T.E.; Spears, J.W. Effects of dietary copper concentration and source on performance and copper status of growing and finishing steers. *J. Anim. Sci.* **2000**, *78*, 2446-2451, doi:10.2527/2000.7892446x.
5. VanValin, K.R.; Genther-Schroeder, O.N.; Laudert, S.B.; Hansen, S.L. Relative bioavailability of organic and hydroxy copper sources in growing steers fed a high antagonist diet. *J. Anim. Sci.* **2019**, *97*, 1375-1383, doi:10.1093/jas/sky487.
6. Hansen, S.L.; Schlegel, P.; Legleiter, L.R.; Lloyd, K.E.; Spears, J.W. Bioavailability of copper from copper glycinate in steers fed high dietary sulfur and molybdenum. *J. Anim. Sci.* **2008**, *86*, 173-179, doi:10.2527/jas.2006-814.
7. Kegley, E.B.; Spears, J.W. Bioavailability of feed-grade copper sources (oxide, sulfate, or lysine) in growing cattle. *J. Anim. Sci.* **1994**, *72*, 2728-2734, doi:10.2527/1994.72102728x.
8. Chase, C.R.; Beede, D.K.; Van Horn, H.H.; Shearer, J.K.; Wilcox, C.J.; Donovan, G.A. Responses of lactating dairy cows to copper source, supplementation rate, and dietary antagonist (Iron). *J. Dairy Sci.* **2000**, *83*, 1845-1852, doi:https://doi.org/10.3168/jds.S0022-0302(00)75055-7.
9. Nockels, C.F.; DeBonis, J.; Torrent, J. Stress induction affects copper and zinc balance in calves fed organic and inorganic copper and zinc sources. *J. Anim. Sci.* **1993**, *71*, 2539-2545, doi:10.2527/1993.7192539x.
10. Ward, J.D.; Spears, J.W.; Kegley, E.B. Effect of copper level and source (copper lysine vs copper sulfate) on copper status, performance, and immune response in growing steers fed diets with or without supplemental molybdenum and sulfur. *J. Anim. Sci.* **1993**, *71*, 2748-2755, doi:10.2527/1993.71102748x.

11. Kincaid, R.L.; Blauwiekel, R.M.; Cronrath, J.D. Supplementation of Copper as Copper Sulfate or Copper Proteinate for Growing Calves Fed Forages Containing Molybdenum. *J. Dairy Sci.* **1986**, *69*, 160-163, doi:10.3168/jds.S0022-0302(86)80381-2.
12. Wittenberg, K.M.; Boila, R.J.; Shariff, M.A. Comparison of copper sulfate and copper proteinate as copper sources for copper-depleted steers fed high molybdenum diets. *Can. J. Anim. Sci.* **1990**, *70*, 895-904, doi:10.4141/cjas90-108.
13. Du, Z.; Hemken, R.W.; Harmon, R.J. Copper Metabolism of Holstein and Jersey Cows and Heifers Fed Diets High in Cupric Sulfate or Copper Proteinate. *J. Dairy Sci.* **1996**, *79*, 1873-1880, doi:https://doi.org/10.3168/jds.S0022-0302(96)76555-4.
14. Ivan, M.; Proulx, J.G.; Morales, R.; Codagnone, H.C.V.; Dayrell, M.d.S. Copper accumulation in the liver of sheep and cattle fed diets supplemented with copper sulfate or copper chloride. *Can. J. Anim. Sci.* **1990**, *70*, 727-730, doi:10.4141/cjas90-089.
15. Xin, Z.; Waterman, D.F.; Hemken, R.W.; Harmon, R.J.; Jackson, J.A. Effects of Copper Sources and Dietary Cation-Anion Balance on Copper Availability and Acid-Base Status in Dairy Calves. *J. Dairy Sci.* **1991**, *74*, 3167-3173, doi:https://doi.org/10.3168/jds.S0022-0302(91)78502-0.
16. Miltimore, J.E.; Kalnin, C.M.; Clapp, J.B. Copper storage in the livers of cattle supplemented with injected copper and with copper sulfate and chelated copper. *Can. J. Anim. Sci.* **1978**, *58*, 525-529, doi:10.4141/cjas78-068.
17. Spears, J.W., Kegley, E.B., Mullis, L.A. and Wise, T.A. Bioavailability of copper from tri-basic copper chloride in cattle. *J. Anim. Sci.* **1997**, *75*, 265.
18. Spears, J.; Kegley, E.; Mullis, L. Bioavailability of copper from tribasic copper chloride and copper sulfate in growing cattle. *Anim. Feed Sci. Technol.* **2004**, *116*, 1-13, doi:10.1016/j.anifeedsci.2004.06.002.
19. Kim, J.W.; Kil, D.Y. Determination of relative bioavailability of copper in tribasic copper chloride to copper in copper sulfate for broiler chickens based on liver and feather copper concentrations. *Anim. Feed Sci. Technol.* **2015**, *210*, 138-143, doi:https://doi.org/10.1016/j.anifeedsci.2015.09.022.
20. Kim, J.W.; Kim, J.H.; Shin, J.E.; Kil, D.Y. Relative bioavailability of copper in tribasic copper chloride to copper in copper sulfate for laying hens based on egg yolk and feather copper concentrations. *Poult. Sci.* **2016**, *95*, 1591-1597, doi:https://doi.org/10.3382/ps/pew049.
21. Pott, E.B.; Henry, P.R.; Ammerman, C.B.; Merritt, A.M.; Madison, J.B.; Miles, R.D. Relative bioavailability of copper in a copperlysine complex for chicks and lambs. *Anim. Feed Sci. Technol.* **1994**, *45*, 193-203, doi:https://doi.org/10.1016/0377-8401(94)90026-4.
22. Baker, D.H.; Odle, J.; Funk, M.A.; Wieland, T.M. Research note: bioavailability of copper in cupric oxide, cuprous oxide, and in a copper-lysine complex. *Poult. Sci.* **1991**, *70*, 177-179, doi:10.3382/ps.0700177.
23. Aoyagi, S.; Baker, D.H. Nutritional evaluation of copper-lysine and zinc-lysine complexes for chicks. *Poult. Sci.* **1993b**, *72*, 165-171, doi:10.3382/ps.0720165.
24. Aoyagi, S.; Baker, D.H. Nutritional evaluation of a copper-methionine complex for chicks. *Poult. Sci.* **1993d**, *72*, 2309-2315, doi:https://doi.org/10.3382/ps.0722309.
25. Aoyagi, S.; Baker, D.H.; Wedekind, K.J. Estimates of copper bioavailability from liver of different animal species and from feed ingredients derived from plants and animals. *Poult. Sci.* **1993**, *72*, 1746-1755, doi:https://doi.org/10.3382/ps.0721746.
26. Guo, R.; Henry, P.R.; Holwerda, R.; Cao, J.; Littell, R.C.; Miles, R.D.; Ammerman, C.B. Chemical characteristics and relative bioavailability of supplemental organic copper sources for poultry. *J. Anim. Sci.* **2001**, *79 5*, 1132-1141.
27. Rong-fu, G.; Kelin, C.; Xi, Z. Bioavailability of copper from tribasic copper chloride, copper amino acid chelate or copper proteinate for broilers. In Proceedings of Materials Science.

28. Miles, R.; Henry, P.; Sampath, V.; Shivazad, M.; Comer, C. Relative Bioavailability of Novel Amino Acid Chelates of Manganese and Copper for Chicks. *J. Appl. Poult. Res* **2003**, *12*, 417-423, doi:10.1093/japr/12.4.417.
29. Wang, Z.; Cerrate, S.; Coto, C.; Yan, F.; Waldroup, P. Evaluation of Mintrex® copper as source of copper in broiler diets. *Int. J. Poult. Sci.* **2007**, *6*, 308-313, doi:10.3923/ijps.2007.308.313.
30. McNaughton, J.L.; Day, E.J.; Dilworth, B.C.; Lott, B.D. Iron and copper availability from various sources. *Poult. Sci.* **1974**, *53 4*, 1325-1330.
31. Liu, S.; Lu, L.; Li, S.; Xie, J.; Zhang, L.; Wang, R.; Luo, X. Copper in organic proteinate or inorganic sulfate form is equally bioavailable for broiler chicks fed a conventional corn-soybean meal diet. *Biol. Trace Elem. Res.* **2012**, *147*, 142-148, doi:10.1007/s12011-012-9329-5.
32. Norvell, M.J.; Thomas, M.C.; Goatcher, W.D.; Gable, D.A.; Calvert, C.C. Some effects of high dietary levels of various salts of copper in broiler chickens. In Proceedings of University of Missouri's Annual Conference on Trace Substances in Environmental Health, Columbia, MO, USA, 11 June 1974; pp. 367-372.
33. Ledoux, D.R.; Ammerman, C.B.; Miles, R.D. Biological availability of copper sources for broiler chicks. *Poultry Sci.* **1987**, *66*.
34. Ledoux, D.R.; Henry, P.R.; Ammerman, C.B.; Rao, P.V.; Miles, R.D. Estimation of the relative bioavailability of inorganic copper sources for chicks using tissue uptake of copper. *J. Anim. Sci.* **1991**, *69 1*, 215-222.
35. Aoyagi, S.; Baker, D.H. Bioavailability of copper in analytical-grade and feed-grade inorganic copper sources when fed to provide copper at levels below the chick’s requirement. *Poult. Sci.* **1993a**, *72*, 1075-1083, doi:https://doi.org/10.3382/ps.0721075.
36. Zanetti, M.A.; Henry, P.R.; Ammerman, C.B.; Miles, R.D. Estimation of the relative bioavailability of copper sources in chicks fed on conventional dietary amounts. *Br. Poult. Sci.* **1991**, *32*, 583-588, doi:10.1080/00071669108417383.
37. Miles, R.D.; O'Keefe, S.F.; Henry, P.R.; Ammerman, C.B.; Luo, X.G. The effect of dietary supplementation with copper sulfate or tribasic copper chloride on broiler performance, relative copper bioavailability, and dietary prooxidant activity. *Poult. Sci.* **1998**, *77*, 416-425, doi:10.1093/ps/77.3.416.
38. Luo, X.G.; Ji, F.; Lin, Y.X.; Steward, F.A.; Lu, L.; Liu, B.; Yu, S.X. Effects of dietary supplementation with copper sulfate or tribasic copper chloride on broiler performance, relative copper bioavailability, and oxidation stability of vitamin E in feed. *Poult. Sci.* **2005**, *84*, 888-893, doi:10.1093/ps/84.6.888.
39. Liu, Z.; Bryant, M.; Roland, D.A. Layer Performance and Phytase Retention as Influenced by Copper Sulfate Pentahydrate and Tribasic Copper Chloride. *J. Appl. Poult. Res* **2005**, *14*, 499-505, doi:10.1093/japr/14.3.499.
40. Norvell, M.J.; Gable, D.A.; Thomas, M.C. Effects of feeding high levels of various copper salts to broiler chickens. In Proceedings of Annual conference on trace substances in environmental health Columbia, MO, USA.
41. Miles, R.D.; Ammerman, C.B.; Henry, P.R.; O' Keefe, S.F. The influence of high dietary copper supplementation from two feed grade inorganic sources on broiler performance, tissue copper accumulation and dietary prooxidnt activity. *Poult. Sci.* **1994**, *73*.
42. Jackson, N.; Stevenson, M.H. A study of the effects of dietary added cupric oxide on the laying, domestic fowl and a comparison with the effects of hydrated copper sulphate. *Br. J. Nutr.* **1981**, *45*, 99-110, doi:10.1079/bjn19810082.
43. Aoyagi, S.; Hiney, K.M.; Baker, D.H. Copper bioavailability in pork liver and in various animal by-products as determined by chick bioassay. *J. Anim. Sci.* **1995**, *73*, 799-804, doi:10.2527/1995.733799x.
44. Izquierdo, O.A.; Baker, D.H. Bioavailability of copper in pig feces. *C. J. Anim. Sci.* **1986**, *66*, 1145-1148, doi:10.4141/cjas86-127.

45. Hatfield, P.G.; Swenson, C.K.; Kott, R.W.; Ansotegui, R.P.; Roth, N.J.; Robinson, B.L. Zinc and copper status in ewes supplemented with sulfate- and amino acid-complexed forms of zinc and copper. *J. Anim. Sci.* **2001**, *79*, 261-266, doi:10.2527/2001.791261x.
46. Ledoux, D.R.; Pott, E.B.; Henry, P.R.; Ammerman, C.B.; Merritt, A.M.; Madison, J.B. Estimation of the relative bioavailability of inorganic copper sources for sheep. *Nutr. Res.* **1995**, *15*, 1803-1813, doi:https://doi.org/10.1016/0271-5317(95)02049-7.
47. Macpherson, A.; Hemingway, R.G. Effects of liming and various forms of oral copper supplementation on the copper status of grazing sheep. *J. Sci. Food Agric.* **1968**, *19*, 53-56, doi:10.1002/jsfa.2740190111.
48. Luo, X.G.; Henry, P.R.; Ammerman, C.B.; Madison, J.B. Relative bioavailability of copper in a copper-lysine complex or copper sulfate for ruminants as affected by feeding regimen. *Anim. Feed Sci. Technol.* **1996**, *57*, 281-289.
49. Pal, D.T.; Gowda, N.K.; Prasad, C.S.; Amarnath, R.; Bharadwaj, U.; Suresh Babu, G.; Sampath, K.T. Effect of copper- and zinc-methionine supplementation on bioavailability, mineral status and tissue concentrations of copper and zinc in ewes. *J. Trace Elem. Med. Biol. Organ Soc. Miner. Trace Elem. (GMS)* **2010**, *24*, 89-94, doi:10.1016/j.jtemb.2009.11.007.
50. Eckert, G.; Greene, L.; Carstens, G.; Ramsey, W. Copper status of ewes fed increasing amounts of copper from copper sulfate or copper proteinate. *J. Anim. Sci.* **1999**, *77*, 244-249, doi:10.2527/1999.771244x.
51. Charmley, L.L.; Ivan, M. The relative accumulation of copper in the liver and kidneys of sheep fed corn silage supplemented with copper chloride, copper acetate or copper sulphate. *Can. J. Anim. Sci.* **1989**, *69*, 205-214, doi:10.4141/cjas89-024.
52. Dick, A. Studies on the assimilation and storage of copper in crossbred sheep. *Aus. J. Agri. Res.* **1954**, *5*, 511-555, doi:https://doi.org/10.1071/AR9540511.
53. Suttle, N.F. Effects of organic and inorganic sulphur on the availability of dietary copper to sheep. *Br. J. Nutr.* **1974b**, *32*, 559-568, doi:10.1079/bjn19740109.
54. Prince, T.J.; Hays, V.W.; Cromwell, G.L. Environmental effects of high copper pig manure on pastures for sheep. *J. Anim. Sci.* **1975**, *41*.
55. Dalgarno, A.C.; Mills, C.F. Retention by sheep of copper from aerobic digests of pig faecal slurry. *J. Agri. Sci.* **1975**, *85*, 11-18, doi:10.1017/s0021859600053363.
56. Suttle, N.F.; Price, J. The potential toxicity of copper-rich animal excreta to sheep. *Anim. Sci.* **1976**, *23*, 233-241, doi:10.1017/s0003356100031317.
57. Poole, D.B.R.; McGrath, D.; Fleming, G.A.; Moore, W. Effects of Applying Copper-Rich Pig Slurry to Grassland: 4. Sheep Feeding Experiments. *Irish J. Agric. Res.* **1990**, *29*, 35-40.
58. Dowdy, R.P.; Matrone, G. A Copper-Molybdenum Complex: Its effects and movement in the piglet and sheep. *J. Nutr.* **1968**, *95*, 197-201, doi:10.1093/jn/95.2.197.
59. Cromwell, G.L.; Lindemann, M.D.; Monegue, H.J.; Hall, D.D.; Orr, D.E., Jr. Tribasic copper chloride and copper sulfate as copper sources for weanling pigs. *J. Anim. Sci.* **1998**, *76*, 118-123, doi:10.2527/1998.761118x.
60. Apgar, G.A.; Kornegay, E.T.; Lindemann, M.D.; Notter, D.R. Evaluation of copper sulfate and a copper lysine complex as growth promoters for weanling swine. *J. Anim. Sci.* **1995**, *73*, 2640-2646, doi:10.2527/1995.7392640x.
61. Apgar, G.A.; Kornegay, E.T. Mineral balance of finishing pigs fed copper sulfate or a copper-lysine complex at growth-stimulating levels. *J. Anim. Sci.* **1996**, *74*, 1594-1600, doi:10.2527/1996.7471594x.
62. Coffey, R.D.; Cromwell, G.L.; Monegue, H.J. Efficacy of a copper-lysine complex as a growth promotant for weanling pigs. *J. Anim. Sci.* **1994**, *72*, 2880-2886, doi:10.2527/1994.72112880x.
63. Bunch, R.J.; Mccall, J.T.; Speer, V.C.; Hays, V.W. Copper supplementation for weanling pigs. *J. Anim. Sci.* **1965**, *24*, 995-1000, doi:10.2527/jas1965.244995x.

64. Lin, G.; Guo, Y.; Liu, B.; Wang, R.; Su, X.; Yu, D.; He, P. Optimal dietary copper requirements and relative bioavailability for weanling pigs fed either copper proteinate or tribasic copper chloride. *J. Anim. Sci. Biotechnol.* **2020**, *11*, 54, doi:10.1186/s40104-020-00457-y.
65. Allen, M.M.; Barber, R.S.; Braude, R.; Mitchell, K.G. Further studies on various aspects of the use of high-copper supplements for growing pigs. *Br. J. Nutr.* **1961**, *15*, 507-522, doi:10.1079/bjn19610064.
66. Buescher, R.G.; Griffin, S.A.; Bell, M.C. Copper availability to swine from Cu64 labelled inorganic compounds. *J. Anim. Sci.* **1961**, *20*, 529-531, doi:10.2527/jas1961.203529x.
67. Bunch, R.J.; Speer, V.C.; Hays, V.W.; Hawbaker, J.H.; Catron, D.V. Effects of copper sulfate, copper oxide and chlortetracycline on baby pig performance. *J. Anim. Sci.* **1961**, *20*, 723-726, doi:10.2527/jas1961.204723x.
68. Bunch, R.J.; Speer, V.C.; Hays, V.W.; McCall, J.T. Effects of high levels of copper and chlortetracycline on performance of pigs. *J. Anim. Sci.* **1963**, *22*, 56-60, doi:10.2527/jas1963.22156x.
69. Bekaert, H.; Eeckhout, W.; Buysse, F. The effect of $CuSO_4$ and CuO, the granulometric characteristics of $CuSO_4$ and a zinc supplementation on performance, carcass quality and liver copper status of pigs. *Landbouwtijdschrift* **1967**, *20*, 1571 - 1585.
70. Cromwell, G.L.; Stahly, T.S.; Monegue, H.J. Effects of source and level of copper on performance and liver copper stores in weanling pigs. *J. Anim. Sci.* **1989**, *67*, 2996-3002, doi:10.2527/jas1989.67112996x.
71. Barber, R.S.; Bowland, J.P.; Mithcell, K.G.; Porter, J.W.G. Copper sulphate and copper sulphide (CuS) as supplements for growing pigs. *Br. J. Nutr.* **1961**, *15*, 189-197, doi:10.1079/bjn19610024.
72. Bowland, J.P.; Braode, R.; Chamberlain, A.G.; Glascock, R.F.; Mitchell, K.G. The absorption, distribution and excretion of labelled copper in young pigs given different quantities, as sulphate or sulphide, orally or intravenously. *Br. J. Nutr.* **1961**, *15*, 59-72, doi:10.1079/bjn19610008.
73. Cromwell, G.L.; Clark, T.L. Effects of copper sulfate, copper sulfide and sodium sulfide on performance and copper stores of pigs. *J. Anim. Sci.* **1978**, *46*, 692-698, doi:10.2527/jas1978.463692x.
74. Bremner, I.; Dalgarno, A.C. Iron metabolism in the veal calf. The availability of different iron compounds. *Br. J. Nutr.* **1973**, *29*, 229-243, doi:10.1079/bjn19730098.
75. McGuire, S.O.; Miller, W.J.; Gentry, R.P.; Neathery, M.W.; Ho, S.Y.; Blackmon, D.M. Influence of high dietary iron as ferrous carbonate and ferrous sulfate on iron metabolism in young calves. *J. Dairy Sci.* **1985**, *68*, 2621-2628, doi:10.3168/jds.S0022-0302(85)81146-2.
76. Miller, W.J.; Gentry, R.P.; Blackmon, D.M.; Fosgate, H.H. Effects of High Dietary Iron as Ferrous Carbonate on Performance of Young Dairy Calves. *J. Dairy Sci.* **1991**, *74*, 1963-1967, doi:10.3168/jds.S0022-0302(91)78363-X.
77. Chausow, D.G.; Czarnecki-Maulden, G.L. The relative bioavailability of iron from feedstuffs of plant and animal origin to the chick. *Nutr. Res.* **1988b**, *8*, 175-185, doi:https://doi.org/10.1016/S0271-5317(88)80021-6.
78. Chausow, D.G.; Czarnecki-Maulden, G.L. The relative bioavailability of plant and animal sources of iron to the cat and chick. *Nutr. Res.* **1988a**, *8*, 1041 - 1050.
79. Fly, A.D.; Fahey, G.C.; Czarnecki-Maulden, G.L. Iron bioavailability from diets containing isolated or intact sources of lignin. *Biol. Trace Elem. Res.* **1998**, *62*, 83-100, doi:10.1007/bf02820024.
80. Pla, G.W.; Fritz, J.C. Availability of Iron. *J. Assoc. Off. Anal. Chem.* **1970**, *53*.
81. Fritz, J.C.; Pla, G.W.; Roberts, T.; Boehne, J.W.; Hove, E.L. Biological availability in animals of iron from common dietary sources. *J. Agric. Food Chem.* **1970**, *18*, 647-651, doi:10.1021/jf60170a031.
82. Deming, J.G.; Czarnecki-Maulden, G.L. Iron bioavailability in calcium and phosphorus sources. *J. Anim. Sci.* **1989**, *67*.

83. Boling, S.D.; Edwards, H.M., 3rd; Emmert, J.L.; Biehl, R.R.; Baker, D.H. Bioavailability of iron in cottonseed meal, ferric sulfate, and two ferrous sulfate by-products of the galvanizing industry. *Poult. Sci.* **1998**, *77*, 1388-1392, doi:10.1093/ps/77.9.1388.
84. Henry, P.R.; Ammerman, C.B.; Miles, R.D.; Littell, R.C. Relative bioavailability of iron in feed grade phosphates for chicks. *J. Anim. Sci.* **1992**, *70*.
85. Ma, X.Y.; Liu, S.B.; Lu, L.; Li, S.F.; Xie, J.J.; Zhang, L.Y.; Zhang, J.H.; Luo, X.G. Relative bioavailability of iron proteinate for broilers fed a casein-dextrose diet. *Poult. Sci.* **2014**, *93*, 556-563, doi:10.3382/ps.2013-03296.
86. Cao, J.; Luo, X.G.; Henry, P.R.; Ammerman, C.B.; Littell, R.C.; Miles, R.D. Effect of dietary iron concentration, age, and length of iron feeding on feed intake and tissue iron concentration of broiler chicks for use as a bioassay of supplemental iron sources. *Poult. Sci.* **1996**, *75*, 495-504, doi:https://doi.org/10.3382/ps.0750495.
87. Zhang, L.Y.; Lu, L.; Luo, X.G. The chemical characteristics of organic iron sources and their relative bioavailabilities for broilers fed a conventional corn-soybean meal diet. *J. Anim. Sci.* **2016**, *94*, 2378-2396, doi:10.2527/jas.2016-0297.
88. Amine, E.K.; Neff, R.; Hegsted, D.M. Biological estimation of available iron using chicks or rats. *J. Agric. Food Chem.* **1972**, *20*, 246-251, doi:10.1021/jf60180a051.
89. Pla, G.W.; Fritz, J.C. Collaborative Study of the Hemoglobin Repletion Test in Chicks and Rats for Measuring Availability of Iron. *J. Assoc. Off. Anal. Chem.* **1971**, *54*, 13-17, doi:10.1093/jaoac/54.1.13.
90. Pennell, M.D.; Davies, M.I.; Rasper, J.; Motzok, I. Biological availability of iron supplements for rats, chicks and humans. *J. Nutr.* **1976**, *106*, 265-274, doi:10.1093/jn/106.2.265.
91. Elvehjem, C.A.; Hart, E.B. The relation of iron and copper to hemoglobin synthesis in the chick. *J. Biol. Chem.* **1929**, *84*, 131-141.
92. Poitevint, A.L. Determination of the true biological availability of ferrous carbonate. *Feedstuffs* **1979**, *51*, 31.
93. Moore, J.R.; Hitchcock, J.P.; Miller, L.C. Effect of nickel level, iron level and iron form on performance, hematological and mineral parameters of broiler chicks. *J. Anim. Sci.* **1988**, *66*, 326.
94. Ammerman, C.B.; Henry, P.R.; Miles, R.D.; Littell, R.C. Feed grade phosphates and iron sulfates as sources of iron for animals. In Proceedings of Trace Elements in Man and Animals - TEMA 8, Gersdorf, Germany.
95. Pla, G.W.; Harrison, B.N.; Fritz, J.C. Comparison of Chicks and Rats as Test Animals for Studying Bioavailability of Iron, with Special Reference to Use of Reduced Iron in Enriched Bread. *J. Assoc. Off. Anal. Chem.* **1973**, *56*, 1369-1373, doi:10.1093/jaoac/56.6.1369.
96. Aoyagi, S.; Baker, D.H. Iron requirement of chicks fed a semipurified diet based on casein and soy protein concentrate. *Poult. Sci.* **1995**, *74*, 412-415, doi:10.3382/ps.0740412.
97. Biehl, R.R.; Emmert, J.L.; Baker, D.H. Iron bioavailability in soybean meal as affected by supplemental phytase and 1 alpha-hydroxycholecalciferol. *Poult. Sci.* **1997**, *76*, 1424-1427, doi:10.1093/ps/76.10.1424.
98. Van Ravenswaay, R.O.; Henry, P.; Ammerman, C. Effects of time and dietary iron on tissue iron concentration as an estimate of relative bioavailability of supplemental iron sources for ruminants. *Anim. Feed Sci. Technol.* **2001**, *90*, 185-198, doi:10.1016/s0377-8401(01)00208-5.
99. Harmon, B.G.; Becker, D.E.; Jensen, A.H. Efficacy of Ferric Ammonium Citrate in Preventing Anemia in Young Swine. *J. Anim. Sci.* **1967**, *26*, 1051-1053, doi:10.2527/jas1967.2651051x.
100. Miller, E.R.; Parsons, M.J.; Ullrey, D.E.; Ku, P.K. Bioavailability of Iron from Ferric Choline Citrate and a Ferric Copper Cobalt Choline Citrate Complex for Young Pigs. *J. Anim. Sci.* **1981**, *52*, 783-787, doi:10.2527/jas1981.524783x.
101. Ullrey, D.E.; Miller, E.R.; Hitchcock, J.P.; Ku, P.K.; Covert, R.L.; Hegenauer, J.; Saltman, P. Oral ferric citrate vs ferrous sulfate for prevention of baby pig anemia. *Michigan Agric. Exp. Sta. Rep.* **1973**, *232*, 34-38.

102. Pickett, R.A.; Plumlee, M.P.; Beeson, W.M. Availability of dietary iron in diffent compounds for young pigs. *J. Anim. Sci.* **1961**, *20*, 946.
103. Anderson, T.A.; Filer, L.J., Jr.; Fomon, S.J.; Andersen, D.W.; Nixt, T.L.; Rogers, R.R.; Jensen, R.L.; Nelson, S.E. Bioavailability of different sources of dietary iron fed to Pitman-Moore miniature pigs. *J. Nutr.* **1974**, *104*, 619-628, doi:10.1093/jn/104.5.619.
104. Ammerman, C.B.; Standish, J.F.; Holt, C.E.; Houser, R.H.; Miller, S.M.; Combs, G.E. Ferrous carbonates as sources of iron for weanling pigs and rats. *J. Anim. Sci.* **1974**, *38*, 52-58, doi:10.2527/jas1974.38152x.
105. Harmon, B.G.; Hoge, D.E.; Jensen, A.H.; Baker, D.H. Efficacy of Ferrous Carbonate as a Hematinic for Young Swine. *J. Anim. Sci.* **1969**, *29*, 706-710, doi:10.2527/jas1969.295706x.
106. Miller, E.R. Biological availability of iron in iron supplements. *Feedstuffs* **1978**, *50*, 20.
107. Kuznetsov, S.G. Biological availability of iron from different chemical compounds for piglets. *Soviet Agric. Sci.* **1987**, *12*, 32 -36.
108. Spears, J.W.; Schoenherr, W.D.; Kegley, E.B.; Flowers, W.L.; Alhusen, H.D. Efficacy of iron methionine as a source of iron for nursing pigs. *J. Anim. Sci.* **1992**, *70*, 243.
109. Lewis, A.; Miller, P.S.; Wolverton, C. *Bioavailability of Iron in two different sources for weanling pigs*; Animal science department, University of Nebraska: Lincoln, USA, 1996.
110. Brady, P.S.; Ku, P.K.; Ullrey, D.E.; Miller, E.R. Evaluation of an amino acid-iron chelate hematinic for the baby pig. *J. Anim. Sci.* **1978**, *47*, 1135-1140, doi:10.2527/jas1978.4751135x.
111. Kim, S.W.; Hulbert, L.E.; Rachuonyo, H.A.; McGlone, J.J. Relative Availability of Iron in Mined Humic Substances for Weanling Pigs. *Asian-Australas. J. Anim. Sci.* **2004**, *17*, 1266-1270.
112. Kornegay, E.T. Availability of Iron Contained in Defluorinated Phosphate. *J. Anim. Sci.* **1972**, *34*, 569-572, doi:10.2527/jas1972.344569x.
113. Schaible, P.; Bandemer, S.L.; Davidson, J. The manganese content of feedstufls and its relation to poultry nutrition. In *Manganese content feed. its relat. poult. nutr.*, Michigan State Coll. Agric. Exp. Stat.: 1938; p 32.
114. Gallup, W.D.; Norris, L.C. The Amount of Manganese Required to Prevent Perosis in the Chick. *Poult. Sci.* **1939**, *18*, 76-82, doi:https://doi.org/10.3382/ps.0180076.
115. Scheideler, S.E. Interaction of dietary calcium, manganese, and manganese source (Mn oxide or Mn methionine complex) on chick performance and manganese utilization. *Biol. Trace Elem. Res.* **1991**, *29*, 217-228, doi:10.1007/bf03032679.
116. Fly, A.D.; Izquierdo, O.A.; Lowry, K.R.; Baker, D.H. Manganese bioavailability in a Mn-methionine chelate. *Nutr. Res.* **1989**, *9*, 901-910.
117. Henry, P.R.; Ammerman, C.B.; Miles, R.D. Relative bioavailability of manganese in a manganese-methionine complex for broiler chicks. *Poult. Sci.* **1989**, *68*, 107-112, doi:10.3382/ps.0680107.
118. Black, J.R.; Ammerman, C.B.; Henry, P.R.; Miles, R.D. Biological availability of manganese sources and effects of high dietary manganese on tissue mineral composition of broiler-type chicks. *Poult. Sci.* **1984**, *63*, 1999-2006, doi:10.3382/ps.0631999.
119. Watson, L.T.; Ammerman, C.B.; Miller, S.M.; Harms, R.H. Biological Assay of Inorganic Manganese for Chicks. *Poult. Sci.* **1970**, *49*, 1548-1554, doi:https://doi.org/10.3382/ps.0491548.
120. Wong-Valle, J.; Ammerman, C.B.; Henry, P.R.; Rao, P.V.; Miles, R.D. Bioavailability of manganese from feed grade manganese oxides for broiler chicks. *Poult. Sci.* **1989a**, *68*, 1368-1373, doi:10.3382/ps.0681368.
121. Watson, L.T.; Ammerman, C.B.; Miller, S.M.; Harms, R.H. Biological availability to chicks of manganese from different inorganic sources. *Poult. Sci.* **1971**, *50 6*, 1693-1700.

122. Baker, D.H.; Halpin, K.M. Efficacy of a manganese-protein chelate compared with that of manganese sulfate for chicks. *Poult. Sci.* **1987**, *66*, 1561-1563, doi:10.3382/ps.0661561.
123. Smith, M.O.; Sherman, I.L.; Miller, L.C.; Robbins, K.R.; Halley, J.T. Relative biological availability of manganese from manganese proteinate, manganese sulfate, and manganese monoxide in broilers reared at elevated temperatures. *Poult. Sci.* **1995**, *74*, 702-707, doi:10.3382/ps.0740702.
124. Wang, F.; Lu, L.; Li, S.; Liu, S.; Zhang, L.; Yao, J.; Luo, X. Relative bioavailability of manganese proteinate for broilers fed a conventional corn-soybean meal diet. *Biol. Trace Elem. Res.* **2012**, *146*, 181-186, doi:10.1007/s12011-011-9238-z.
125. Saldanha, M.M.; Araújo, I.C.S.; Triguineli, M.V.; Vaz, D.P.; Ferreira, F.N.A.; Albergaria, J.D.S.; Fontes, D.O.; Lara, L.J.C. Relative bioavailability of manganese in relation to proteinate and sulfate sources for broiler chickens from one to 20 d of age. *Poult. Sci.* **2020**, *99*, 5647-5652, doi:https://doi.org/10.1016/j.psj.2020.05.006.
126. Li, S.; Luo, X.; Liu, B.; Crenshaw, T.D.; Kuang, X.; Shao, G.; Yu, S. Use of chemical characteristics to predict the relative bioavailability of supplemental organic manganese sources for broilers1. *J. Anim. Sci.* **2004**, *82*, 2352-2363, doi:10.2527/2004.8282352x.
127. Luo, X.; Li, S.; Liu, B.; Bu, Y.; Kuang, X.; Yu, S. Bioavailabilities of manganese sources based on heart manganese-containing superoxide dismutase gene expression for broilers. *Wei Sheng Yan Jiu = J. Hyg. Res.* **2004**, *33*, 681-686.
128. Li, S.F.; Luo, X.G.; Lu, L.; Crenshaw, T.D.; Bu, Y.; Liu, B.; Kuang, X.; Shao, G.Z.; Yu, S.X. Bioavailability of organic manganese sources in broilers fed high dietary calcium. *Anim. Feed Sci.Technol.* **2005**, *123*, 703-715.
129. Yan, F.; Waldroup, P. Evaluation of Mintrex® manganese as a source of manganese for young broilers. *Int. J. Poult. Sci* **2006**, *5*, 708-713.
130. Southern, L.L.; Baker, D.H. Excess manganese ingestion in the chick. *Poult. Sci.* **1983**, *62*, 642-646, doi:10.3382/ps.0620642.
131. Henry, P.R.; Ammerman, C.B.; Miles, R.D. Bioavailability of manganese sulfate and manganese monoxide in chicks as measured by tissue uptake of manganese from conventional dietary levels. *Poult. Sci.* **1986**, *65*, 983-986, doi:10.3382/ps.0650983.
132. Luo, X. Studies on the optimal manganese level and its bioavailability in a practical diet for broiler chicks. Chinese Academy of Agricultural Science, Beijing, China, 1989.
133. Korol, W.; Wójcik, S.; Matyka, S.; Hansen, T.S. Availability of manganese from different manganese oxides and their effect on performance of broiler chickens. *J. Anim. Feed Sci.* **1996**, *5*, 273-279, doi:10.22358/jafs/69606/1996.
134. Henry, P.; Ammerman, C.; Miles, R. Bioavailability of manganese monoxide and manganese dioxide for broiler chicks. *Nutr. Rep. Int.* **1987**.
135. Brooks, M.A.; Grimes, J.L.; Lloyd, K.E.; Valdez, F.R.; Spears, J.W. Relative bioavailability in chicks of manganese from manganese propionate. *J. Appl. Poult. Res.* **2012**, *21*, 126-130.
136. Ao, T.; Pierce, J.L.; Pescatore, A.J.; Ford, M.J.; Cantor, A.H.; Dawson, K.A.; Paul, M. Evaluation of organic Mn (Bioplex Mn®) as a Mn source for chicks. *Poult. Sci.* **2008**, *87*, 172-173.
137. Black, J.R.; Ammerman, C.B.; Henry, P.R. Effects of high dietary manganese as manganese oxide or manganese carbonate in sheep. *J. Anim. Sci.* **1985**, *60*, 861-866, doi:10.2527/jas1985.603861x.
138. Wong-Valle, J.; Henry, P.R.; Ammerman, C.B.; Rao, P.V. Estimation of the relative bioavailability of manganese sources for sheep. *J. Anim. Sci.* **1989b**, *67*, 2409-2414, doi:10.2527/jas1989.6792409x.
139. Henry, P.R.; Ammerman, C.B.; Littell, R.C. Relative bioavailability of manganese from a manganese-methionine complex and inorganic sources for ruminants. *J. Dairy Sci.* **1992**, *75*, 3473-3478, doi:10.3168/jds.S0022-0302(92)78123-5.
140. Kayongo-Male, H.; Ullrey, D.E.; Miller, E.R. Manganese (Mn) nutrition of the pig. 2 The availability of Mn from different sources to the growing pig. *Bull. Anim. Health Prod. Africa* **1980**, *28*, 145-153.

141. Neathery, M.W.; Rachmat, S.; Miller, W.J.; Gentry, R.P.; Blackmon, D.M. Effect of Chemical Form of Orally Administered 65Zn on Absorption and Metabolism in Cattle. *Proc. Soc. Exp. Biol. Med.* **1972**, *139*, 953-956, doi:10.3181/00379727-139-36274.
142. Kincaid, R.L. Biological availability of zinc from inorganic sources with excess dietary calcium. *J. Dairy Sci.* **1979**, *62*, 1081-1085, doi:10.3168/jds.S0022-0302(79)83378-0.
143. Engle, T.E.; Nockels, C.F.; Kimberling, C.V.; Weaber, D.L.; Johnson, A.B. Zinc repletion with organic or inorganic forms of zinc and protein turnover in marginally zinc-deficient calves. *J. Anim. Sci.* **1997**, *75*, 3074-3081, doi:10.2527/1997.75113074x.
144. Kincaid, R.L., A.S. Hodgson, R.E. Riley, Jr. and J.D. Conrath. Supplementation of diets for lactating cows with zinc as zinc oxide and zinc methionine. *J. Dairy Sci.* **1984**, *67*, 103 [Abstract].
145. Greene, L.W.; Lunt, D.K.; Byers, F.M.; Chirase, N.K.; Richmond, C.E.; Knutson, R.E.; Schelling, G.T. Performance and carcass quality of steers supplemented with zinc oxide or zinc methionine. *J. Anim. Sci.* **1988**, *66*, 1818-1823, doi:10.2527/jas1988.6671818x.
146. Spears, J.W. Zinc Methionine for Ruminants: Relative Bioavailability of Zinc in Lambs and Effects of Growth and Performance of Growing Heifers. *J. Anim. Sci.* **1989**, *67*, 835-843, doi:10.2527/jas1989.673835x.
147. Spears, J.W.; Kegley, E.B. Effect of zinc and manganese methionine on performance of beef cows and calves. *J. Anim. Sci.* **1991**, *69*, 59 [Abstract].
148. Miller, W.J.; Blackmon, D.M.; Hiers, J.M., Jr.; Fowler, P.R.; Clifton, C.M.; Gentry, R.P. Effects of adding two forms of supplemental zinc to a practical diet on skin regeneration in Holstein heifers and evaluation of a procedure for determining rate of wound healing. *J. Dairy Sci.* **1967**, *50*, 715-721, doi:10.3168/jds.S0022-0302(67)87499-X.
149. Miller, W.J.; Blackmon, D.M.; Gentry, R.P.; Pate, F.M. Effects of High but Nontoxic Levels of Zinc in Practical Diets on 65Zn and Zinc Metabolism in Holstein Calves. *J. Nutr.* **1970**, *100*, 893-902, doi:10.1093/jn/100.8.893.
150. Kennedy, D.W., W.M. Craig, L.L. Southern and M. Engstrom. Ruminal partitioning of zinc in steers fed a polysaccharide complex of zinc or zinc oxide. *J. Anim. Sci.* **1988**, *66*, 462.
151. Hempe, J.M. Zinc bioavailability in the chick [Doctor Degree Thesis Dissertation]. University of Missouri, Columbia, 1987.
152. Hortin, A.E.; Bechtel, P.J.; Baker, D.H. Efficacy of pork loin as a source of zinc and effect of added cysteine on zinc bioavailability. *J. Food Sci.* **1991**, *56*, 1505-1507, doi:https://doi.org/10.1111/j.1365-2621.1991.tb08626.x.
153. O'Dell, B.L.; Burpo, C.E.; Savage, J.E. Evaluation of Zinc Availability in Foodstuffs of Plant and Animal Origin. *J. Nutr.* **1972**, *102*, 653-660, doi:10.1093/jn/102.5.653.
154. Edwards, H.M.; Baker, D.H. Zinc bioavailability in soybean meal. *J. Anim. Sci.* **2000**, *78*, 1017-1021, doi:10.2527/2000.7841017x.
155. Edwards, H.M., 3rd; Boling, S.D.; Emmert, J.L.; Baker, D.H. Bioavailability of zinc in two zinc sulfate by-products of the galvanizing industry. *Poult. Sci.* **1998**, *77*, 1546-1549, doi:10.1093/ps/77.10.1546.
156. Edwards, H.M., Jr. The availability to chicks of zinc in various compounds and ores. *J. Nutr.* **1959**, *69*, 306-308, doi:10.1093/jn/69.3.306.
157. Aoyagi, S.; Hiney, K.M.; Baker, D.H. Estimates of zinc and iron bioavailability in pork liver and the effect of sex of pig on the bioavailability of copper in pork liver fed to male and female chicks. *J. Anim. Sci.* **1995**, *73*, 793-798, doi:10.2527/1995.733793x.
158. O'Dell, B.L.; Savage, J.E. Effect of phytic acid on zinc availability. *Proc. Soc. Exp. Biol. Med.* **1960**, *103*, 304-306, doi:10.3181/00379727-103-25498.
159. Edwards, H.M.; Baker, D.H. Bioavailability of zinc in several sources of zinc oxide, zinc sulfate, and zinc metal. *J. Anim. Sci.* **1999**, *77*, 2730-2735, doi:10.2527/1999.77102730x.

160. Cao, J.; Henry, P.R.; Guo, R.; Holwerda, R.A.; Toth, J.P.; Littell, R.C.; Miles, R.D.; Ammerman, C.B. Chemical characteristics and relative bioavailability of supplemental organic zinc sources for poultry and ruminants. *J. Anim. Sci.* **2000**, *78*, 2039-2054, doi:10.2527/2000.7882039x.
161. Pensack, J.; Henson, J.; Bogdonoff, P. The effects of calcium and phosphorus on the zinc requirements of growing chickens. In Proceedings of Poultry Science; pp. 1232-1233.
162. Roberson, R.H.; Schaible, P.J. The Availability to the Chick of Zinc as the Sulfate, Oxide or Carbonate. *Poult. Sci.* **1960a**, *39*, 835-837, doi:https://doi.org/10.3382/ps.0390835.
163. Roberson, R.H.; Schaible, P.J. The Tolerance of Growing Chicks for High Levels of Different Forms of Zinc. *Poult. Sci.* **1960b**, *39*, 893-896, doi:https://doi.org/10.3382/ps.0390893.
164. Roberson, R.H.; Schaible, P.J. The Zinc Requirement of the Chick. *Poult. Sci.* **1958**, *37*, 1321-1323, doi:https://doi.org/10.3382/ps.0371321.
165. Yu, L.; Yi, J.; Chen, Y.; Huang, M.; Zhu, N. Relative Bioavailability of Broiler Chickens Fed with Zinc Hydroxychloride and Sulfate Sources for Corn-Soybean Meal. *Biol. Trace Elem. Res.* **2021**, *200*, 1-12, doi:10.1007/s12011-021-03013-5.
166. Hill, D.A.; Peo, E.R., Jr.; Lewis, A.J. Effect of zinc source and picolinic acid on 65Zn uptake in an in vitro continuous-flow perfusion system for pig and poultry intestinal segments. *J. Nutr.* **1987**, *117*, 1704-1707, doi:10.1093/jn/117.10.1704.
167. Pimentel, J.L.; Cook, M.E.; Greger, J.L. Research Note: Bioavailability of Zinc-Methionine for Chicks. *Poult. Sci.* **1991**, *70*, 1637-1639.
168. Liu, S.; Li, S.; Lu, L.; Xie, J.; Zhang, L.; Wang, R.; Luo, X. The effectiveness of zinc proteinate for chicks fed a conventional corn-soybean meal diet. *J. Appl. Poult. Res.* **2013**, *22*, 396-403, doi:10.3382/japr.2012-00564.
169. Li, W.-x.; Ma, X.-y.; Lu, L.; Zhang, L.-y.; Luo, X.-g. Relative bioavailability of tribasic zinc sulfate for broilers fed a conventional corn-soybean meal diet. *J. Integ. Agric.* **2015**, *14*, 2042-2049, doi:https://doi.org/10.1016/S2095-3119(15)61033-4.
170. Świątkiewicz, S.; Koreleski, J.; Zhong, D.Q. The bioavailability of zinc from inorganic and organic sources in broiler chickens as affected by addition of phytase. *J. Anim. Feed Sci.* **2001**, *10*, 317-328, doi:10.22358/jafs/67987/2001.
171. Star, L.; van der Klis, J.D.; Rapp, C.; Ward, T.L. Bioavailability of organic and inorganic zinc sources in male broilers. *Poult. Sci.* **2012**, *91*, 3115-3120, doi:10.3382/ps.2012-02314.
172. Huang, Y.L.; Lu, L.; Li, S.F.; Luo, X.G.; Liu, B. Relative bioavailabilities of organic zinc sources with different chelation strengths for broilers fed a conventional corn-soybean meal diet1. *J. Anim. Sci.* **2009**, *87*, 2038-2046, doi:10.2527/jas.2008-1212.
173. Huang, Y.; Lu, L.; Xie, J.; Li, S.; Li, X.; Liu, S.; Zhang, L.; Xi, L.; Luo, X. Relative bioavailabilities of organic zinc sources with different chelation strengths for broilers fed diets with low or high phytate content. *Anim. Feed Sci. Technol.* **2013**, *179*, 144-148.
174. Sandoval, M.; Henry, P.R.; Ammerman, C.B.; Miles, R.D.; Littell, R.C. Relative bioavailability of supplemental inorganic zinc sources for chicks. *J. Anim. Sci.* **1997**, *75*, 3195-3205, doi:10.2527/1997.75123195x.
175. Batal, A.B.; Parr, T.M.; Baker, D.H. Zinc bioavailability in tetrabasic zinc chloride and the dietary zinc requirement of young chicks fed a soy concentrate diet. *Poult. Sci.* **2001**, *80*, 87-90, doi:10.1093/ps/80.1.87.
176. Wedekind, K.J.; Baker, D.H. Zinc bioavailability in feed-grade sources of zinc. *J. Anim. Sci.* **1990**, *68*, 684-689, doi:10.2527/1990.683684x.
177. Wedekind, K.J. The bioavailability of zinc-methionine relative to zinc sulfate is affected by calcium level. *Poult. Sci.* **1994**, *73*, 114.

178. Cao, J.; Henry, P.R.; Davis, S.R.; Cousins, R.J.; Miles, R.D.; Littell, R.C.; Ammerman, C.B. Relative bioavailability of organic zinc sources based on tissue zinc and metallothionein in chicks fed conventional dietary zinc concentrations. *Anim. Feed Sci. Tech.* **2002**, *101*, 161-170, doi:https://doi.org/10.1016/S0377-8401(02)00051-2.
179. Suo, H.; Lu, L.; Zhang, L.; Zhang, X.; Li, H.; Lu, Y.; Luo, X. Relative bioavailability of zinc-methionine chelate for broilers fed a conventional corn-soybean meal diet. *Biol. Trace Elem. Res.* **2015**, *165*, 206-213, doi:10.1007/s12011-015-0252-4.
180. Wedekind, K.J.; Hortin, A.E.; Baker, D.H. Methodology for assessing zinc bioavailability: efficacy estimates for zinc-methionine, zinc sulfate, and zinc oxide. *J. Anim. Sci.* **1992**, *70*, 178-187, doi:10.2527/1992.701178x.
181. Richards, J.; Fisher, P.; Evans, J.; Wedekind, K. Greater bioavailability of chelated compared with inorganic zinc in broiler chicks in the presence or absence of elevated calcium and phosphorus. *Open Access Anim. Physiol.* **2015**, *7*, 97-110, doi:10.2147/oaap.s83845.
182. Sahraei, M.; Janmmohamadi, H.; Taghizadeh, A.; Ali Moghadam, G.; Abbas Rafat, S. Estimation of the Relative Bioavailability of Several Zinc Sources for Broilers Fed a Conventional Corn-Soybean Meal Diet. *J. Poult. Sci.* **2013**, *50*, 53-59, doi:10.2141/jpsa.0120022.
183. Brooks, M.A.; Grimes, J.L.; Lloyd, K.E.; Verissimo, S.; Spears, J.W. Bioavailability in chicks of zinc from zinc propionate. *J. Appl. Poult. Res.* **2013**, *22*, 153-159, doi:https://doi.org/10.3382/japr.2012-00525.
184. Ao, T.; Pierce, J.L.; Power, R.F.G.; Dawson, K.A.; Pescatore, A.J.; Cantor, A.H.; Ford, M.J. Evaluation of Bioplex Zn® as an organic zinc source for chicks. *Int. J. Poult. Sci.* **2006**, *5*, 808-811.
185. Vohra, P.; Kratzer, F.H. Influence of various phosphates and other complexing agents on the availability of zinc for turkey poults. *J. Nutr.* **1966**, *89*, 106-112, doi:10.1093/jn/89.1.106.
186. Sullivan, T.W. The availability of zinc in various compounds to broad breasted bronze pouhs. *Poult. Sci.* **1961**, *40*, 340.
187. Kratzer, F.H.; Allred, J.B.; Davis, P.N.; Marshall, B.J.; Vohra, P. The effect of autoclaving soybean protein and the addition of ethylenediaminetetracetic acid on the biological availability of dietary zinc for turkey poults. *J. Nutr.* **1959**, *68*, 313-322, doi:10.1093/jn/68.2.313.
188. Jones, A.O.L.; Fox, M.R.S.; Fry, B.E., Jr. In vitro assessment of zinc binding to protein foods as a potential index of zinc bioavailability Comparison of in vitro and in vivo data. *J. Agric. Food. Chem.* **1985**, *33*, 1123-1128.
189. Sandoval, M.; Henry, P.R.; Littell, R.C.; Cousins, R.J.; Ammerman, C.B. Estimation of the relative bioavailability of zinc from inorganic zinc sources for sheep. *Anim. Feed Sci.Tech.* **1997**, *66*, 223-235, doi:https://doi.org/10.1016/S0377-8401(96)01103-0.
190. Ho, S.K.; Hidroglou, M. Effects of dietary chelated and sequestered zinc and zinc sulfate on growing lambs fed a purified diet *Can. J. Anim. Sci.* **1977**, *57*, 93-99, doi:10.4141/cjas77-011.
191. Smith, B.L.; Embling, P.P. The influence of chemical form of zinc on the effects of toxic intraruminal doses of zinc to sheep. *J. Appl. Toxicol.* **1984**, *4*, 92-96, doi:10.1002/jat.2550040207.
192. Grešáková, Ľ.; Tokarčíková, K.; Čobanová, K. Bioavailability of Dietary Zinc Sources and Their Effect on Mineral and Antioxidant Status in Lambs. *Agriculture* **2021**, *11*, 1093.
193. Rojas, L.X.; McDowell, L.R.; Cousins, R.J.; Martin, F.G.; Wilkinson, N.S.; Johnson, A.B.; Velasquez, J.B. Relative bioavailability of two organic and two inorganic zinc sources fed to sheep. *J. Anim. Sci.* **1995**, *73*, 1202-1207, doi:10.2527/1995.7341202x.
194. Swinkels, J.W.G.M.; Kornegay, E.T.; Zhou, W.; Lindemann, M.D.; Webb, K.E., Jr.; Verstegen, M.W.A. Effectiveness of a zinc amino acid chelate and zinc sulfate in restoring serum and soft Tissue zinc concentrations when fed to zinc-depleted pigs. *J. Anim. Sci.* **1996**, *74*, 2420-2430, doi:10.2527/1996.74102420x.
195. Hap, I.; Zeman, L. The effect of the use of various zinc sources on zinc availability in piglets. *Zivocisma-Vyroba* **1994**, *39*, 343-349.

196. Zhang, B.; Guo, Y. Beneficial effects of tetrabasic zinc chloride for weanling piglets and the bioavailability of zinc in tetrabasic form relative to ZnO. *Anim. Feed Sci. Technol.* **2007**, *135*, 75-85, doi:https://doi.org/10.1016/j.anifeedsci.2006.06.006.
197. Hahn, J.D.; Baker, D.H. Growth and plasma zinc responses of young pigs fed pharmacologic levels of zinc. *J. Anim. Sci.* **1993**, *71*, 3020-3024, doi:10.2527/1993.71113020x.
198. Cheng, J.; Kornegay, E.T.; Schell, T. Influence of dietary lysine on the utilization of zinc from zinc sulfate and a zinc-lysine complex by young pigs. *J. Anim. Sci.* **1998**, *76*, 1064-1074, doi:10.2527/1998.7641064x.
199. Schell, T.C.; Kornegay, E.T. Zinc concentration in tissues and performance of weanling pigs fed pharmacological levels of zinc from ZnO, Zn-methionine, Zn-lysine, or ZnSO4. *J. Anim. Sci.* **1996**, *74*, 1584-1593, doi:10.2527/1996.7471584x.
200. Wedekind, K.J.; Lewis, A.J.; Giesemann, M.A.; Miller, P.S. Bioavailability of zinc from inorganic and organic sources for pigs fed corn-soybean meal diets. *J. Anim. Sci.* **1994**, *72*, 2681-2689, doi:10.2527/1994.72102681x.
201. Miller, E.R.; Ku, P.K.; Hitchcock, J.P.; Magee, W.T. Availability of Zinc from Metallic Zinc Dust for Young Swine. *J. Anim. Sci.* **1981**, *52*, 312-315, doi:10.2527/jas1981.522312x.
202. Hill, D.A.; Peo, E.R., Jr.; Lewis, A.J.; Crenshaw, J.D. Zinc-Amino Acid Complexes for Swine. *J. Anim. Sci.* **1986**, *63*, 121-130, doi:10.2527/jas1986.631121x.
203. Dourmad, J.-Y.; Guinotte, F.; Nys, Y. Bioavailability of two sources of zinc in weanling pigs. *Anim. Res.* **2002**, *51*, 315-326.
